# Discovery of novel eGFR-associated multiple independent signals using a quasi-adaptive method

**DOI:** 10.3389/fgene.2022.997302

**Published:** 2022-10-31

**Authors:** Sahar Ghasemi, Tim Becker, Hans J. Grabe, Alexander Teumer

**Affiliations:** ^1^ Institute for Community Medicine, University Medicine Greifswald, Greifswald, Germany; ^2^ Department of Psychiatry and Psychotherapy, University Medicine Greifswald, Greifswald, Germany; ^3^ DZHK (German Center for Cardiovascular Research), Partner Site Greifswald, Greifswald, Germany; ^4^ German Center for Neurodegenerative Diseases DZNE, Site Rostock/Greifswald, Greifswald, Germany

**Keywords:** estimated glomerular filtration rate (eGFR), genome-wide association studies (GWAS), expression quantitative trait loci (eQTL), conditional association analysis, SNP-specific alpha-level, colocalization

## Abstract

A decreased estimated glomerular filtration rate (eGFR) leading to chronic kidney disease is a significant public health problem. Kidney function is a heritable trait, and recent application of genome-wide association studies (GWAS) successfully identified multiple eGFR-associated genetic loci. To increase statistical power for detecting independent associations in GWAS loci, we improved our recently developed quasi-adaptive method estimating SNP-specific alpha levels for the conditional analysis, and applied it to the GWAS meta-analysis results of eGFR among 783,978 European-ancestry individuals. Among known eGFR loci, we revealed 19 new independent association signals that were subsequently replicated in the United Kingdom Biobank (n = 408,608). These associations have remained undetected by conditional analysis using the established conservative genome-wide significance level of 5 × 10^–8^. Functional characterization of known index SNPs and novel independent signals using colocalization of conditional eGFR association results and gene expression in *cis* across 51 human tissues identified two potentially causal genes across kidney tissues: *TSPAN33* and *TFDP2*, and three candidate genes across other tissues: *SLC22A2, LRP2*, and *CDKN1C*. These colocalizations were not identified in the original GWAS. By applying our improved quasi-adaptive method, we successfully identified additional genetic variants associated with eGFR. Considering these signals in colocalization analyses can increase the precision of revealing potentially functional genes of GWAS loci.

## Introduction

Glomerular filtration rate estimated from serum creatinine (eGFR) is used to quantify kidney function and define chronic kidney disease (CKD). CKD defined by low eGFR <60 ml/min/1.73 m^2^ is strongly associated with an increased risk of major adverse clinical outcomes such as end-stage kidney disease (ESKD), cardiovascular (CV) outcomes, and mortality (Go et al., 2004; Chronic Kidney Disease PrognosisMatsushita et al., 2010; Hemmelgarn et al., 2010; Astor et al., 2011; Bello et al., 2011; Gansevoort et al., 2011; Gansevoort et al., 2013; Weiner et al., 2014; Matsushita et al., 2015). A better understanding of the biological mechanisms underlying kidney function is a prerequisite for initiating targeted treatments and reducing patient mortality, comorbidity, and associated healthcare costs. eGFR is a heritable trait with estimated h^2^ = 39%, and recent application of genome-wide association studies (GWAS) successfully identified multiple eGFR-associated genetic loci (Okada et al., 2012; Pattaro et al., 2012; Mahajan et al., 2016; Pattaro et al., 2016; Hishida et al., 2018; Kanai et al., 2018; Lee et al., 2018; Wuttke et al., 2019). Allelic heterogeneity within a GWAS locus is a common characteristic of complex traits and conditional analyses successfully identified multiple independent associations with eGFR. For instance, Gorski et al. (2017) (Gorski et al., 2017) detected 57 independent signals among the 49 loci. Morris et al. (2019) (Morris et al., 2019) delineated 127 distinct signals across the 93 loci. Hellwege et al. (2019) (Hellwege et al., 2019) discovered 18 independent signals at 15 loci, and Wuttke et al. (2019) (Wuttke et al., 2019) identified 253 independent SNPs at 228 loci explaining 7.3% of the eGFR variation.

To identify an independent signal, the SNPs of a locus are conditioned by the known significant associations. In case individual genotypes of a sample are available, the genotypes of known signals are added as covariates to the association model. Alternatively, these conditional associations can be approximated by using summary statistics and an appropriate linkage disequilibrium (LD) panel. Usually, the established genome-wide significance level of 5 × 10^–8^ was applied as a significance threshold for the conditional analysis, which is also the significance level for the primary GWAS. Since the conditional analysis is applied on a specific genomic region and not on a genome-wide scale, 5 × 10^–8^ is too conservative and implies a loss of power. In Ghasemi et al. (2021) (Ghasemi et al., 2021), we developed a quasi-adaptive method to determine SNP-specific significance levels in conditional analysis.

Although GWAS have discovered multiple eGFR-associated loci, the underlying genes that influence genetic associations have often remained unknown. Integration of GWAS signals and expression quantitative trait loci (eQTL) studies (Nica and Dermitzakis, 2013) to estimate the relation between gene expression of nearby genes and eGFR, termed colocalization (Giambartolomei et al., 2014), allows the identification of candidate genes and improves the functional interpretation of GWAS results. For instance, *FGF5*, *CDKL5*, *TPSAN33*, and *METTL10* colocalized with the eGFR-associated loci in kidney-specific tissues (Graham et al., 2019), and Wuttke et al. (2019) (Wuttke et al., 2019) detected 17 underlying genes expressed in kidney tissues including *UMOD*, *KNG1*, and *FGF5*.

Here, we improved and applied our quasi-adaptive method to the publically available GWAS meta-analysis results of 783,978 European-ancestry individuals (Wuttke et al., 2019) of the CKDGen Consortium to uncover additional independent signals for eGFR. Replication of the identified novel independent signals was conducted using individual-level participant data of the United Kingdom Biobank (UKBB) (Bycroft et al., 2018). The UKBB was not included in the primary GWAS meta-analysis, and thus represents an independent dataset for replication. We run colocalization analyses based on associations with eGFR and with gene expression (eQTLs) in *cis* across 49 human tissues included in the Genotype-Tissue Expression (GTEx) project v8^27^, as well as the microdissected human glomerular and tubulo-interstitial kidney portions from 187 individuals from the NEPTUNE study (Gillies et al., 2018). Since the presence of multiple independent signals within a GWAS locus reduces power of colocalization, we provided the colocalization analyses with conditional eGFR-association analysis and eQTL to detect potential causal genes and compared these results to the unconditional approach. Our colocalization analyses used the latest version of GTEx-v8 compared to the GTEx-v6 in the previous report of eGFR (Wuttke et al., 2019).

The emerging list of novel eGFR-associated variants and genes influencing kidney disease etiology facilitate CKD targeted treatment and prevention.

## Methods

### Additional independent eGFR-associated signals identification by quasi-adaptive method

We obtained the CKDGen Consortium 2019 eGFR-association GWAS meta-analysis results for European-ancestry (Wuttke et al., 2019) from https://ckdgen.imbi.uni-freiburg.de. The downloaded file included chromosome, position (b37), SNP rsid, effect allele, non-effect allele, effect allele frequency, beta, standard error, *p*-value, and sample size for each variant. Wuttke et al. (2019) (Wuttke et al., 2019) identified 253 independent genome-wide-significant eGFR-associated SNPs through approximate conditional analyses implemented in GCTA (Yang et al., 2011) (GCTA COJO Slct algorithm) across 228 European-ancestry-specific and replicated loci. To identify additional independent eGFR-associated secondary signals, we applied our quasi-adaptive method to the aforementioned GWAS meta-analysis with 8,885,712 genetic variants and 783,978 individuals. The method incorporated LD structure from individual-level genotype data of 15,000 randomly selected European-ancestry participants of the UKBB (Bycroft et al., 2018). The selected UKBB LD reference sample underwent the same data preparation procedure as described in (Wuttke et al., 2019) and (Teumer et al., 2019), except for the minor allele frequency (MAF) cut-off. We excluded SNPs with a MAF <0.0001. The final dataset for estimating the LD structure included 13,558 unrelated European-ancestry individuals and 36, 228, 692 genetic variants. We used the published 228 replicated index SNPs (i.e., variants with the smallest *p*-value of a locus) as the basis for applying our method (Wuttke et al., 2019). A one megabase window around the index SNPs was considered as primary loci. Overlapping loci at which two adjacent index SNPs were less than one megabase apart or with pairwise correlation $r^{2}>0.1$ were merged using the lower-bound and the upper-bound of the merged regions as new locus borders, and the SNP with the smallest *p*-value as the new index SNP. This resulted in a final list of 190 independent loci (Supplementary Table S1). All SNPs except the index SNP were considered candidate SNPs within each locus. We conducted conditional analyses on this dataset using GCTA (GCTA COJO-cond algorithm) by adjusting for the corresponding index SNP across the 190 loci. The number of tested SNPs equals to the number of candidate SNPs included in the conditional analyses across the 190 loci. As described in Ghasemi et al. (2021) (Ghasemi et al., 2021), our method prioritizes the candidate SNPs and assigns a SNP-specific α -threshold to the candidate SNPs in conditional analysis. The pairwise correlation ( $r^{2}$ ) and chromosomal distance ( d ) between the candidate SNPs and respective index SNP needed as inputs for our method were retrieved by the INTERSNP tool (Herold et al., 2009). Let $m_{2}$ be the number of tested SNPs from $N_{2}$ loci (here, $N_{2}$ = 190 with the index reflecting the analysis of secondary signals). Of note, $m_{2}$ and $N_{2}$ were named as *m* and *N* in the original paper (Ghasemi et al., 2021). The pre-weight based on $r^{2}$ ( $w_{r_{i}^{2}}$ ) with optimal $r^{2}=0.3$ and a pre-weight based on d ( $w_{d_{i}})$ which down-weighted SNPs at higher distance step-wise-strong are assigned to a candidate SNP( i ), $\left(1\leq i\leq m_{2}\;\right)$ as:

$$w_{r_{i}^{2}}=\frac{1-\left|r_{i}^{2}-0.3\right|-0.3}{\begin{array}{c} 1-0.3\\ w_{d_{i}}=\left\{ \begin{array}{c} 1\\ 0.5\\ 0.25\\ 0.125\\ 0.0625 \end{array}\right.\begin{array}{c} \begin{array}{c} \begin{array}{c} \;if\;0<d\leq 1Kb\\ \;if\;1Kb<d\leq 10Kb \end{array}\\ \;if\;10Kb<d\leq 50Kb\\ \;if\;50Kb<d\leq 100Kb \end{array}\\ \;if\;100Kb<d\leq 500Kb \end{array} \end{array}},$$

The pre-weight $w_{r_{i}^{2}}$ and $w_{d_{i}}$ are combined (with more emphasis on d than on $r^{2}$ ) by the geometric mean $w_{i}={\left(w_{d_{i}}^{k}\times w_{r_{i}^{2}}\right)}^{\frac{1}{k+1}}$ , with k=5 , to assign an optimal weight $W_{i}=\frac{w_{i}\times m_{2}}{\overset{m_{2}}{\underset{i=1}{\sum }}w_{i}}$ to SNP( i ).

The quasi-adaptive method is applied on $N_{2}$ loci, spends type *I* error rate ( α ) over $m_{2}$ candidate SNPs by incorporating $W_{i}$ into the weighted Šidák correction (Kang et al., 2009), and assigns the SNP-specific α -thresholds to SNP( i ) by $G_{i}\left(\alpha ,{\;r}^{2},\;d\;\right)$ as follows:

$$G_{i}\left(\alpha ,{\;r}^{2},\;d\;\right)=1-{\left(1-\alpha \right)}^{\frac{W_{i}}{m_{2}}},\;i=1,2,\ldots ,\;m_{2}$$ (1)

SNP( i ) is a secondary signal if the conditional *p*-value is smaller than $G_{i}\left(\alpha ,{\;r}^{2},\;d\;\right)$ .

(Ghasemi et al., 2021) showed that Equation 1 has the overall best power in detecting secondary signals while controlling the family-wise error rate (FWER) at the α -level. In our study, α was set to 0.05.

### Improved quasi-adaptive method to identify multiple independent eGFR-associated signals

The original quasi-adaptive method was developed to determine one independent signal (secondary signal) with the smallest conditional *p*-value smaller than the correspondingly assigned $G\left(\alpha ,{\;r}^{2},\;d\;\right)$ at each locus. We extended the idea from the main paper (Ghasemi et al., 2021) to identify multiple independent signals (a tertiary signal, a signal of fourth, a signal of fifth, and beyond). To detect independent tertiary signals, only loci with confirmed secondary signals (confirmed according to the quasi-adaptive method) were considered. We proceeded according to the idea of the paper (Ghasemi et al., 2021) but performed conditional analyses by adjusting for the primary index SNP and confirmed secondary signal for each locus. Let $N_{3}$ be the number of loci with confirmed secondary signals and $m_{3}$ be the number of tested SNPs from $N_{3}$ loci (i.e., excluding index SNPs and secondary signals). Of note, the number of tested SNPs is lower for tertiary signals detection than for secondary signals detection ( $m_{3}<m_{2}$ ). As described in 2.1, the LD structure was determined between the index SNP and corresponding candidate SNPs at each locus. Our method was applied on $N_{3}$ loci according to the schema described in 2.1 and the SNP-specific α -thresholds assigned to SNP( i ) by equation (2)

$$G_{i}\left(\alpha ,{\;r}^{2},\;d\;\right)=1-{\left(1-\alpha \right)}^{\frac{W_{i}}{m_{3}}},\;W_{i}=\frac{w_{i}\times m_{3}}{\overset{m_{3}}{\underset{i=1}{\sum }}w_{i}},\;i=1,2,\ldots ,\;m_{3}$$ (2)

The improved method is an iterative process that is subsequently performed to detect higher-order independent signals (applied to loci with confirmed independent signals from the previous steps) until no additional independent signals are found. Finding higher-order independent signals keeps the FWER at the α -level because only the number of tested SNPs and the LD structure have to be taken into account (as shown in Equations 1, 2, where the LD structure does not change by analyzing higher-order independent signals.

Due to the complexity of the LD structure of the major histocompatibility complex (MHC) region, this region was excluded from the search for independent signals as also in the main GWAS (Wuttke et al., 2019).

### Replication of the results in the UK biobank dataset

The novel independent eGFR-associated signals were tested for replication by conditional association analyses using the individual-level data of the UKBB (Bycroft et al., 2018) cohort. This cohort was not included in the initial GWAS of eGFR, and thus represents an independent dataset for replication. The phenotype definition, quality control, and analyses were performed using the same methods and scripts of the main GWAS (Teumer et al., 2019; Wuttke et al., 2019). As independent signals were identified from samples of European ancestry, conditional analyses were restricted to 408,608 UKBB participants of European ancestry with approximately 19 million autosomal SNPs that met the inclusion criteria of MAF ≥0.001 and imputation quality score *>* 0.3. For replication of each category of independent signals (secondary, tertiary, and beyond) across loci, a conditional analysis was conducted by including sex- and age-adjusted residual of log (eGFR), the first 15 genetic principal components, and the allele dosages of all corresponding conditioned SNPs as covariates in a mixed-model association method as implemented in BOLT-LMM, v2.3.2 (Loh et al., 2005). Within each locus, conditional analysis was performed for replication of an identified independent signal by conditioning on a known index SNP and (if present) on other known or replicated independent signals identified before the corresponding independent signal. Of note, non-replicated signals identified before the independent signal under investigation were excluded from the conditional analysis. Supplementary Table S2 shows the list of known index SNPs and known and novel independent signals with the list of covariates (SNPs) used for replication. Bonferroni correction of 0.05/9, 0.05/8, 0.05/6, 0.05/3, and 0.05, correcting for the number of tested SNPs per conditional analysis, was applied to assess the significance of the replication of secondary signals, tertiary signals, signals of fourth, signals of fifth, and signal of sixth, respectively.

### Colocalization of eGFR signals with gene expression in cis

In the first instance, colocalization analyses were run for known index SNPs and novel independent signals using unconditional eGFR association analyses in the UKBB and expression quantitative trait (eQTL) studies (Nica and Dermitzakis, 2013). eQTL were quantified from 49 human tissues included in the GTEx project v8 release (Aguet et al., 2019), and the microdissected human glomerular and tubulointerstitial kidney portions from 187 individuals from the NEPTUNE study (Gillies et al., 2018). For colocalization, the effect alleles for GWAS and eQTLs were harmonized, and tissue gene pairs with eQTL data were identified within ± 100 kilobases of the independent signals. We used the eQTL *cis* window (1-megabase window from each side of the transcriptional start site) as the region for each colocalization test. We applied colocalization by using the approximate Bayes factor computations with the default prior probability = 1 × 10^–5^ on the signals available in both GWAS and eQTL as implemented in the coloc. fast function from the R package “gtx” version 2.1.6 (https://github.com/tobyjohnson/gtx). This function provides an adaptation of Giambartolomei’s colocalization method (Giambartolomei et al., 2014).

Secondly, we re-run the colocalization analyses using conditional eGFR association analyses and the eQTL studies. Conditional analysis was performed for a known index SNP by adjusting for all known and novel independent signals and for a novel independent signal by conditioning on a known index SNP and (if present) on other known or novel independent signals within the corresponding locus. Supplementary Table S2 shows the list of covariates (SNPs) used in the eGFR association. We defined a variant as a colocalized signal (same causal variant underlying both the GWAS and eQTL association) if the posterior probability (PP) of a variant was greater than 80%.

## Results

### Novel eGFR-associated multiple conditionally independent signals

To detect additional eGFR-associated independent signals, our method was applied on 190 loci derived from the GWAS meta-analysis (Wuttke et al., 2019) (Methods and Supplementary Table S1). Our method identified in total 87 independent signals, including 53 secondary signals (Supplementary Table S3), 20 tertiary signals (Supplementary Table S4), 10 signals of fourth (Supplementary Table S5), three signals of fifth (Supplementary Table S6), and one signal of sixth (Supplementary Table S7), of which 27 were novel (Table 1). Of note, all novel SNPs were secondary or higher-order signals. We have listed the differences between the previous analysis (Wuttke et al., 2019) and our analysis in Supplementary Tables S3-S7 in a column labeled “Known”. At a locus, an SNP detected by our method was considered known (yes) if it was exactly the independent signal or in high LD ( $r^{2}>0.8$ ) with a SNP detected by Wuttke et al. (2019) (Wuttke et al., 2019). We detected 60 known loci, of which 54 loci comprised the same independent signal identified in the previous GWAS, and six loci with independent signals in high LD with the identified independent signals from the aforementioned GWAS.

**TABLE 1:** Summary of novel independent eGFR-associated signals identified by quasi-adaptive method and replication results.

—	Chr	Signal	Index	Closest gene	D [bp]	r^2^	Pos (b37)	EA	EAF	GWAS-MA			GCTA			α-threshold	Replication-UKBB		
										Effect	Se	P	Effect	Se	P		Effect	Se	P
Secondary signal	3	**rs147877018**	rs1397764	*TFDP2*	62,539	0.031	141,813,349	A	0.081	−0.0047	0.0007	1.18E-12	−0.0035	0.0007	1.13E-07	1.33E-07	−0.0042	0.0006	1.80E-11
	4	rs59664098	rs7667050	*PPARGC1A*	50,300	0.001	23,863,409	A	0.070	−0.0037	0.0007	2.30E-07	−0.0038	0.0007	8.66E-08	1.31E-07	−0.0011	0.0007	8.70E-02
	6	**rs3904600**	rs6921580	*RREB1*	−94,049	0.107	7,109,665	C	0.370	0.0027	0.0004	4.91E-14	0.0018	0.0003	9.75E-08	1.37E-07	0.0030	0.0004	1.80E-16
	7	rs12111979	rs700753	*LOC730338*	59,613	0.170	46,813,297	T	0.420	0.0004	0.0003	2.38E-01	0.0017	0.0003	8.07E-08	1.39E-07	0.0007	0.0004	4.70E-02
	8	**rs4566**	rs10086569	*SLC7A13*	−886,127	0.002	86,361,082	T	0.610	0.0020	0.0004	1.03E-08	0.0019	0.0004	5.90E-08	7.37E-08	0.0011	0.0003	1.50E-03
	12	rs2300127	rs11062167	*SLC6A13*	−49,290	0.011	315,449	T	0.570	0.0023	0.0004	1.62E-10	0.0019	0.0004	1.99E-07	2.35E-07	0.0008	0.0003	2.20E-02
	12	**rs11056376**	rs10846157	*RERG*	−17,637	0.020	15,307,394	A	0.910	0.0040	0.0007	5.43E-10	0.0034	0.0006	2.03E-07	2.36E-07	0.0021	0.0006	6.00E-04
	12	**rs3730071**	rs2634675	*ZNF641*	427,943	0.001	49,168,798	A	0.029	−0.0058	0.0011	1.86E-07	−0.0060	0.0011	7.20E-08	7.37E-08	−0.0040	0.0010	5.40E-05
	16	rs438339	rs113956264	*RPL3L*	6,421	0.001	2,003,425	T	0.880	0.0035	0.0007	5.36E-08	0.0034	0.0007	1.60E-07	4.17E-07	0.0010	0.0006	8.60E-02
Tertiary signal	2	**rs807574**	rs807624	*DDX1*	24,768	0.055	15,807,239	A	0.600	0.0011	0.0004	1.45E-03	0.0019	0.0003	8.09E-08	7.62E-07	0.0019	0.0004	9.90E-08
	7	**rs13227214**	rs3757387	*IRF5*	164,269	0.057	128,740,355	C	0.460	−0.0024	0.0003	1.12E-12	−0.0018	0.0003	5.64E-08	2.40E-07	−0.0027	0.0003	6.60E-15
	9	rs7035892	rs2039424	*PIP5K1B*	107,868	0.089	71,540,042	A	0.840	0.0053	0.0006	1.27E-17	0.0037	0.0006	1.23E-09	2.43E-07	−0.0016	0.0008	6.20E-02
	11	**rs81205**	rs233438	*KCNQ1*	4,412	0.261	2,798,804	A	0.540	0.0034	0.0004	4.73E-20	0.0018	0.0003	1.28E-07	1.45E-06	0.0020	0.0004	3.50E-07
	11	rs294345	rs3925584	*DCDC1*	−93,675	0.012	30,666,660	T	0.067	−0.0057	0.0007	2.32E-14	−0.0035	0.0007	3.01E-07	4.21E-07	−0.0001	0.0008	8.50E-01
	11	**rs1193692**	rs11227260	*KAT5*	42,911	0.025	65,504,069	A	0.600	−0.0027	0.0005	4.33E-09	−0.0024	0.0005	2.86E-07	7.54E-07	−0.0020	0.0007	4.50E-03
	15	**rs4775830**	rs1153855	*GATM*	−127,414	0.167	45,533,344	A	0.430	−0.0017	0.0004	1.14E-06	0.0019	0.0003	4.65E-09	2.49E-07	0.0018	0.0004	2.10E-06
	20	rs75041355	rs6127099	*CYP24A1*	6,360	0.056	52,737,762	A	0.034	0.0083	0.0011	3.66E-14	0.0059	0.0011	4.87E-08	1.36E-06	0.0031	0.0012	8.90E-03
Signal of 4th	2	**rs2075251**	rs35472707	*LRP2*	15,877	0.016	170,011,458	A	0.750	−0.0028	0.0004	8.41E-12	−0.0021	0.0004	1.12E-07	1.76E-06	−0.0031	0.0004	2.80E-15
	6	**rs6912283**	rs881858	*LINC01512*	−442,115	0.000	43,364,494	A	0.560	−0.0009	0.0004	1.34E-02	−0.0018	0.0003	8.72E-08	5.50E-07	−0.0018	0.0004	4.20E-07
	9	**rs4745268**	rs2039424	*PIP5K1B*	−26,649	0.107	71,405,525	T	0.310	0.0035	0.0004	4.45E-19	0.0022	0.0004	1.00E-08	1.82E-06	0.0012	0.0004	6.00E-03
	11	**rs1056819**	rs233438	*KCNQ1*	155,469	0.002	2,949,861	T	0.200	−0.0021	0.0004	2.10E-06	−0.0023	0.0004	1.65E-07	5.51E-07	−0.0022	0.0004	9.50E-07
	20	**rs2585441**	rs6127099	*CYP24A1*	6,553	0.056	52,737,955	C	0.190	−0.0039	0.0005	6.57E-14	−0.0025	0.0005	1.45E-06	3.18E-06	−0.0026	0.0004	1.00E-08
	20	**rs6062357**	rs2261092	*ZGPAT*	538,806	0.001	62,892,739	T	0.450	0.0020	0.0004	2.19E-08	0.0019	0.0004	8.24E-08	5.50E-07	0.0013	0.0003	2.50E-04
Signal of 5th	6	**rs76426793**	rs12207180	*SLC22A2*	−66,715	0.005	160,566,392	A	0.120	−0.0022	0.0005	4.85E-05	−0.0023	0.0005	1.29E-06	1.73E-06	−0.0049	0.0006	2.50E-16
	7	**rs2695565**	rs2365286	*LINC01006*	139,133	0.000	156,397,312	A	0.200	0.0022	0.0004	6.54E-07	0.0023	0.0004	5.27E-07	9.67E-07	0.0027	0.0004	7.00E-10
	15	rs4886425	rs10851885	*NRG4*	−2,179,960	0.001	74,124,543	A	0.170	−0.0027	0.0005	4.26E-09	−0.0023	0.0005	4.58E-07	5.43E-07	−0.0003	0.0004	4.50E-01
Signal of 6th	7	**rs6951593**	rs2365286	*LINC01006*	12,546	0.110	156,270,725	A	0.047	0.0060	0.0010	4.65E-10	0.0042	0.0009	3.90E-06	7.69E-06	0.0038	0.0009	1.30E-05

This table contains the list of novel independent eGFR-associated signals identified by the quasi-adaptive method and replication results. Chr: chromosome; Signal: novel independent signal identified by quasi-adaptive method; Index: known index SNP in the corresponding locus has previously been reported in GWAS of eGFR; Closest gene: the closest gene to index SNP; D[bp]: the distance between index SNP and signal; r^2^: pairwise LD correlation between index SNP and signal using UKBB reference sample; Pos: position of signal; EA: effect allele of signal; EAF: frequency of the effect allele of signal; GWAS-MA: European-ancestry-specific GWAS meta-analysis; GCTA: approximate conditional analyses implemented in GCTA; Effect: effect of signal; Se: standard error of signal; P: *p*-value of signal; α-threshold: SNP-specific α-threshold assigned by quasi-adaptive method to a signal; Replication-UKBB: replication analysis by BOLT-linear mixed model in United Kingdom Biobank data set; Bold font indicates replicated independent signals*.*

### Replication of novel multiple independent signals in European-ancestry individuals

**FIGURE 1:**
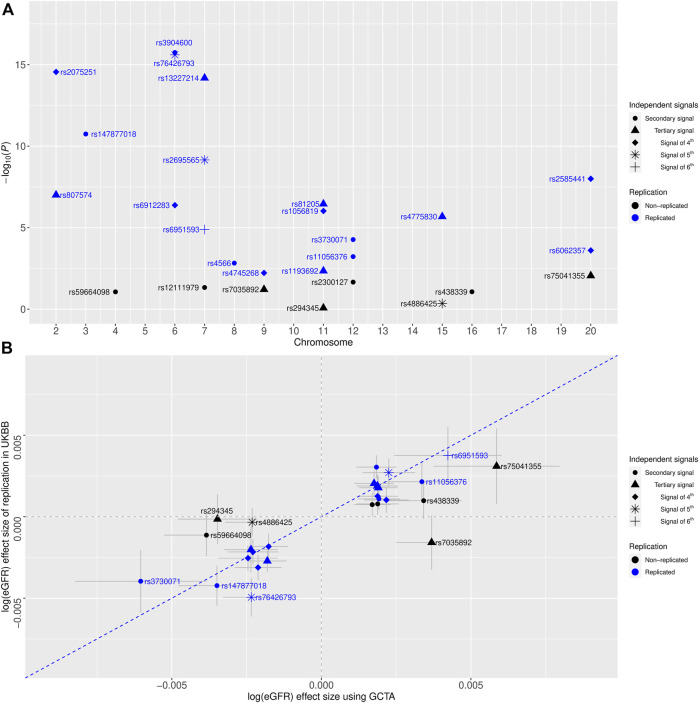
**(A)** Replication of eGFR-associated multiple independent signals identified by the quasi-adaptive method using the United Kingdom Biobank (UKBB) genotype data among European-ancestry individuals. The *x*-axis shows the chromosome number, and the *y*-axis is the ${-\log}_{10}\left(P\right)$ of the conditional GWAS of eGFR. Color coding reflects evidence of replication, which is coded as replicated (blue) and non-replicated (black). Different shapes showed multiple independent signals. **(B)**, comparing genetic effect estimates between conditional analysis using GCTA on the GWAS meta-analysis of a previous GWAS of eGFR (*x*-axis) and by conditional GWAS of eGFR on UKBB (*y*-axis). Color coding reflects replication evidence, coded as significant (blue) and non-significant (black). Error bars correspond to 95% confidence intervals. Pearson’s correlation coefficient *r* ^2^ = 0.937 (95% CI = 0.84, 0.98) for the replicated signals. The blue dashed line corresponds to the diagonal line.

To assess the validity of our newly identified independent signals, we conducted conditional eGFR-association analyses using individual-level genotype data among 408,608 European-ancestry participants of the UKBB as independent replication (Methods). For 27 novel independent signals, we conducted 27 conditional analyses (Supplementary Table S2). In total, replication was achieved for 19 signals (Five secondary signals, five tertiary signals, six signals of fourth, two signals of fifth, and one signal of sixth) after applying multiple testing corrections (Methods, Table 1 and Figure 1A). Of note, seven of these signals achieved genome-wide significant conditional *p*-values, and additional four signals were nominally significant (*p* < 0.05) in the replication analysis. Effect estimates for the replicated signals showed a strong correlation (*r* ^2^ = 0.937) with the discovery results (Figure 1B).

For better comparison, the regional association plots were generated for the unconditional associations and the conditional associations with the highlighted known index and the novel independent signal separately (Supplementary Figures S1-S57). Of note, the new independent signals rs3904600, rs13227214, rs81205, rs2075251, rs2695565, and rs6951593 (identified by the quasi-adaptive method based on the meta-analysis of the previous GWAS of eGFR (Wuttke et al., 2019)) showed smaller *p*-values in their unconditional analysis within the UKBB compared to their corresponding index SNP (Supplementary Figures S4, S19, S22, S31, S52, S55).

### Colocalization with gene expression

Colocalization analyses were performed with eQTLs in *cis* across 51 tissues, including kidney cortex, glomerular, and tubulointerstitial for the 17 known eGFR-associated index SNPs as well as for the 19 new independent signals using unconditional and conditional eGFR results (Methods and Supplementary Table S2).

**FIGURE 2:**
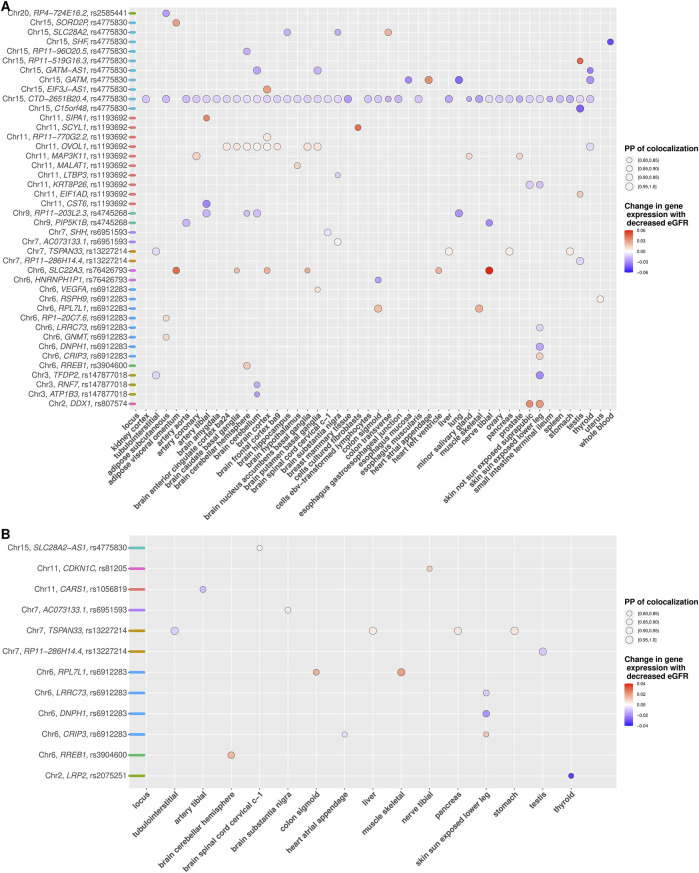
**(A,B)** Colocalization of eGFR association of novel independent signals with gene expression (cis eQTLs) across tissues. **(A** and **B)** depict colocalization results based on unconditional and conditional eGFR association analyses, respectively. Gene with at least one posterior probability of colocalization (PP ≥ 80%) across tissues (*x*-axis) is shown with the respective underlying variant and chromosome number (*y*-axis). Colocalizations are illustrated as dots, where dot size corresponds to the PP and are colored according to the predicted change in gene expression relative to the lower eGFR. Color coding on the *y*-axis reflects the locus.

Using unconditional eGFR associations, we identified 56 genes mapping to 13 out of 17 index SNPs for which *cis*-eQTL in at least one tissue colocalized with an eGFR-associated signal with a high PP ( ≥80% ) (Supplementary Table S8 and Supplementary Figure S58). Results for the 19 new independent signals using unconditional GWAS associations revealed significant colocalization in at least one tissue for 42 genes mapping to 11 of the 19 independent signals (Supplementary Table S8 and Figure 2A).

To determine more robust evidence of colocalization, we re-run the colocalization for each known index SNP using the corresponding conditional eGFR association. We identified 53 genes mapping to 11 index SNPs for which *cis*-eQTL in at least one tissue colocalized with an eGFR-associated signal with a high PP (Supplementary Table S9 and Supplementary Figure S59). We identified 10 genes that colocalized with four index SNPs exclusively using conditional associations, which would have remained undetected if only colocalization of unconditional associations had been considered (Table 2). Comparing colocalization for index SNPs based on unconditional with conditional associations across all tissues revealed consistent results for 45 genes mapping to eight index SNPs (Supplementary Table S10), which means that multiple independent signals did not affect the colocalization analyses at these loci. On the other hand, 11 genes mapping to six index SNPs were detected only by colocalization using unconditional association, indicating that multiple independent signals at these loci affected the colocalization analyses for the corresponding index SNPs (Supplementary Table S11).

**TABLE 2:** Summary of colocalization of eGFR association known index SNPs and novel independent signals with posterior probability (PP ≥ 80%). (A-B) contain summary of colocalization of eGFR association known index SNPs and novel independent signals with a high posterior probability of colocalization (PP) ≥ 80% in at least one tissue.

Rsid	Known	Chr	Gene	Tissue	Supplementary Figure
A: New colocalizations in kidney tissues with consistent results between conditional and unconditional association analyses					
** **rs1397764	Yes	3	TFDP2	tubulointerstitial	Supplementary Figure S62A
** **rs13227214	No	7	TSPAN33	tubulointerstitial	Supplementary Figure S76B
** **rs1153855	Yes	15	CTD-2651B20.4	kidney cortex	Supplementary Figure S74A
B: Summary of colocalization results identified exclusively by colocalization based on conditional association analyses, across all tissues					
rs35472707	Yes	2	KLHL41	brain spinal cord cervical c-1	Supplementary Figure S60A
rs2075251	No	2	LRP2	thyroid	Supplementary Figure S60B
rs12207180	Yes	6	RP11−288H12.3	small intestine terminal ileum	Supplementary Figure S63A
rs12207180	Yes	6	SLC22A2	esophagus gastroesophageal junction	Supplementary Figure S63A
rs12207180	Yes	6	SLC22A2	esophagus muscularis	Supplementary Figure S63A
rs12207180	Yes	6	SLC22A2	prostate	Supplementary Figure S63A
rs12207180	yes	6	SLC22A2	testis	Supplementary Figure S63A
rs12207180	yes	6	SLC22A3	artery tibial	Supplementary Figure S63A
rs6912283	no	6	CRIP3	heart atrial appendage	Supplementary Figure S65B
rs10086569	yes	8	RMDN1	adrenal gland	Supplementary Figure S68A
rs10086569	yes	8	WWP1	muscle skeletal	Supplementary Figure S68A
rs1056819	no	11	CARS1	artery tibial	Supplementary Figure S71B
rs81205	no	11	CDKN1C	nerve tibial	Supplementary Figure S72B
rs4775830	no	15	SLC28A2-AS1	brain spinal cord cervical c-1	Supplementary Figure S74B
rs2261092	yes	20	EEF1A2	whole blood	Supplementary Figure S75A
rs2261092	yes	20	MYT1	brain substantia nigra	Supplementary Figure S75A
rs2261092	yes	20	SLC17A9	brain substantia nigra	Supplementary Figure S75A
rs2261092	yes	20	ZGPAT	ovary	Supplementary Figure S75A

Rsid: SNP rsid; Known: SNP was reported as an index SNP in the previous report of eGFR from Wuttke et al. (2019) is labeled as “yes”, and novel independent signals identified by quasi-adaptive method are labeled as “no”; Chr: chromosome; Supplementary Figure: comparison of the colocalization results for known index SNPs and novel independent signals using conditional *versus* unconditional associations.

Colocalization for each new independent signal using conditional association analysis mapped 12 genes to eight of the 19 independent signals with colocalization PP ≥ 80% in at least one tissue (Supplementary Table S9 and Figure 2B). We identified eight genes mapping to 4 novel independent signals with consistent results between colocalization based on unconditional and conditional associations, indicating accurate colocalization results for novel independent signals at these loci (Supplementary Table S10). In addition, five genes mapping to 5 novel independent signals were identified exclusively by colocalization using conditional associations, which would have remained undetected if only colocalization using unconditional associations had been considered (Table 2 and Figure 2B). On the other hand, 34 genes mapping to 9 novel independent signals were detected only by colocalization using unconditional associations, indicating that colocalization using unconditional association has less power to detect accurate results at these loci (Supplementary Table S11).

The complete comparison of the colocalization results for known index SNPs and novel independent signals using conditional *versus* unconditional associations are provided in Supplementary Figures S60-S76.

## Discussion

Application of our recently developed quasi-adaptive method to the publicly available GWAS meta-analysis results of eGFR among 783,978 European-ancestry individuals (Wuttke et al., 2019) and subsequent replication in additional 408,608 individuals from UKBB identified 19 novel independent eGFR association signals. These signals included five secondary signals, five tertiary signals, six signals of fourth, two signals of fifth, and one signal of sixth. These results would have gone undetected by conditional analysis applying the commonly used but too conservative genome-wide significance level of 5 × 10^–8^. Of note, the individuals included in the LD reference sample were also part of the replication stage, but an influence of the results is very unlikely because of the substantially larger sample size in the replication analysis, and the different methods applied (summary statistics with LD reference vs individual level conditional analysis).

Some previous reports on eGFR support our findings. For instance, our secondary signal rs147877018 was previously discovered as an eGFR-associated signal through conditional analysis implemented in GCTA (at locus-wide significance, *p* < 10^–5^)^20^ *.* In addition, Wuttke et al. (2019) (Wuttke et al., 2019) reported *ADCY6* as a novel eGFR candidate gene in humans by performing a nested candidate gene analysis in mice. *ADCY6* has not been reported to contain genome-wide significant eGFR-associated SNPs or to be located near known loci. However, in our study, the secondary signal rs3730071 was discovered near *ADCY6* (Supplementary Figure S13).

Colocalization of eGFR-associated known index SNPs and novel independent signals and gene expression implicate specific potential functional genes for follow-up. We investigated the kidney by using *cis*-eQTL dataset from the publicly available GTEx project (Aguet et al., 2019). However, the human kidney tissues have been poorly covered by the GTEx study, and only the kidney cortex with small sample size is included in this dataset. To overcome this limitation, we also investigated kidney tissue by using a *cis*-eQTL dataset from microdissected human glomerular and tubulointerstitial kidney portions from 187 individuals from the NEPTUNE study (Gillies et al., 2018).

The presence of multiple independent GWAS signals at a locus violates the assumption required by the applied colocalization method (one causal variant for each locus) and likely reduces the power to detect accurate colocalization results. In this context, Wu. et al. (2019) (Wu et al., 2019) showed that for a locus with multiple GWAS signals and/or multiple eQTL signals for the same gene, integration of conditional GWAS association and conditional eQTL led to more robust evidence of colocalization. Our project provides conditional eGFR association tests conducted in the UKBB individual-level genotype dataset. These tests were used to improve the colocalization analyses of the known index SNPs and novel independent signals to identify plausible effector genes related to eGFR. Our findings could be improved by adding the conditional eQTLs data, which may have affected our ability to colocalize signals. It is worth noting that the conditional eQTLs data are not available in our study.

The consistent results between colocalization using unconditional and conditional associations at a locus with multiple independent signals confirm that the colocalization based on unconditional association has enough power to detect accurate colocalization. On the other hand, inconsistent results indicate that colocalization based on unconditional association is affected by the presence of other independent signals at a locus and has less power to detect true colocalization. Therefore, we suggest more accurate results based on colocalization analyses using conditional association and eQTLs, revealing the plausible candidate genes after eliminating the potential effect of other multiple signals.

For instance, in tubulointerstitial and kidney cortex we revealed the known index SNPs rs1397764 and rs1153855 as the shared underlying variants for colocalization of lower eGFR with lower expression of *TFDP2* and *CTD−2651B20.4*, respectively. This was identified by colocalization based on both unconditional and conditional association analyses (Table 2A and Supplementary Figures S58, S59). Across other tissues, we suggest *SLC22A2* as a plausible candidate gene colocalized with index SNP rs12207180, which was detected only after eliminating the effect of other multiple signals at the locus (Table 2B and Supplementary Figure S59). *TFDP2*, *CTD−2651B20.4,* and *SLC22A2* were exclusively identified by our colocalization and have not been reported in the previous report of eGFR (Wuttke et al., 2019). *TFDP2* encodes E2F dimerization partner (DP)-2, which forms heterodimers with the E2F transcription factors resulting in transcriptional activation of cell cycle-regulated genes. Although the role of *TFDP2* in the context of renal disease has not been reported, several genetic associations in or near *TFDP2* have been reported in previous GWAS of eGFR and CKD (Kottgen et al., 2010; Pattaro et al., 2016; Hellwege et al., 2019; Morris et al., 2019; Wuttke et al., 2019). In addition, *TFDP2* was identified as a prioritized gene for eGFR by performing a transcriptome-wide association study (TWAS) and a summary Mendelian randomization test (Doke et al., 2021). Furthermore, the expression of *TFDP2* was associated with the eGFR index variant, specifically in kidney-specific eQTL associations (Graham et al., 2019). *CTD−2651B20.4* is a protein-kinase, interferon-inducible double-stranded RNA-dependent inhibitor, and repressor of (P58 repressor) (PRKRIR) pseudogene with Ensembl version identifier ENSG00000259433.2. There is no explicit function for *CTD−2651B20.4*, and it has not been reported to contain or be located near associated variants with phenotypes, diseases, and traits in humans or other species. *SLC22A2* is specifically expressed in the kidney and plays a critical role in the renal secretion of various cationic compounds (Aoki et al., 2008)*. SLC22A2* encodes the polyspecific organic cation transporter (OCT2) and mediates tubular uptake of organic compounds including creatinine in the basolateral membrane of renal tubular epithelial cells (Urakami et al., 2004)*. SLC22A2* has been reported to contain or to be located near genetic associations in multiple GWAS of eGFR and CKD (Kottgen et al., 2010; Mahajan et al., 2016; Morris et al., 2019; Wuttke et al., 2019).

Our colocalization of novel independent signals suggests rs13227214 as the shared underlying variant for colocalization of lower eGFR with lower expression of *TSPAN33*in tubulointerstitial tissue, which was robustly identified based on both unconditional and conditional association analyses (Table 2A and Figure 2). Furthermore, in thyroid and nerve tibial tissue, we suggest *LRP2* and *CDKN1C* as the plausible candidate genes colocalized with rs2075251 and rs81205, respectively, which were detected only by colocalization based on conditional associations (Table 2B and Figure 2B). *TSPAN33*, *LRP2*, and *CDKN1C* were identified exclusively by our colocalization of novel independent signals and would have remained undetected if only colocalization of the corresponding index SNPs rs3757387, rs35472707, and rs233438 were considered at these loci (Supplementary Figure S67, Supplementary Figure S60, and Supplementary Figure S72). *TSPAN33* is a member of the tetraspanin family and encodes a transmembrane protein. *TSPAN33* is highly expressed in the kidney and *TSPAN33* mRNA is detectable in the kidney by both microarray and qPCR (Luu et al., 2013). Furthermore, in colocalization analysis of kidney-specific eQTL association (kidney cortex (Ko et al., 2017), glomerulus, and tubule-interstitial compartments (Gillies et al., 2018), *TPSAN33* showed significant colocalization with the eGFR association (Graham et al., 2019). *LRP2* encodes the megalin receptor (Nielsen and Christensen, 2010) and connected to its seed gene *DAB2*, through protein–protein interaction (Hosaka et al., 2009). Chasman et al. (2012) identified *LRP2* related to the kidney function through connection with the previously known eGFR gene *DAB2* and prior biological knowledge about megalin system in kidney function (Chasman et al., 2012). *CDKN1C* expressed in the heart, brain, lung, skeletal muscle, kidney, pancreas and testis. Up-regulation of miR-199a-5p through suppressing *CDKN1C* might promote cell proliferation in autosomal dominant polycystic kidney disease tissues (Sun et al., 2015), which is a genetic disorder characterized by the growth of numerous cysts in the kidney often causes renal failure with many serious complications.

In summary, we have extended our quasi-adaptive method toward identifying multiple independent SNPs within a locus, applied this method to an eGFR meta-analysis result, and discovered and replicated novel eGFR-associated SNPs. Using these results, we revealed plausible candidate genes for eGFR by colocalization, partly undetected using standard approaches. These findings will help improve the understanding of biological mechanisms underlying kidney function and may subsequently help reducing the burden of CKD.

## Data Availability

The datasets presented in this study can be found in online repositories. The names of the repository/repositories and accession number(s) can be found in the article and Supplementary Material.
