# Supplementary material for: Discovery of novel eGFR-associated multiple independent signals using a quasi-adaptive method
Source: Front Genet. 2022 Oct 31;13:997302. doi: 10.3389/fgene.2022.997302 (PMC9660290; doi:10.3389/fgene.2022.997302)

SUPPLEMENTARY INFORMATION

Discovery of novel eGFR-associated multiple independent signals using a quasi-adaptive method

Sahar Ghasemi *et al.*

Content description:

Figures 1-57: Represent regional association plots for loci with replicated novel independent signals identified by the quasi-adaptive method. Three plots were shown for each locus. The **first plot** shows a regional association plot based on eGFR association analysis performed on UKBB individual-level genotype data among European-ancestry participants with highlighted novel independent signal and known index SNP, respectively, as an LD reference variant (colored purple). The **second plot** is a regional association plot based on conditional eGFR association analysis conditioned on known index SNP performed on UKBB individual-level genotype data among European-ancestry participants with the novel independent signal as LD reference variant (colored purple). The **third plot** is a regional association plot based on conditional eGFR association analysis conditioned on a novel independent signal performed on UKBB individual-level genotype data among European-ancestry participants with known index SNP as LD reference variant (colored purple). The details of the conditional eGFR association analyses were explained for each plot separately.

Figures 58-59: Colocalization of eGFR-associated known index SNPs with gene expression (*cis* eQTLs) across all tissues. **Figure 58** and **Figure 59** depict colocalization results based on unconditional and conditional eGFR association analyses, respectively. Genes with at least one posterior probability of colocalization (PP ≥ 80%) across tissues (x axis) are shown with the respective underlying variants and chromosome numbers (y axis). Colocalizations are illustrated as dots, where dot size corresponds to the PP and are colored according to the predicted change in gene expression relative to the lower eGFR. Color coding on y axis reflects locus.

Figures 60-76: Represent colocalization results for known index SNP and novel independent signal at each locus across all tissues. **Plot A** and **Plot B** show posterior probability of colocalization (PP of one common variant) based on eGFR association analysis (y axis) and conditional eGFR association analysis (x axis) for known index SNP and novel independent signal, respectively. The gray dash line represents PP = 80%, with high evidence for colocalization (PP > 80%) shown in color and low evidence (PP < 80%) shown in gray. The blue dashed line corresponds to the diagonal line. The details of the conditional eGFR association analyses were explained for each plot separately.

Figure 1: Regional association plot for index SNP rs1397764 (3:141750810_A/G) and secondary signal rs147877018 (3:141813349_G/A) using eGFR association analysis.

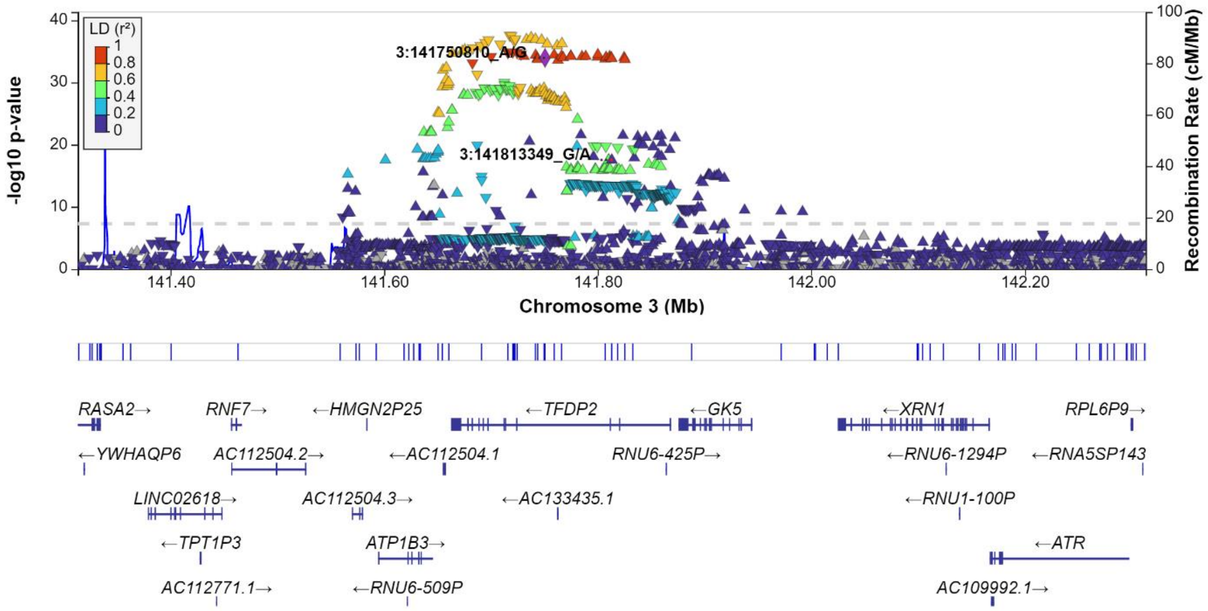

Figure 2: Regional association plot for secondary signal rs147877018 (3:141813349_G/A) using conditional eGFR association analysis (conditioned on index SNP).

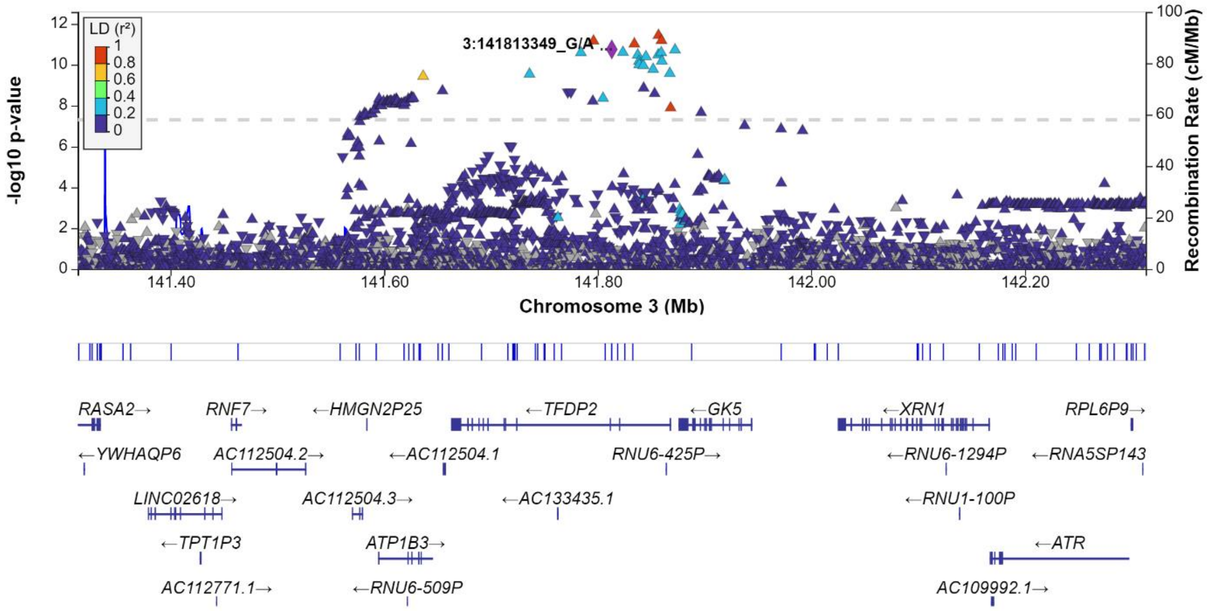

Figure 3: Regional association plot for index SNP rs1397764 (3:141750810_A/G) using conditional eGFR association analysis (conditioned on secondary signal).

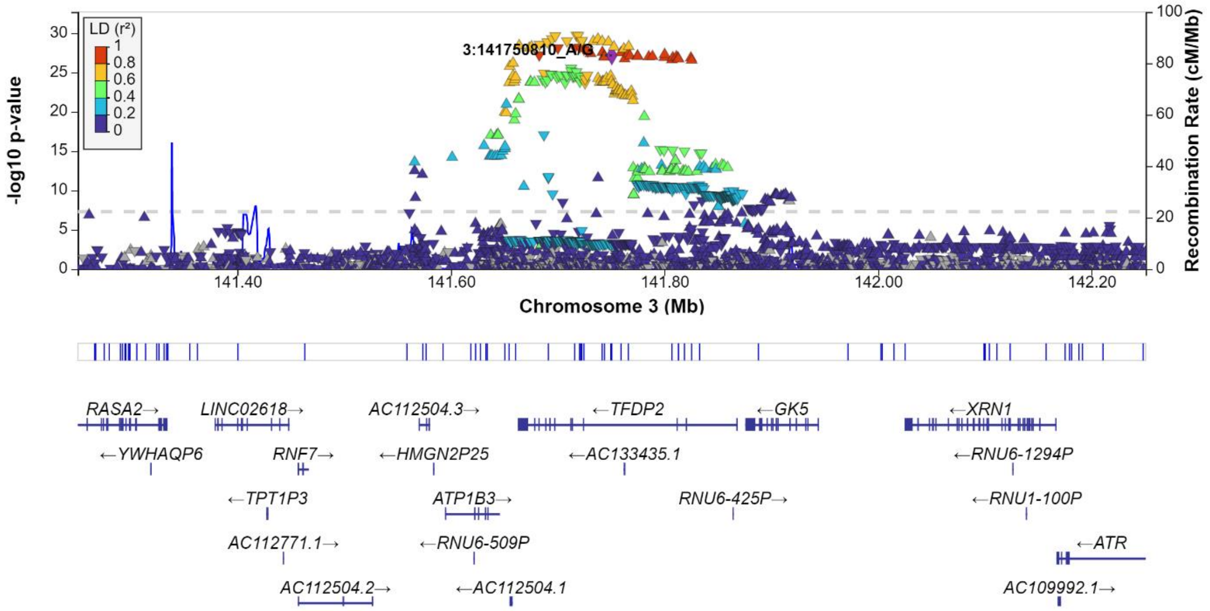

Figure 4: Regional association plot for index SNP rs6921580 (6: 7203714_C/G) and secondary signal rs3904600 (6: 7109665_C/G) using eGFR association analysis.

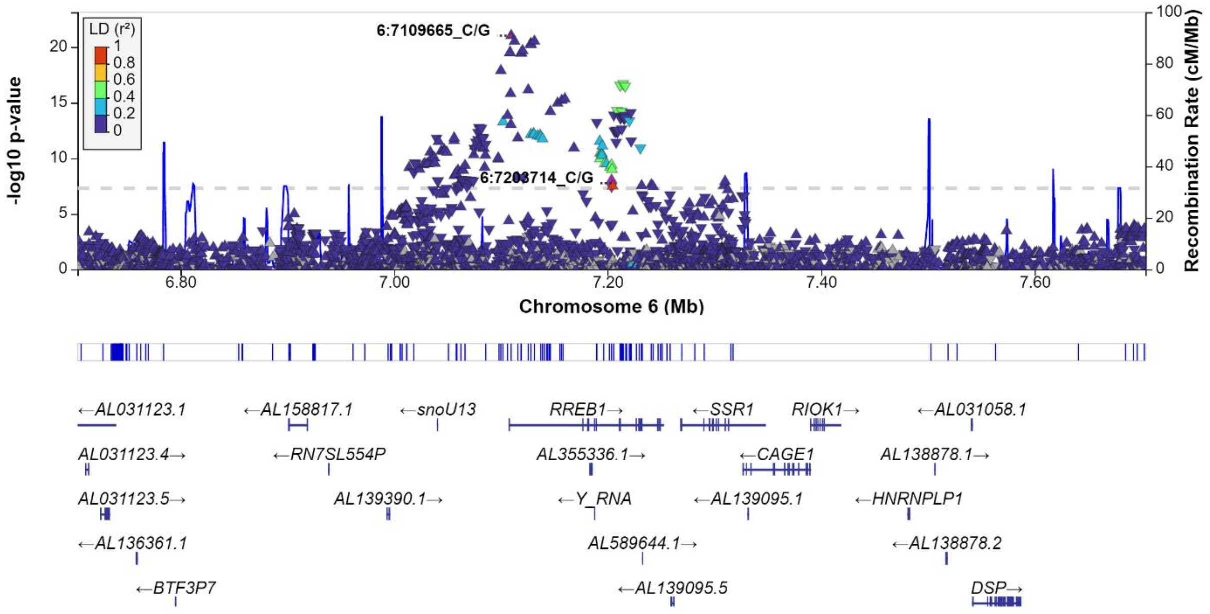

Figure 5: Regional association plot for secondary signal rs3904600 (6: 7109665_C/G) using conditional eGFR association analysis (conditioned on index SNP).

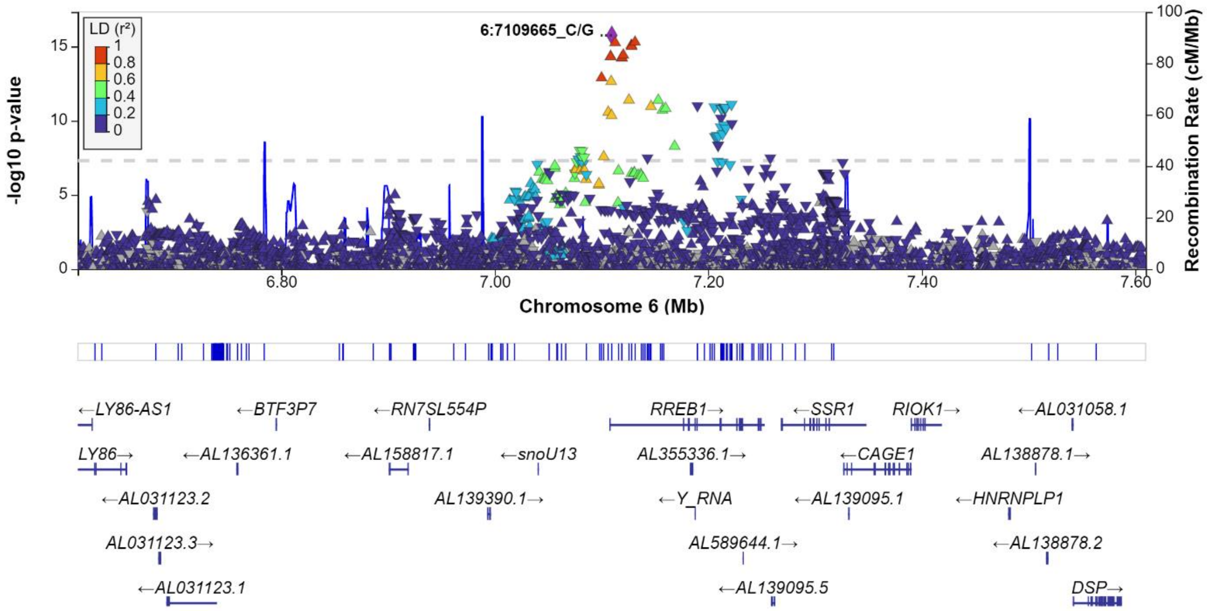

Figure 6: Regional association plot for index SNP rs6921580 (6: 7203714_C/G) using conditional eGFR association analysis (conditioned on secondary signal).

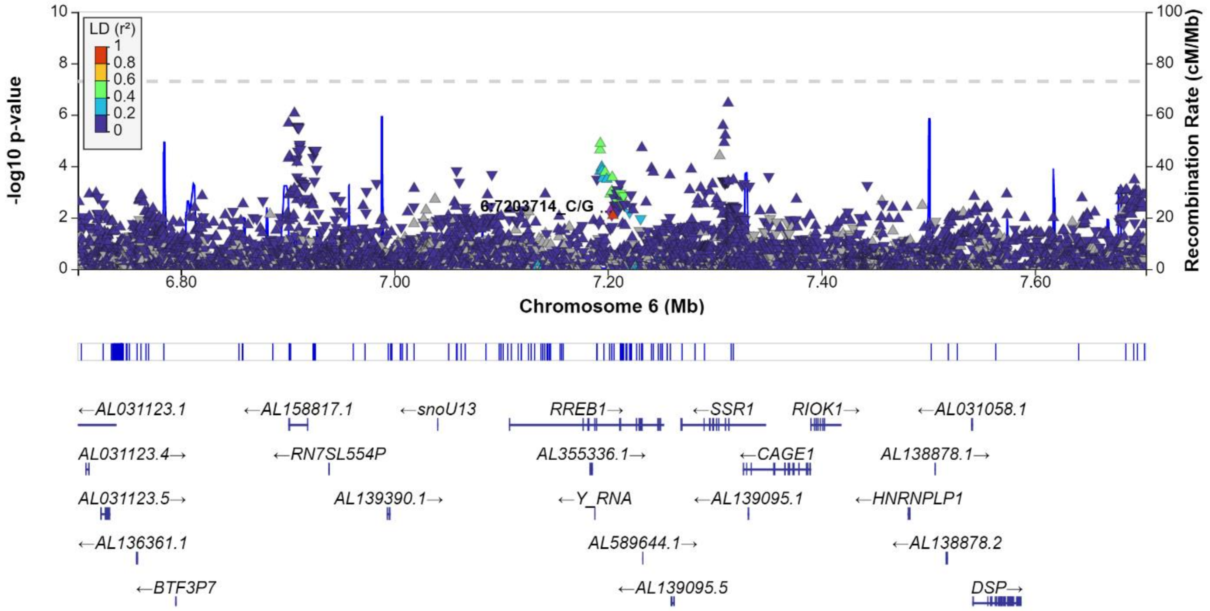

Figure 7: Regional association plot for index SNP rs10086569 (8: 87247209_C/T) and secondary signal rs4566 (8: 86361082_G/T) using eGFR association analysis.

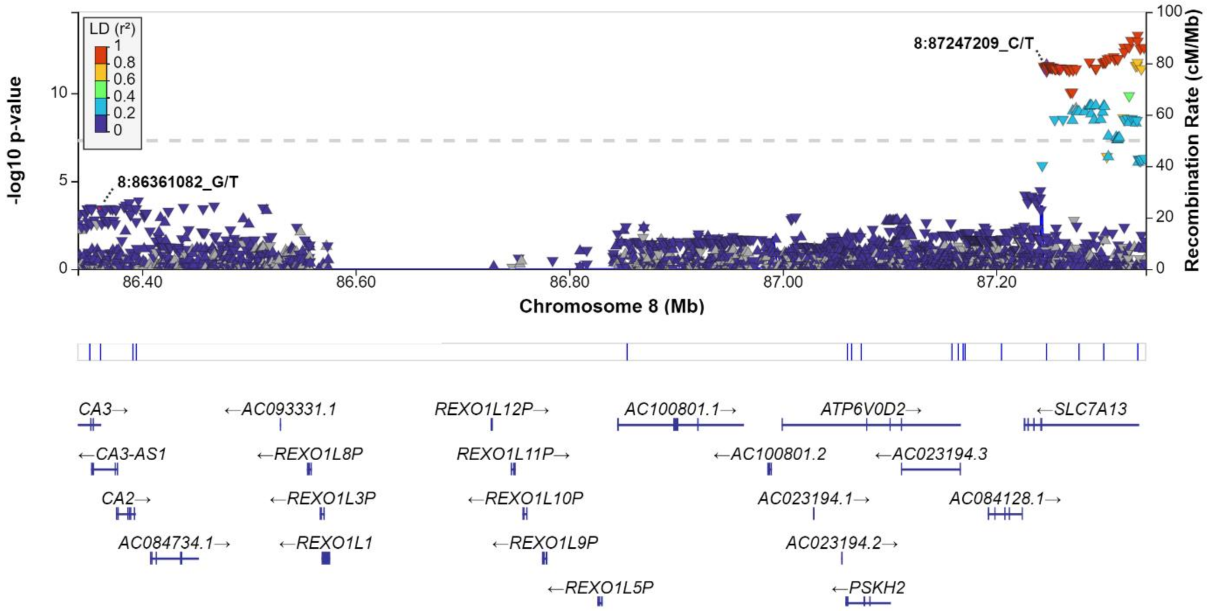

Figure 8: Regional association plot for secondary signal rs4566 (8: 86361082_G/T) using conditional eGFR association analysis (conditioned on index SNP).

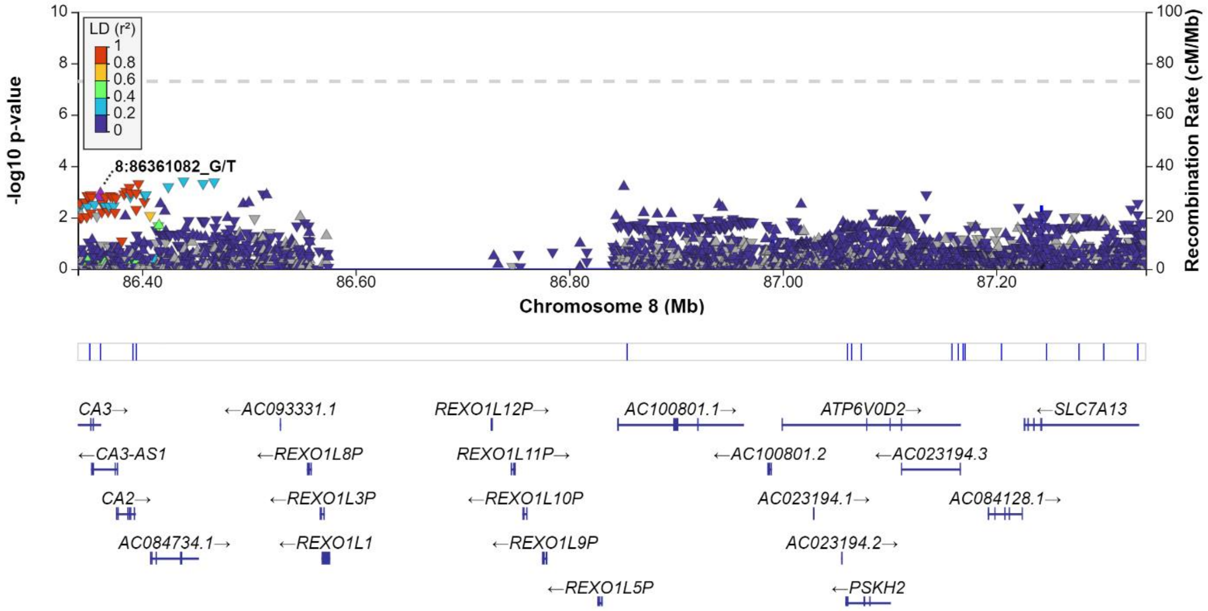

Figure 9: Regional association plot for index SNP rs10086569 (8: 87247209_C/T) using conditional eGFR association analysis (conditioned on secondary signal).

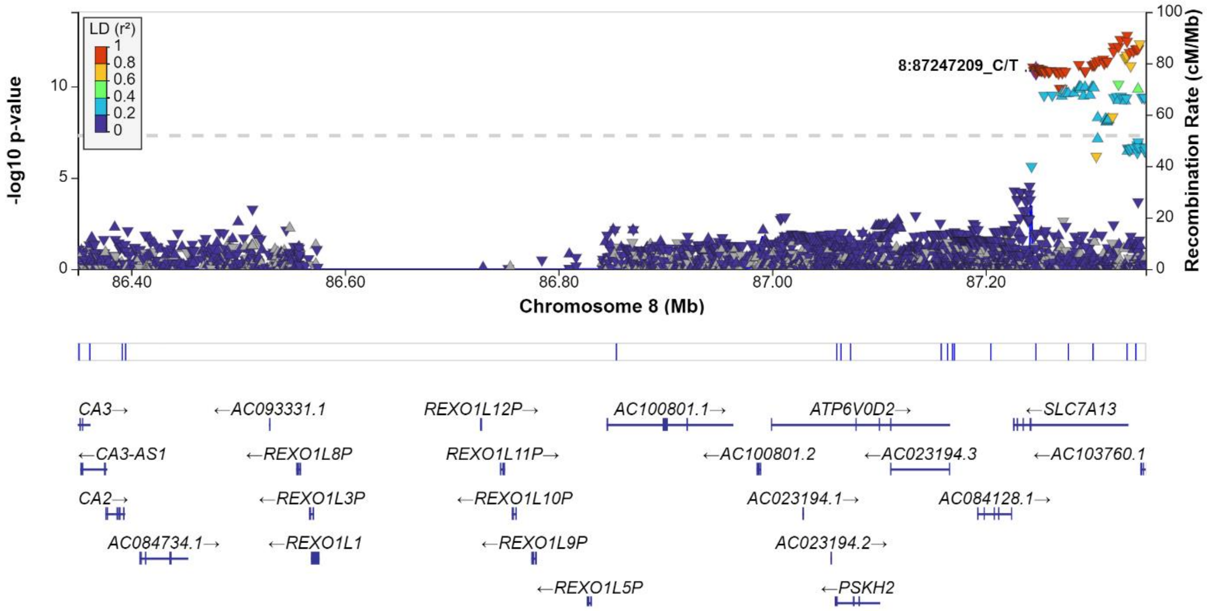

Figure 10: Regional association plot for index SNP rs10846157 (12:15325031_A/C) and secondary signal rs11056376 (12:15307394_A/G) using eGFR association analysis.

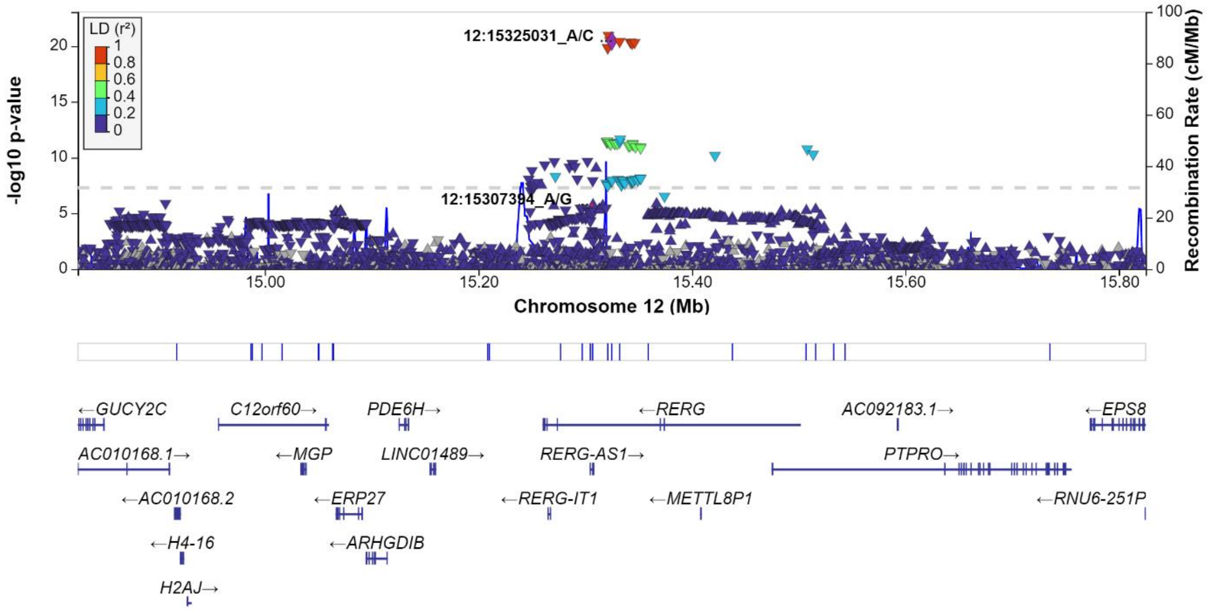

Figure 11: Regional association plot for secondary signal rs11056376 (12:15307394_A/G) using conditional eGFR association analysis (conditioned on index SNP).

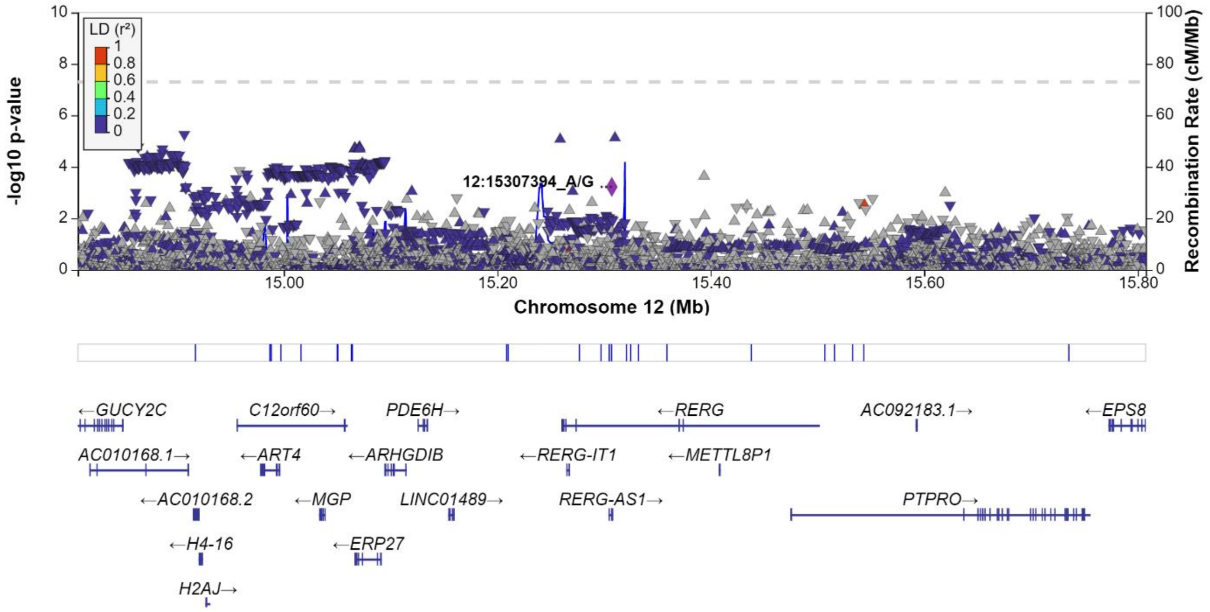

Figure 12: Regional association plot for index SNP rs10846157 (12:15325031_A/C) (conditioned on secondary signal) using conditional eGFR association analysis.

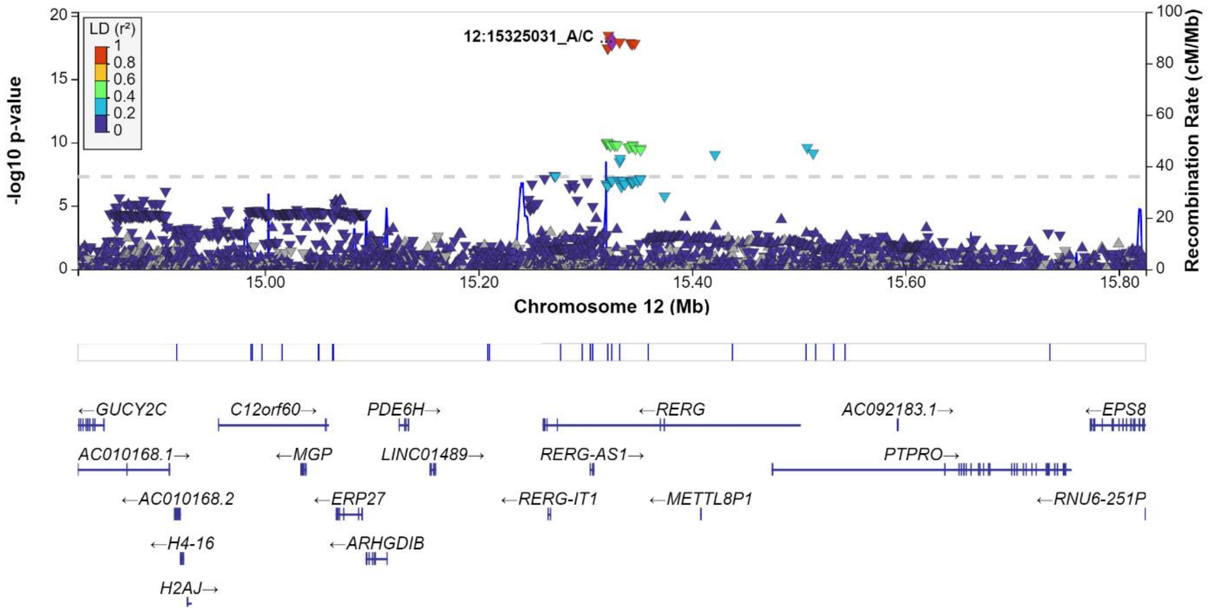

Figure 13: Regional association plot for index SNP rs2634675 (12: 48740855_A/G) and secondary signal rs3730071 (12: 49168798_C/A) using eGFR association analysis.

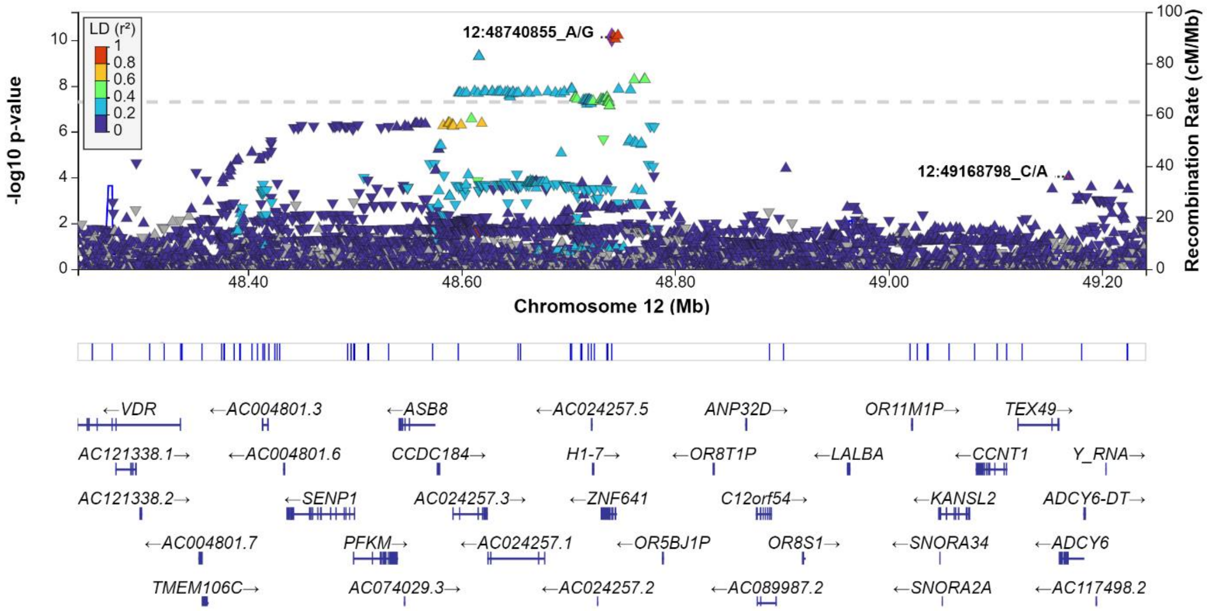

Figure 14: Regional association plot for secondary signal rs3730071 (12: 49168798_C/A) using conditional eGFR association analysis (conditioned on index SNP)

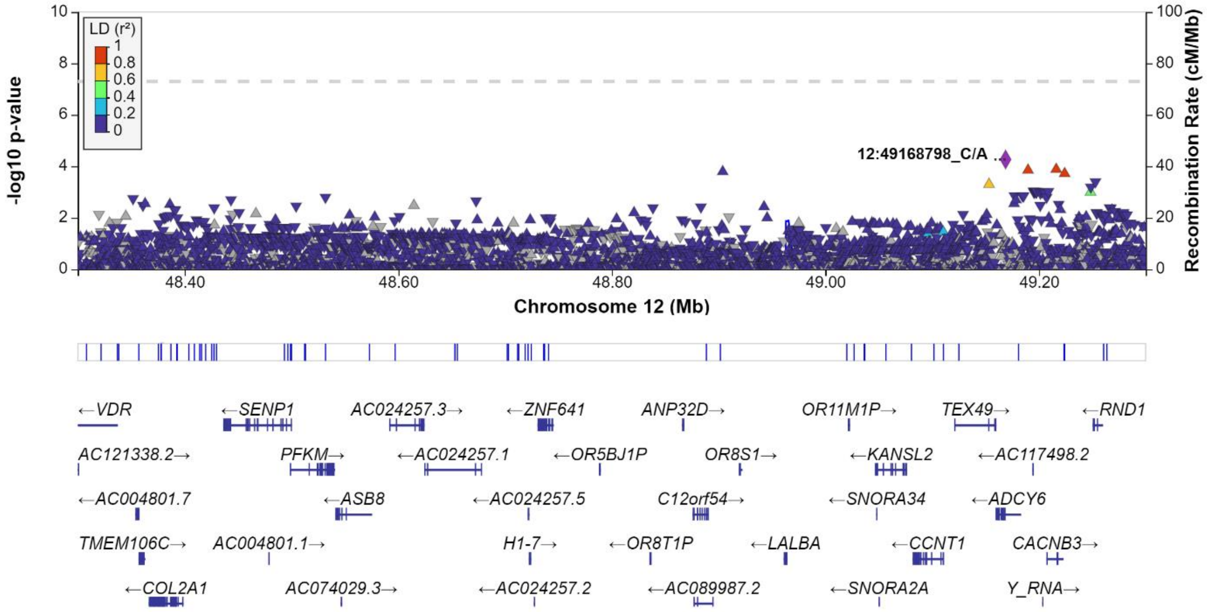

Figure 15: Regional association plot for index SNP rs2634675 (12: 48740855_A/G) using conditional eGFR association analysis (conditioned on secondary signal).

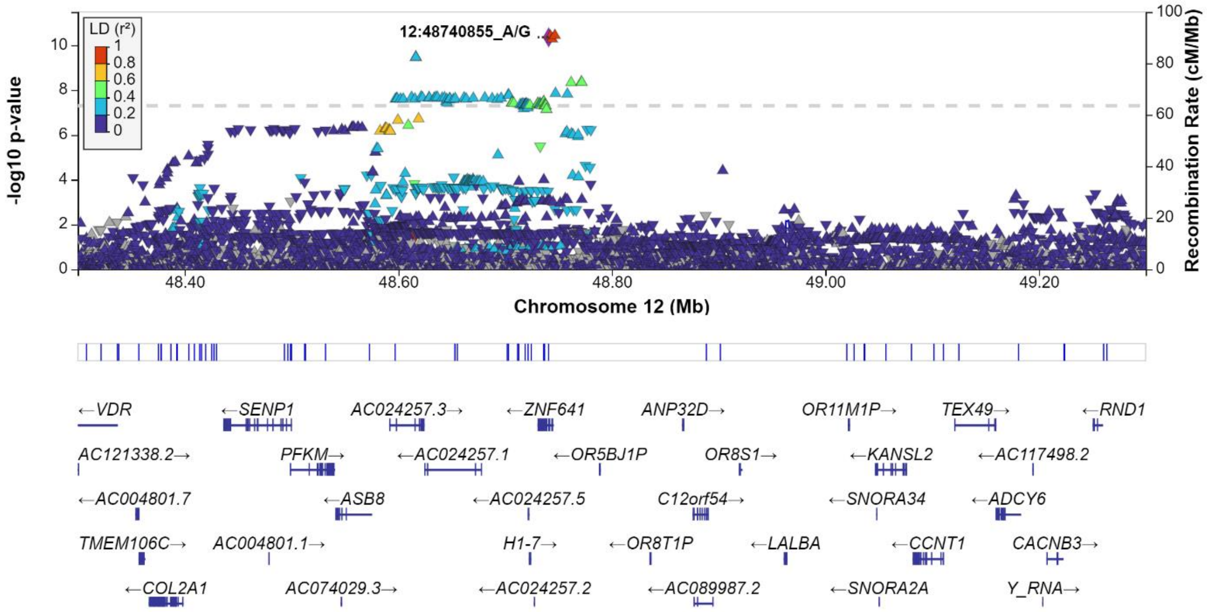

Figure 16: Regional association plot for index SNP rs807624 (2:15782471_G/T) and tertiary signal rs807574 (2:15807239_A/G) using eGFR association analysis.

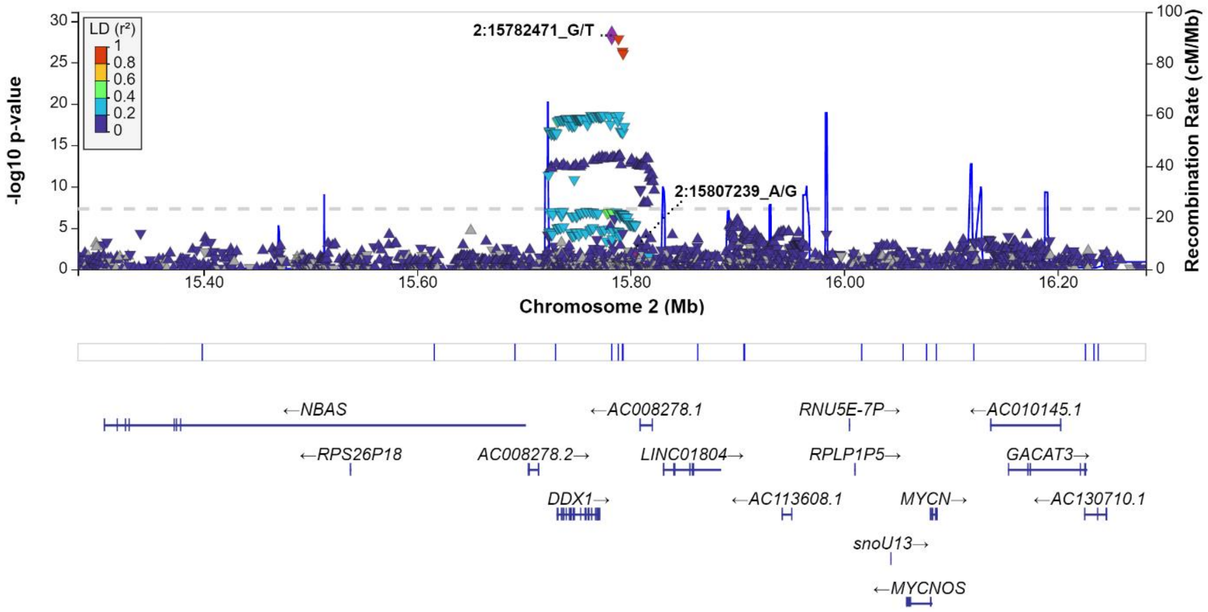

Figure 17: Regional association plot for tertiary signal rs807574 (2:15807239_A/G) using conditional eGFR association analysis (conditioned on index SNP and secondary signal).

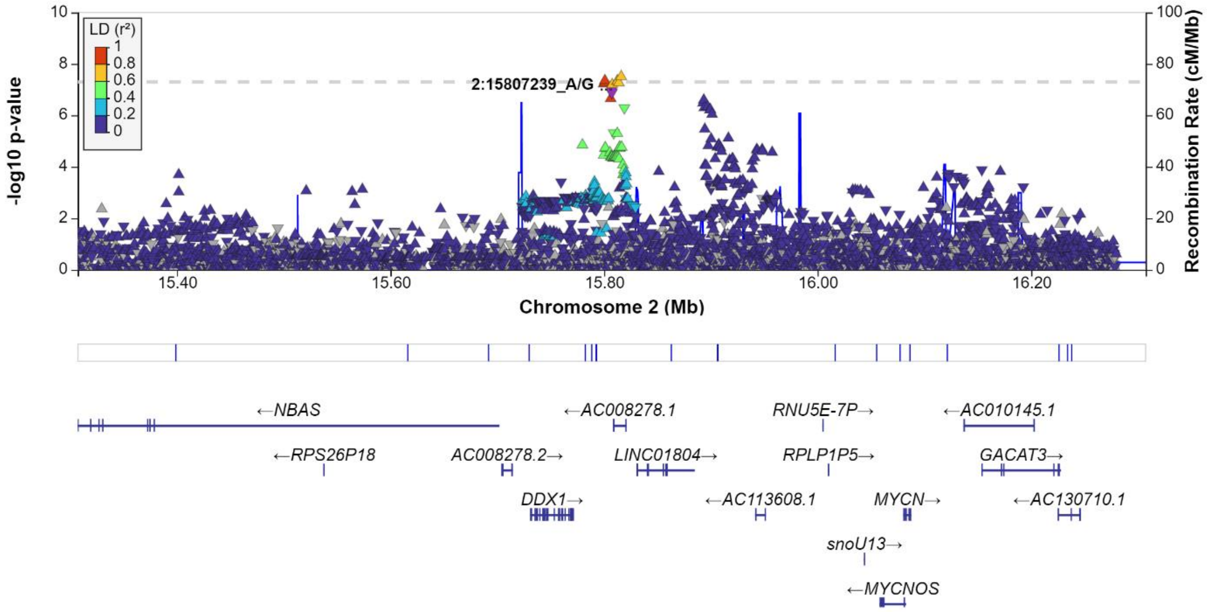

Figure 18: Regional association plot for index SNP rs807624 (2:15782471_G/T) using conditional eGFR association analysis (conditioned on secondary signal and tertiary signal).

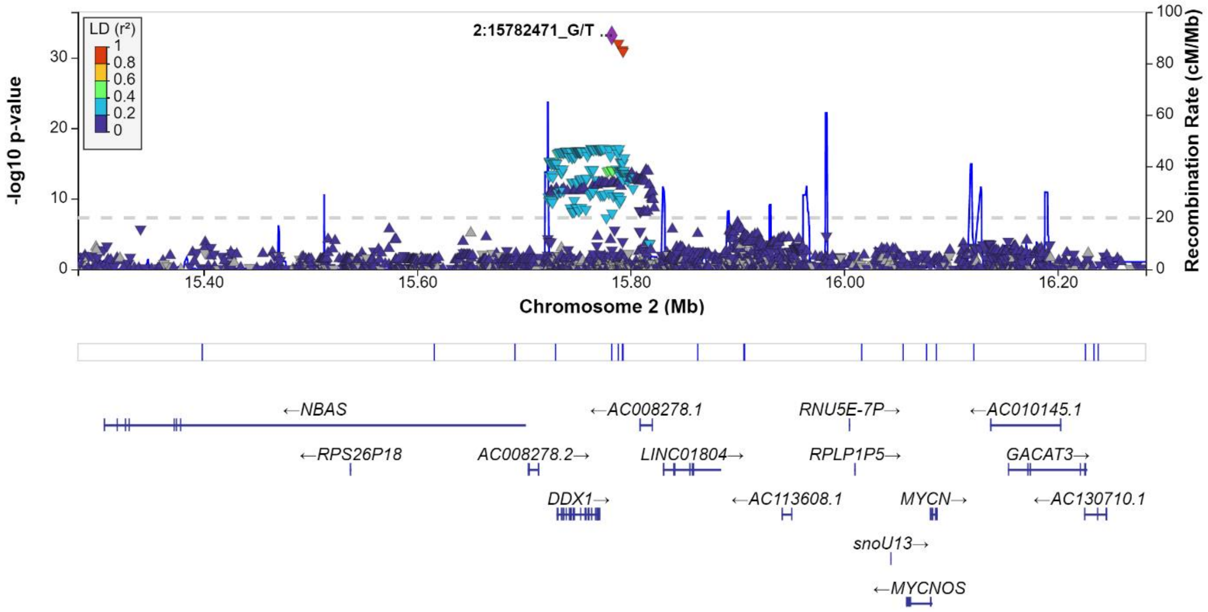

Figure 19: Regional association plot for index SNP rs3757387 (7:128576086_T/C) and tertiary signal rs13227214 (7:128740355_C/G) using eGFR association analysis.

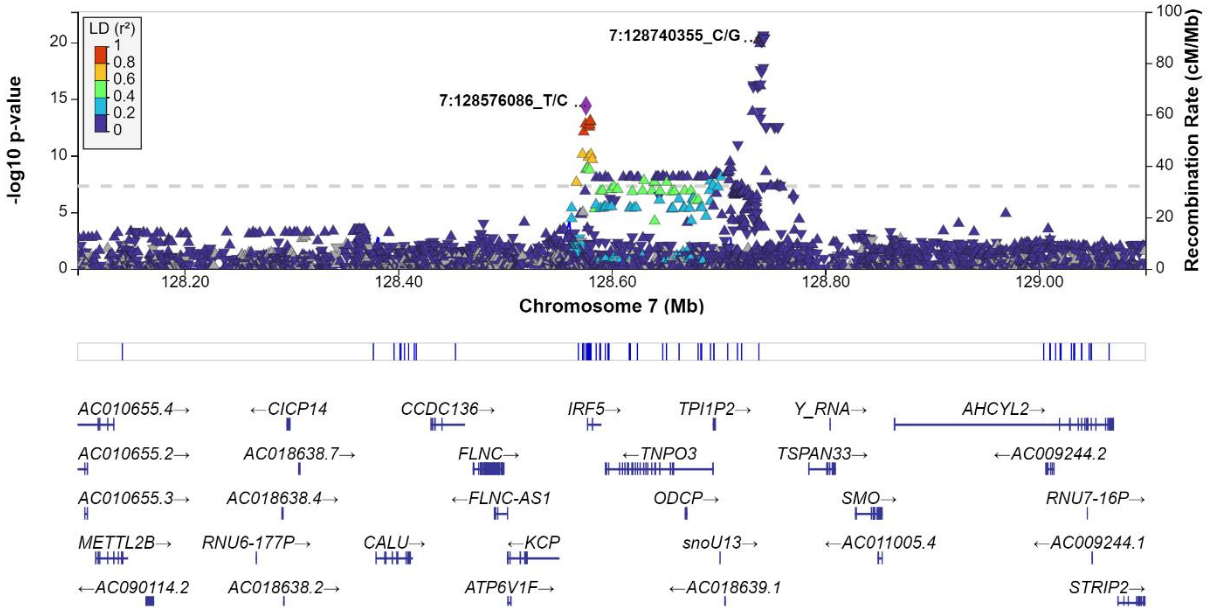

Figure 20: Regional association plot for tertiary signal rs13227214 (7:128740355_C/G) using conditional eGFR association analysis (conditioned on index SNP and secondary signal).

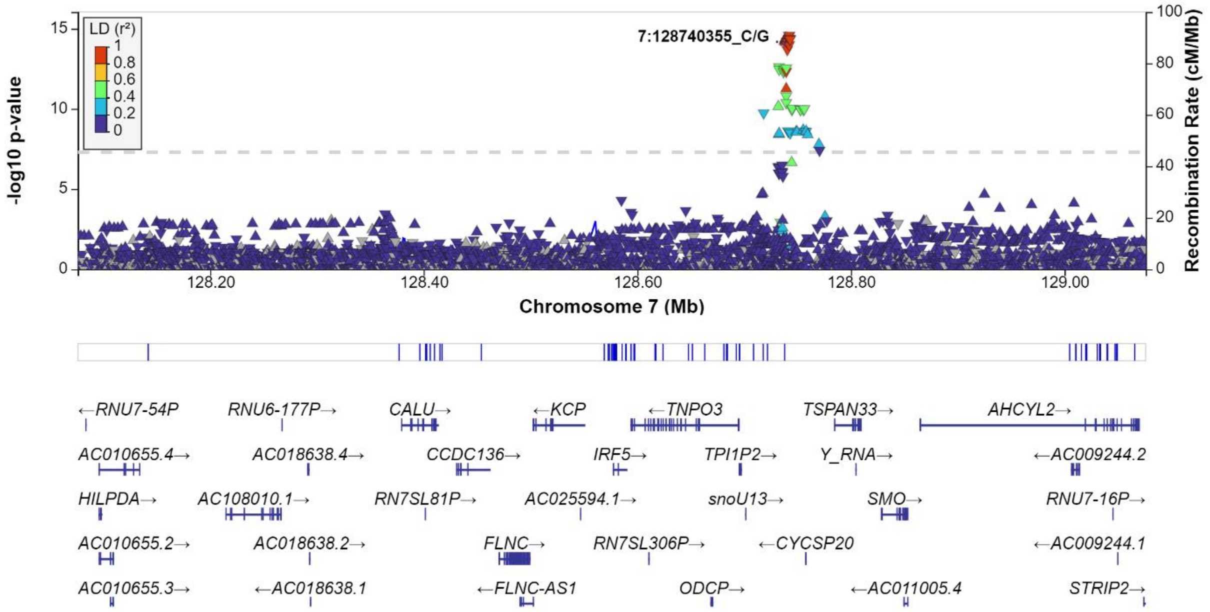

Figure 21: Regional association plot for index SNP rs3757387 (7:128576086_T/C) using conditional eGFR association analysis (conditioned on secondary signal and tertiary signal).

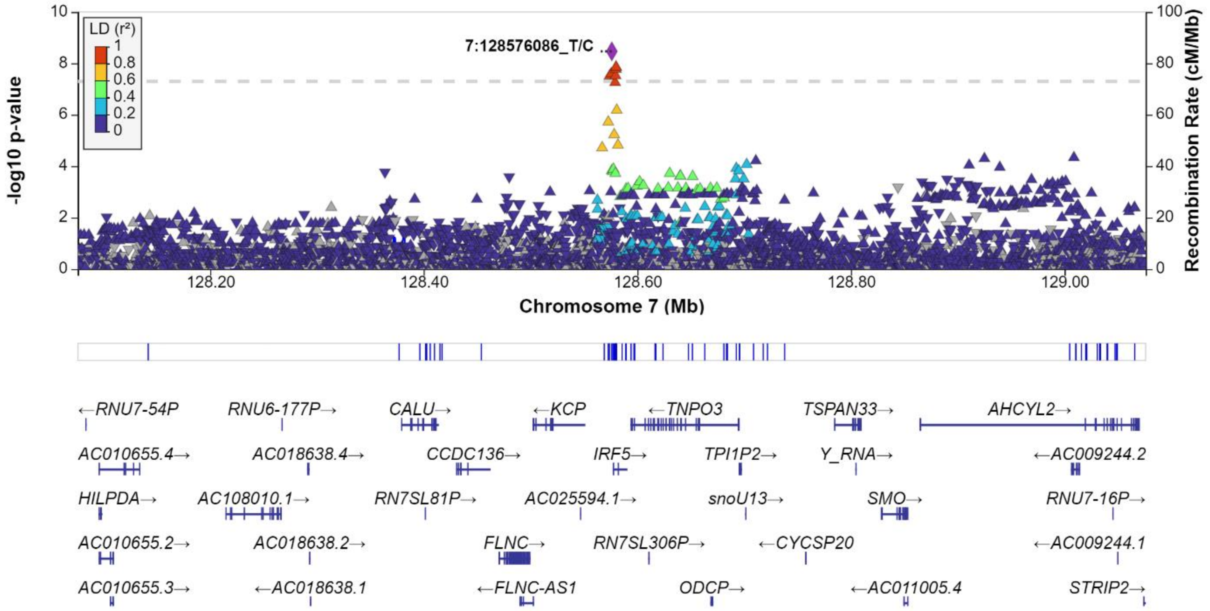

Figure 22: Regional association plot for index SNP rs233438 (11:2794392_G/A) and tertiary signal rs81205 (11: 2798804_A/C) using eGFR association analysis.

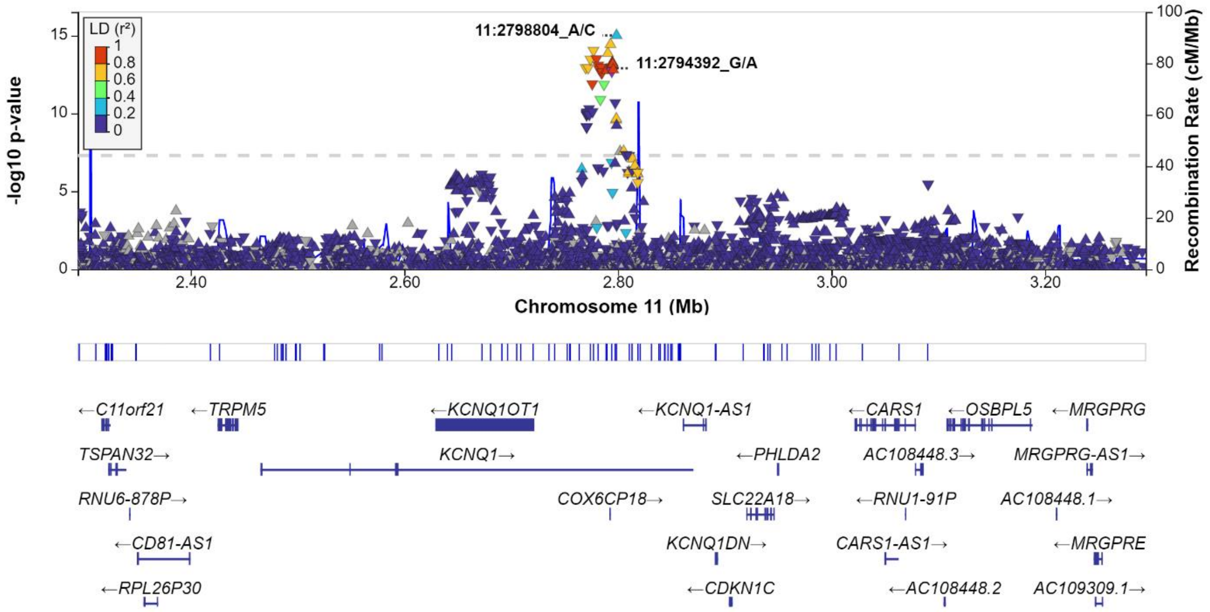

Figure 23: Regional association plot for tertiary signal rs81205 (11:2798804_A/C) using conditional eGFR association analysis (conditioned on index SNP secondary signal and signal of 4th).

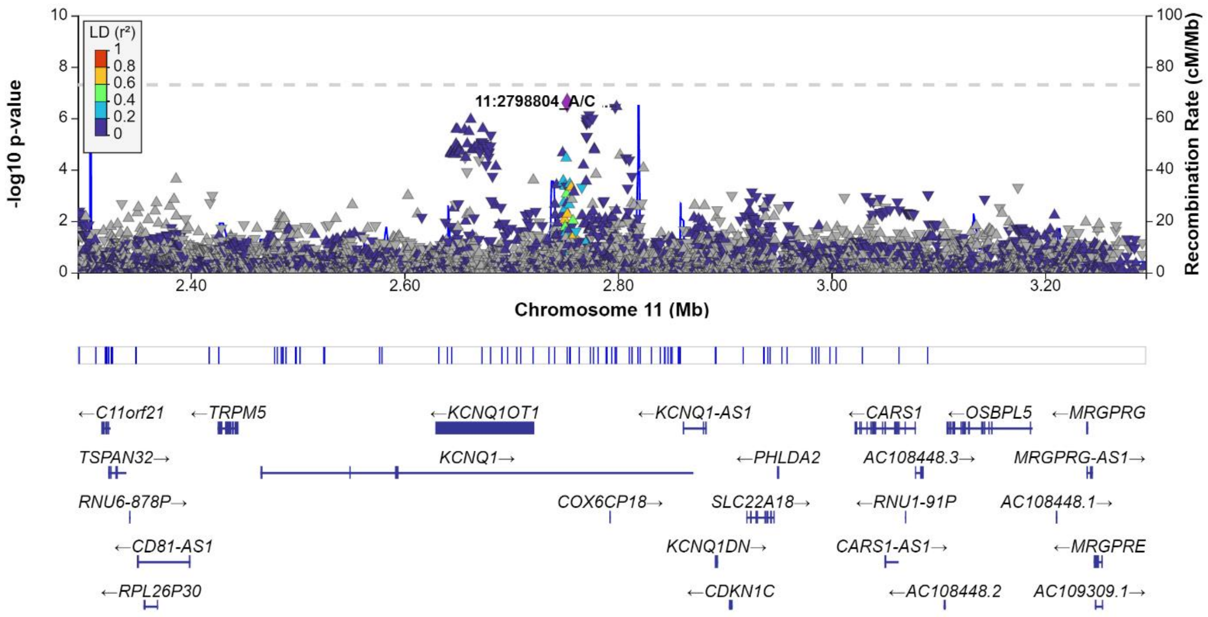

Figure 24: Regional association plot for index SNP rs233438 (11:2794392_G/A) using conditional eGFR association analysis (conditioned on secondary signal, tertiary signal, signal of 4th).

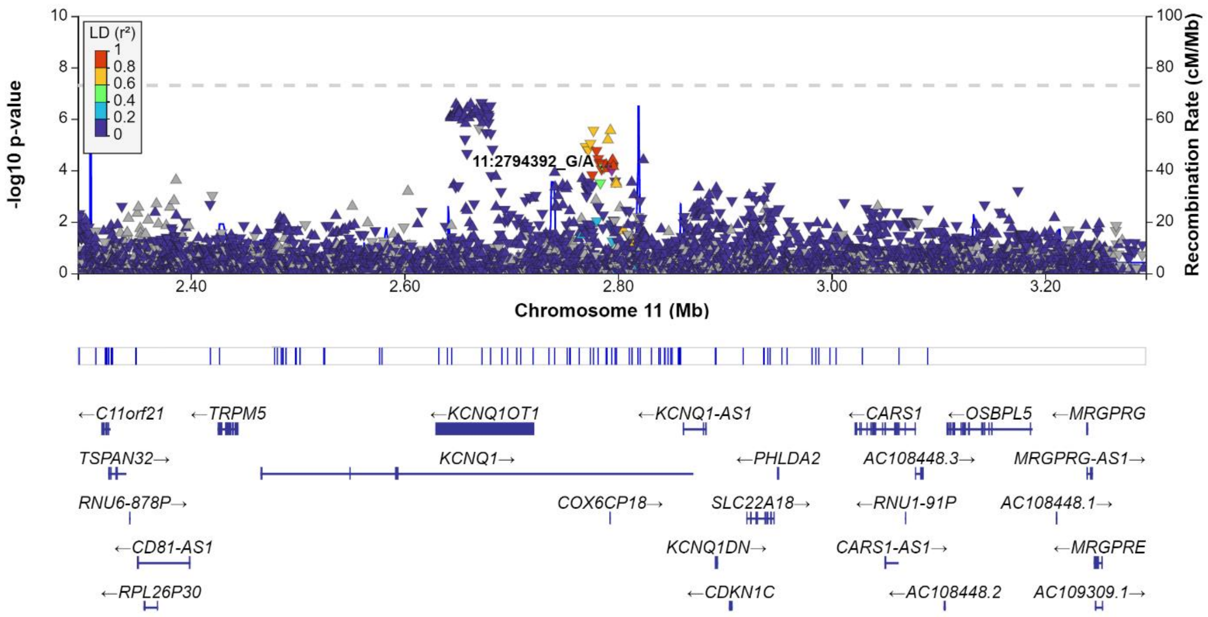

Figure 25: Regional association plot for index SNP rs11227260 (11: 65461158_G/T) and tertiary signal rs1193692 (11: 65504069_A/C) using eGFR association analysis.

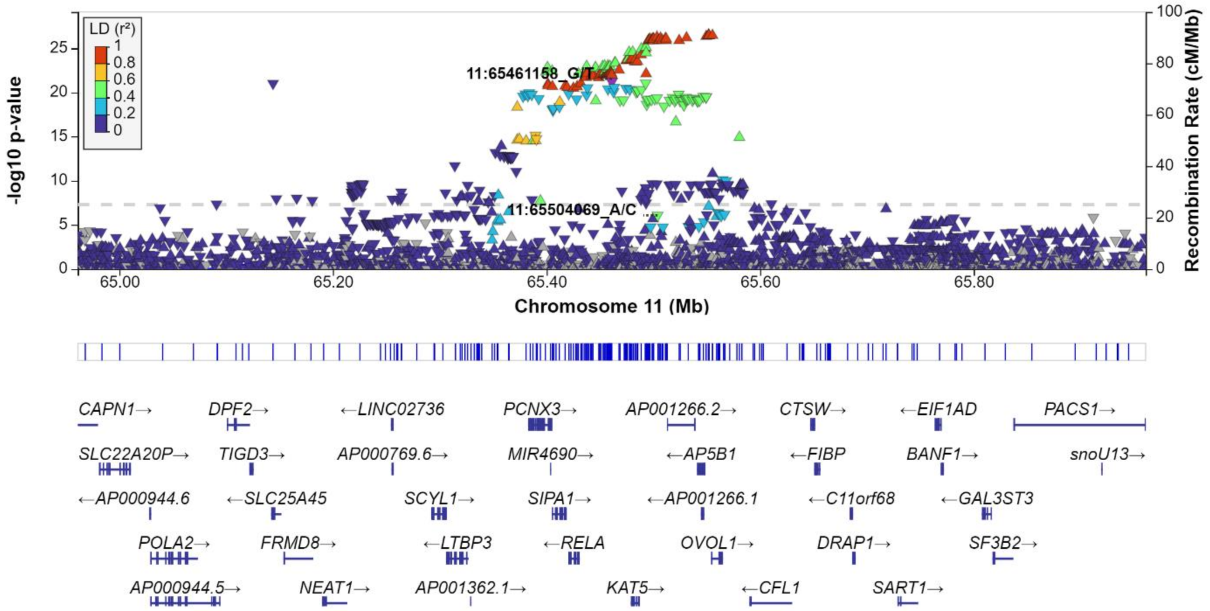

Figure 26: Regional association plot for tertiary signal rs1193692 (11: 65504069_A/C) using conditional eGFR association analysis (conditioned on index SNP and secondary signal).

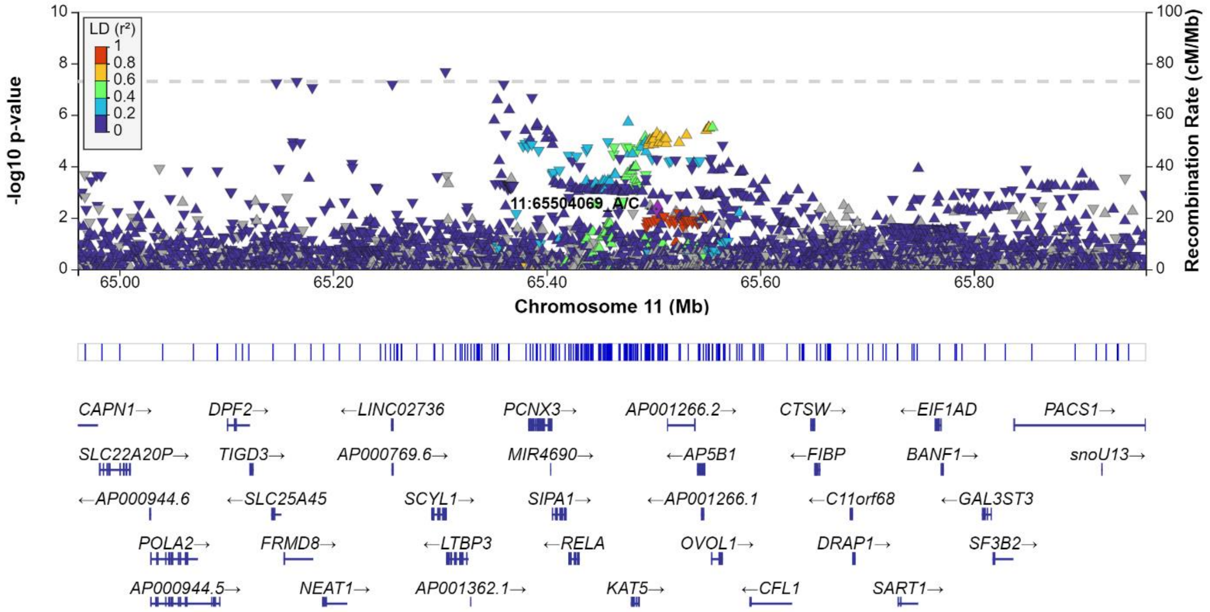

Figure 27: Regional association plot for index SNP rs11227260 (11: 65461158_G/T) using conditional eGFR association analysis (conditioned on secondary signal and tertiary signal).

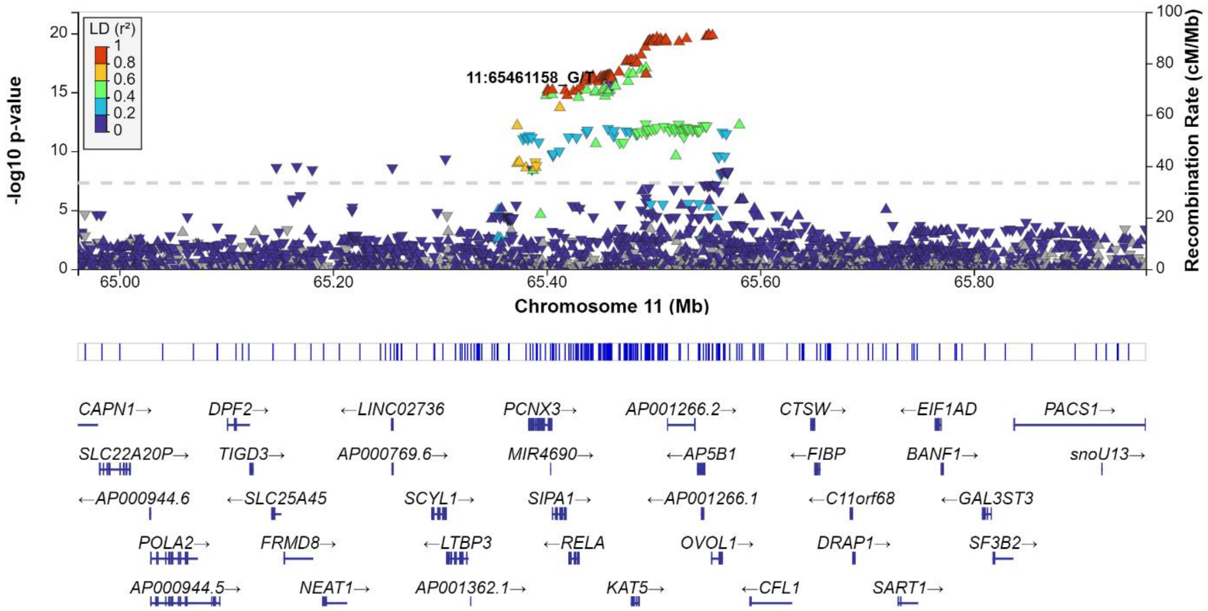

Figure 28: Regional association plot for index SNP rs1153855 (15: 45660758_C/G) and tertiary signal rs4775830 (15: 45533344_A/G) using eGFR association analysis.

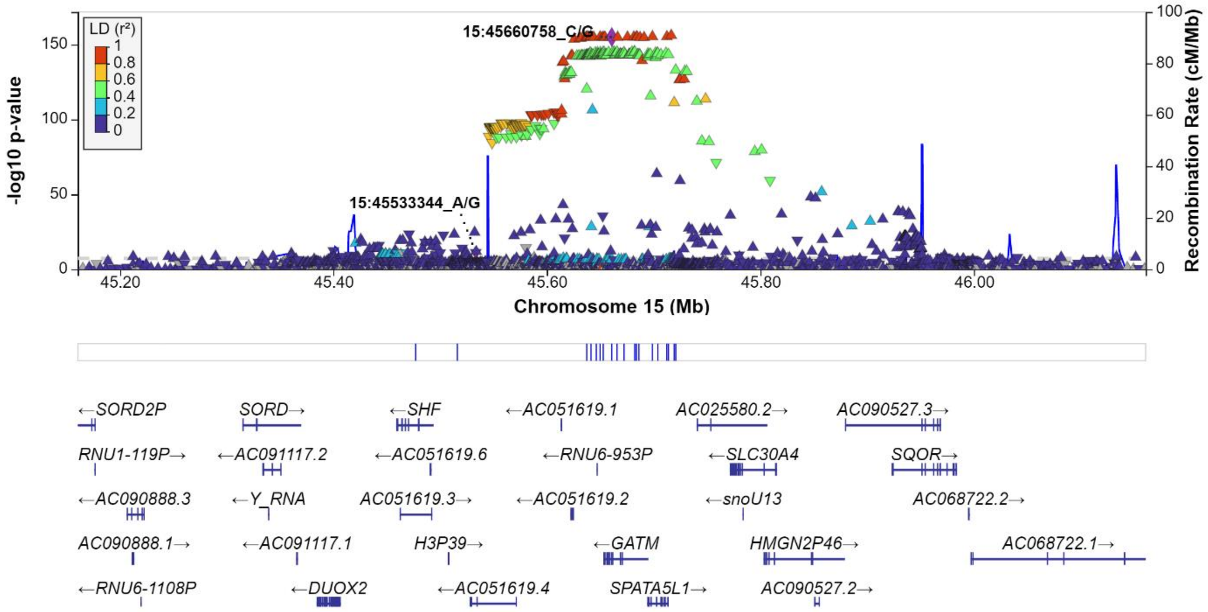

Figure 29: Regional association plot for tertiary signal rs4775830 (15: 45533344_A/G) using conditional eGFR association analysis (conditioned on index SNP and secondary signal).

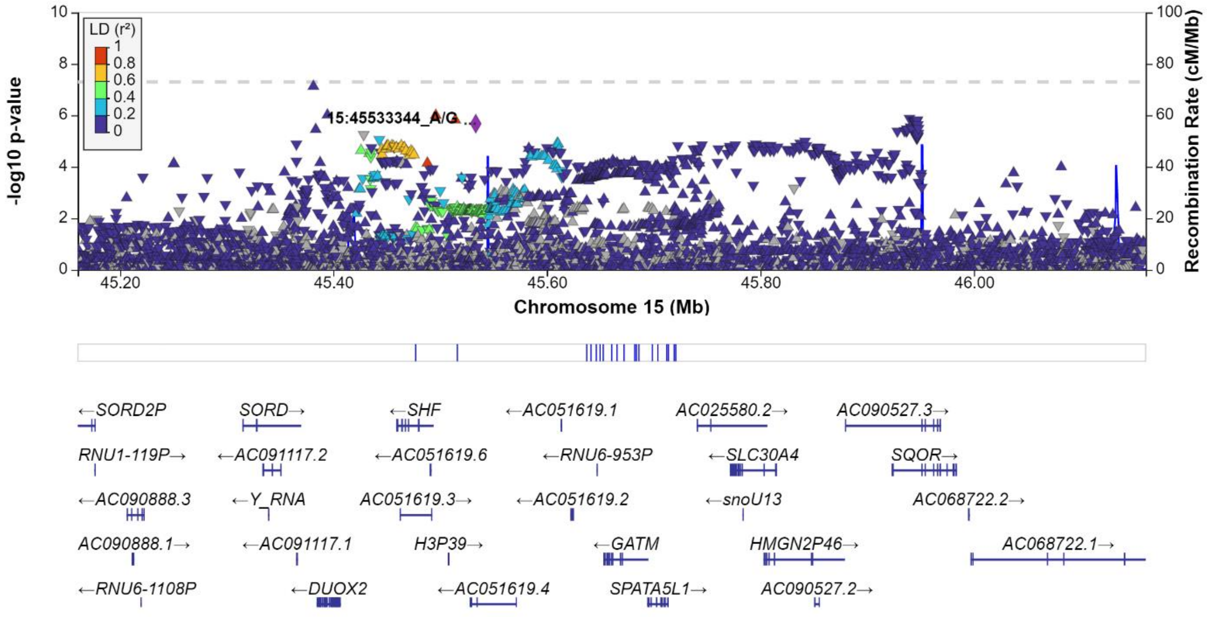

Figure 30: Regional association plot for index SNP rs1153855 (15: 45660758_C/G) using conditional eGFR association analysis (conditioned on secondary signal and tertiary signal).

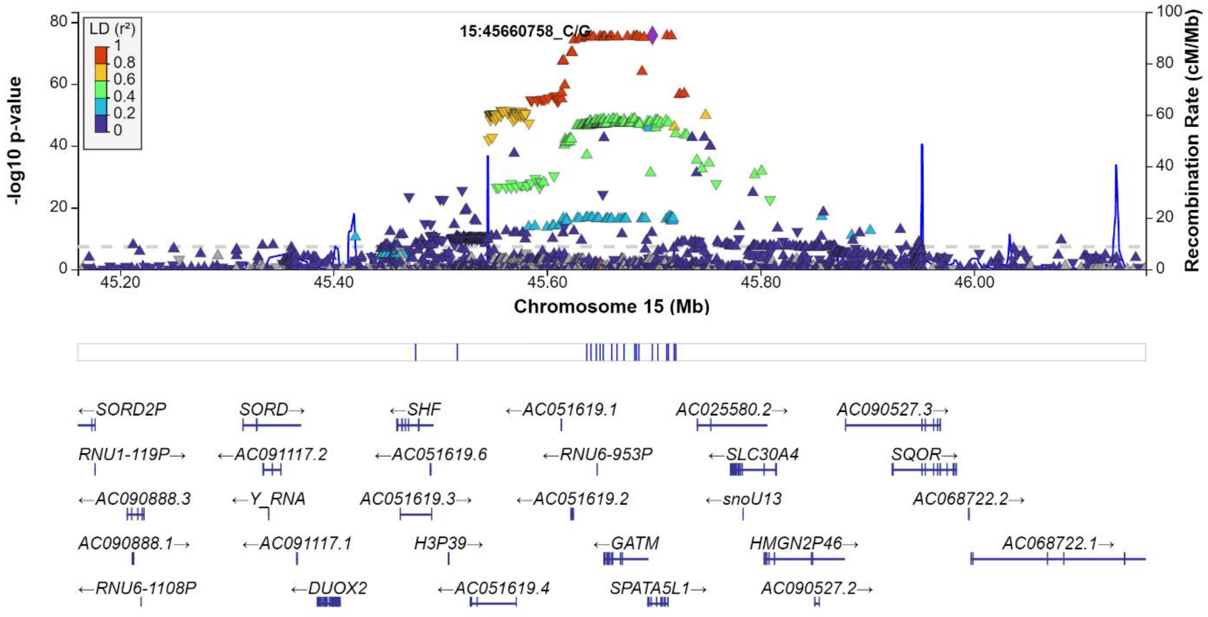

Figure 31: Regional association plot for index SNP rs35472707 (2:169995581_C/T) and signal of 4th rs2075251 (2: 170011458_T/A) using eGFR association analysis.

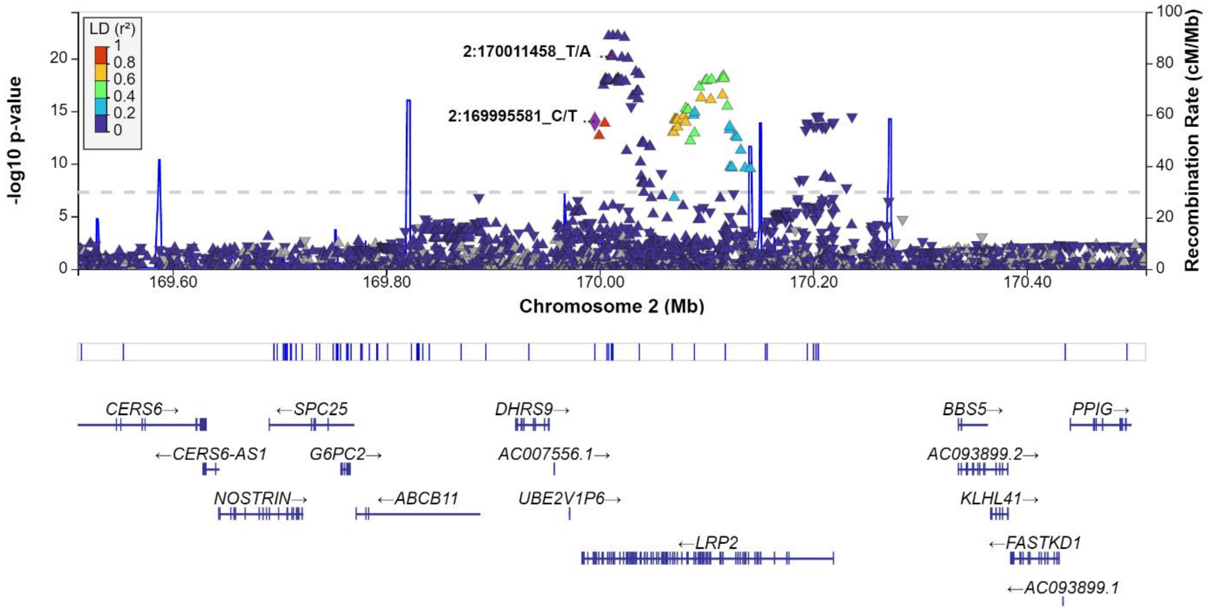

Figure 32: Regional association plot for signal of 4th rs2075251 (2:170011458_T/A) using conditional eGFR association analysis (conditioned on index SNP, secondary signal, and tertiary signal).

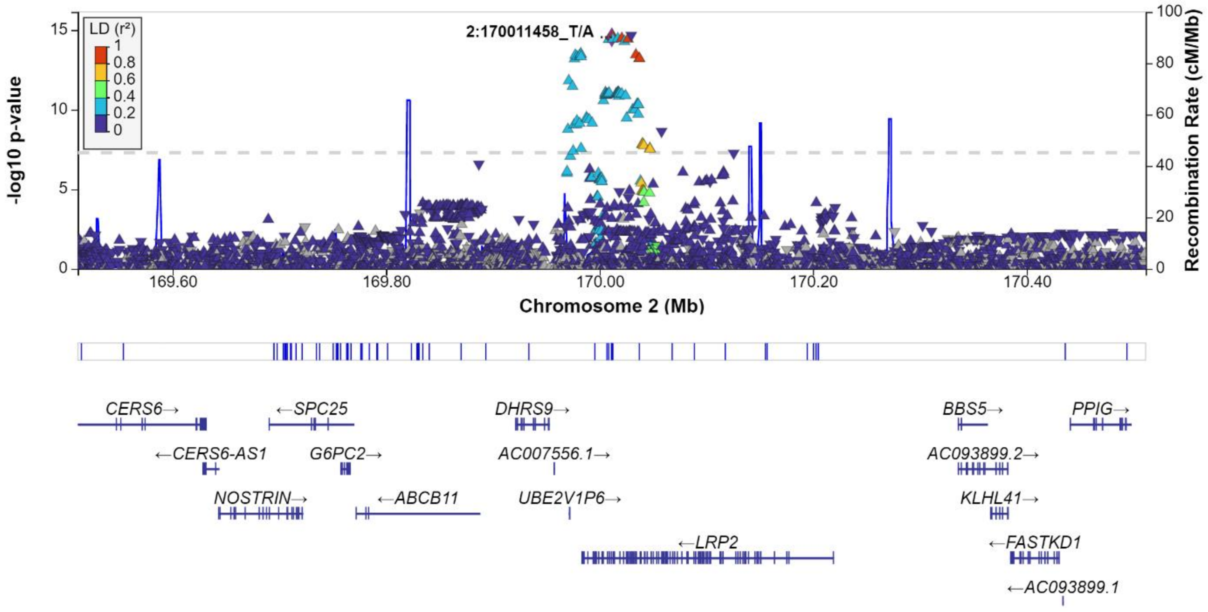

Figure 33: Regional association plot for index SNP rs35472707 (2:169995581_C/T) using conditional eGFR association analysis (conditioned on secondary signal, tertiary signal, and signal of 4th).

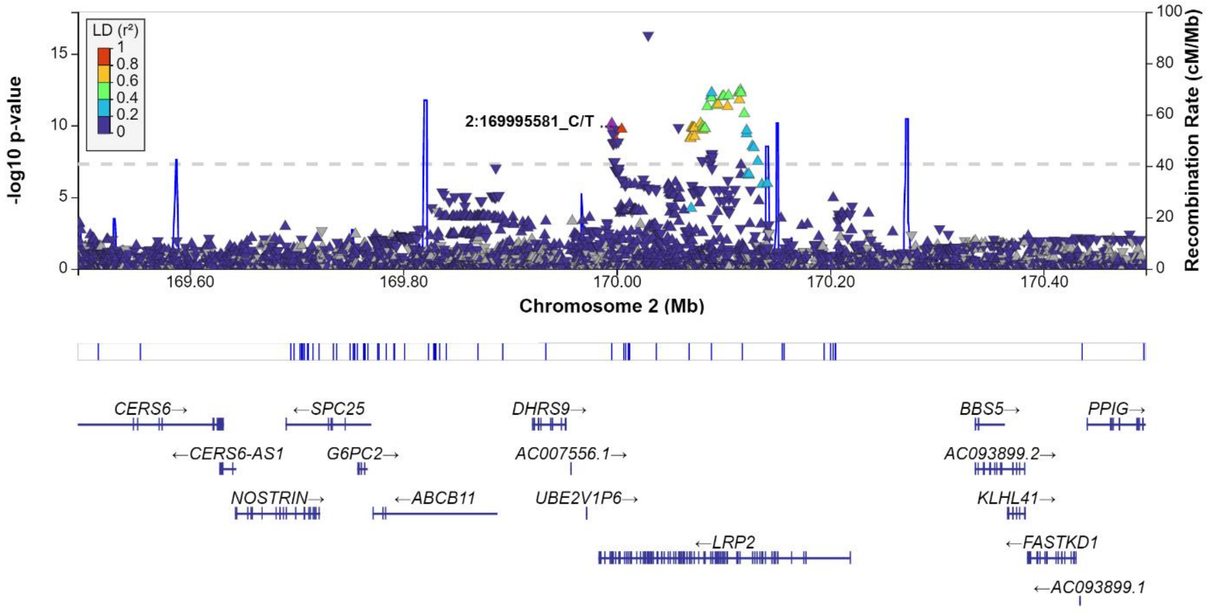

Figure 34: Regional association plot for index SNP rs881858 (6:43806609_G/A) and signal of 4th rs6912283 (6:43364494_G/A) using eGFR association analysis.

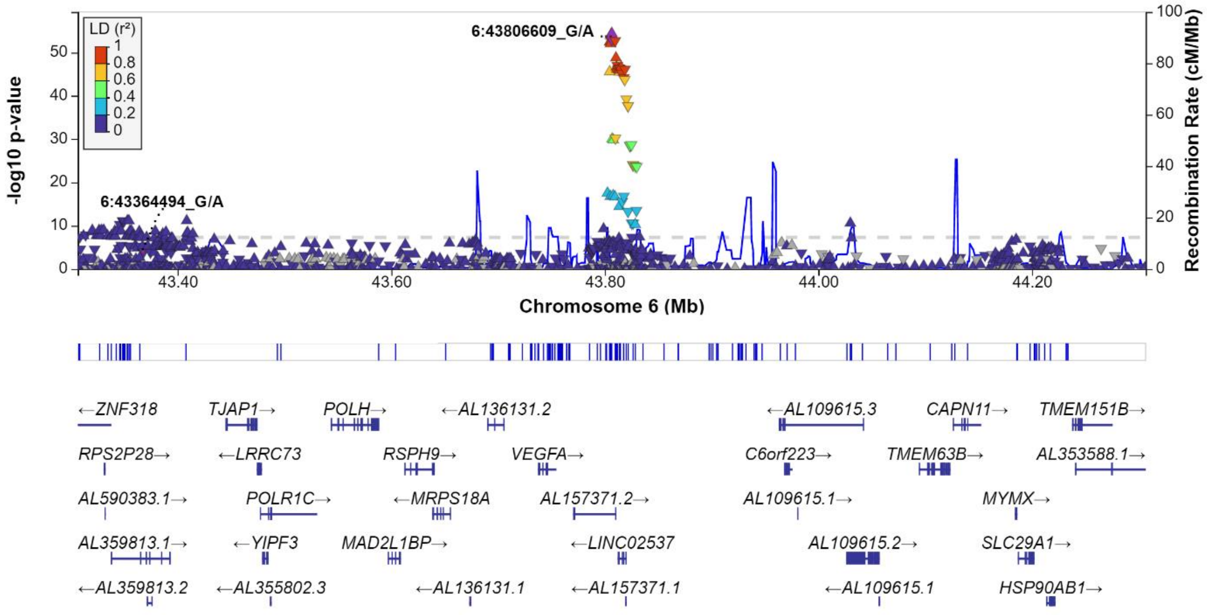

Figure 35: Regional association plot for signal of 4th rs6912283 (6:43364494_G/A) using conditional eGFR association analysis (conditioned on index SNP, secondary signal, and tertiary signal).

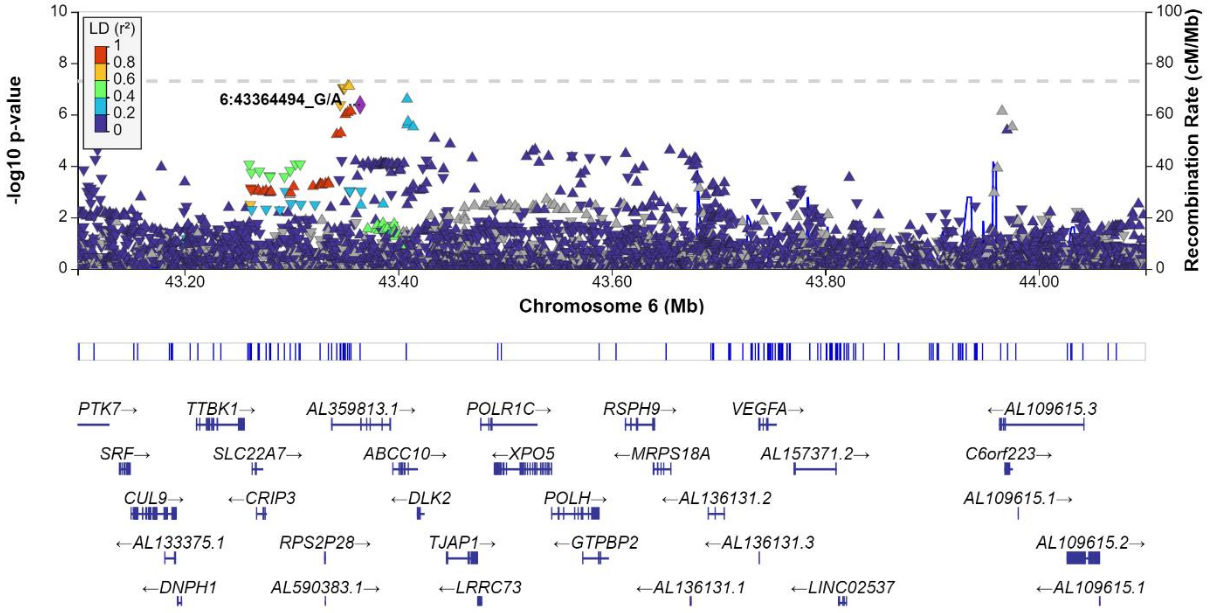

Figure 36: Regional association plot for index SNP rs881858 (6:43806609_G/A) using conditional eGFR association analysis (conditioned on secondary signal, tertiary signal, and signal of 4th).

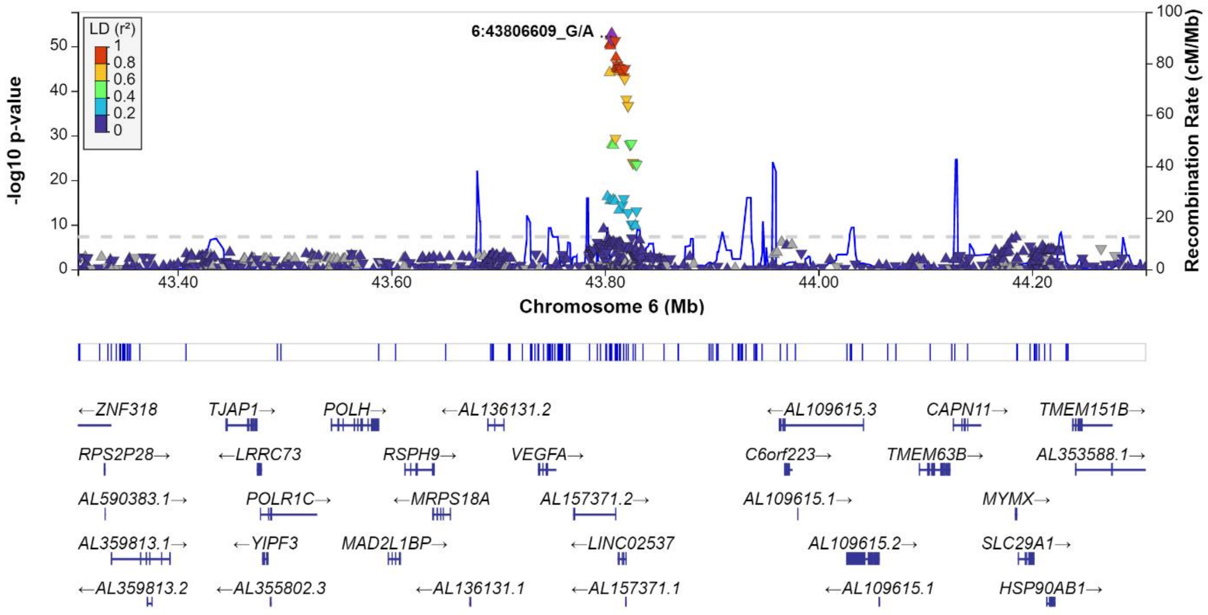

Figure 37: Regional association plot for index SNP rs2039424 (9:71432174_G/A) and signal of 4th rs4745268 (9:71405525_C/T) using eGFR association analysis.

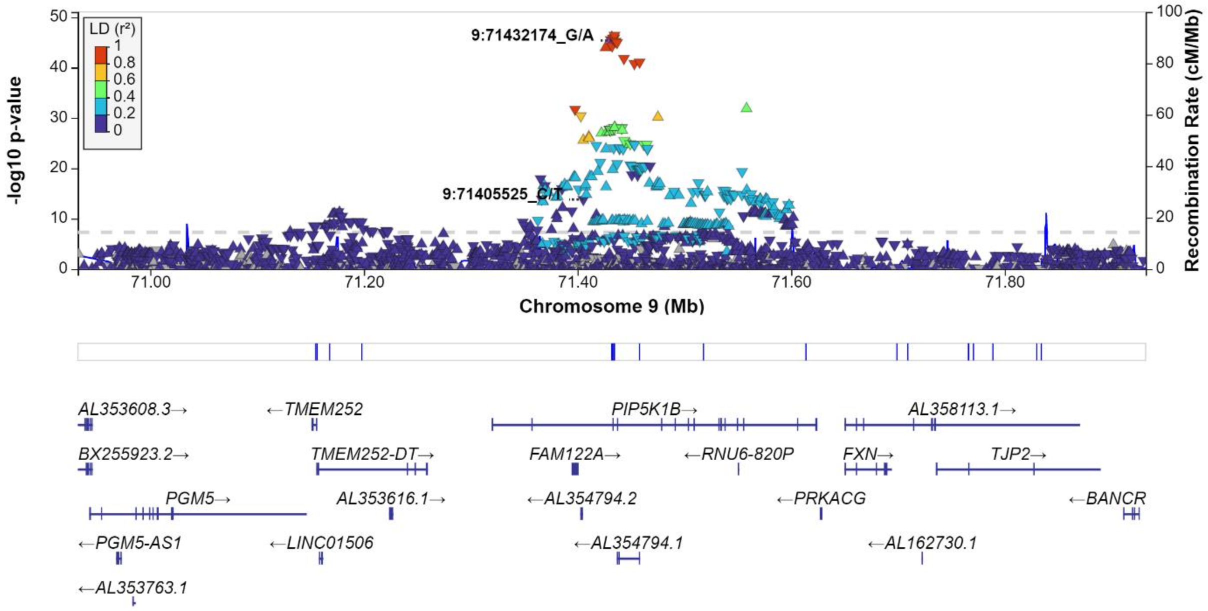

Figure 38: Regional association plot for signal of 4th rs4745268 (9:71405525_C/T) using conditional eGFR association analysis (conditioned on index SNP and secondary signal).

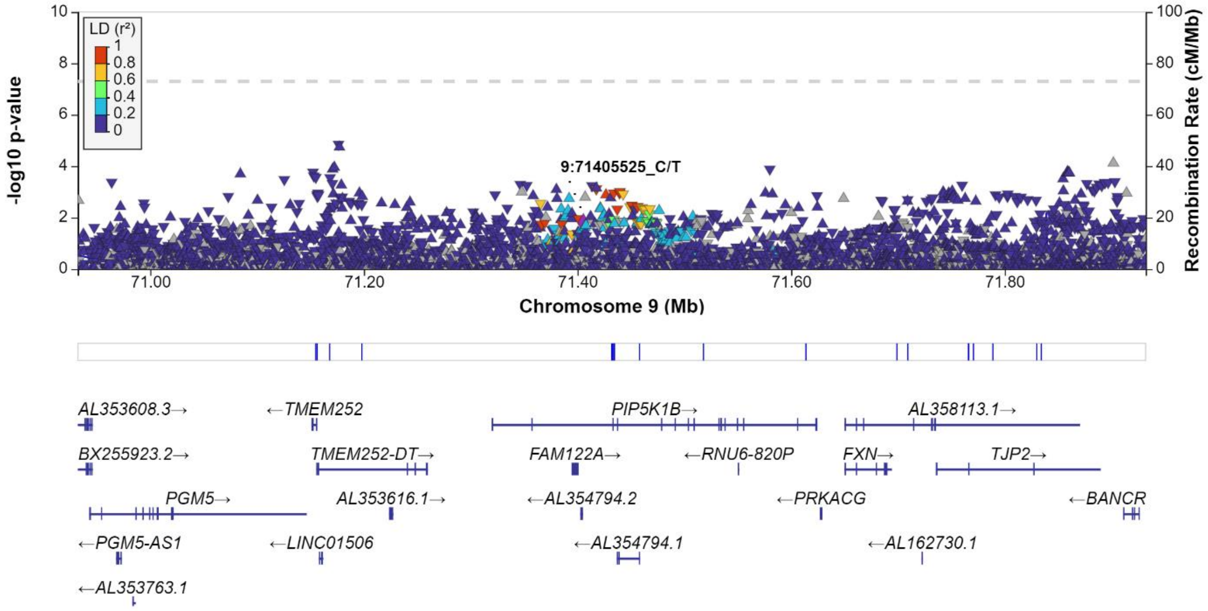

Figure 39: Regional association plot for index SNP rs2039424 (9:71432174_G/A) using conditional eGFR association analysis (conditioned on secondary signal and signal of 4th).

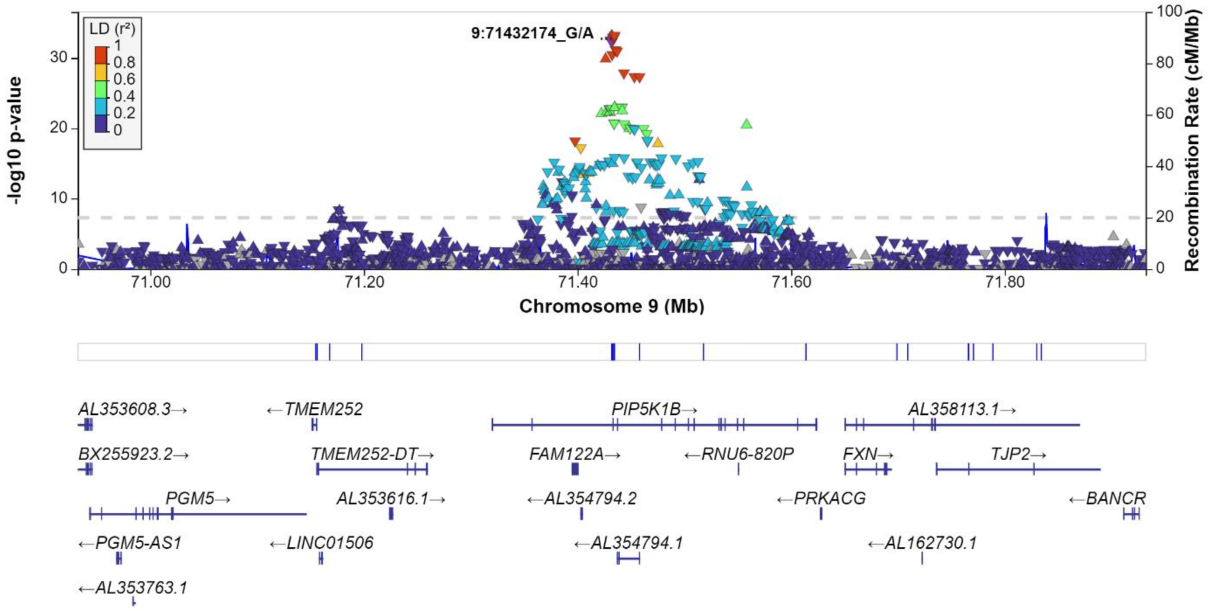

Figure 40: Regional association plot for index SNP rs233438 (11:2794392_G/A) and signal of 4th rs1056819 (11:2949861_C/T) using eGFR association analysis.

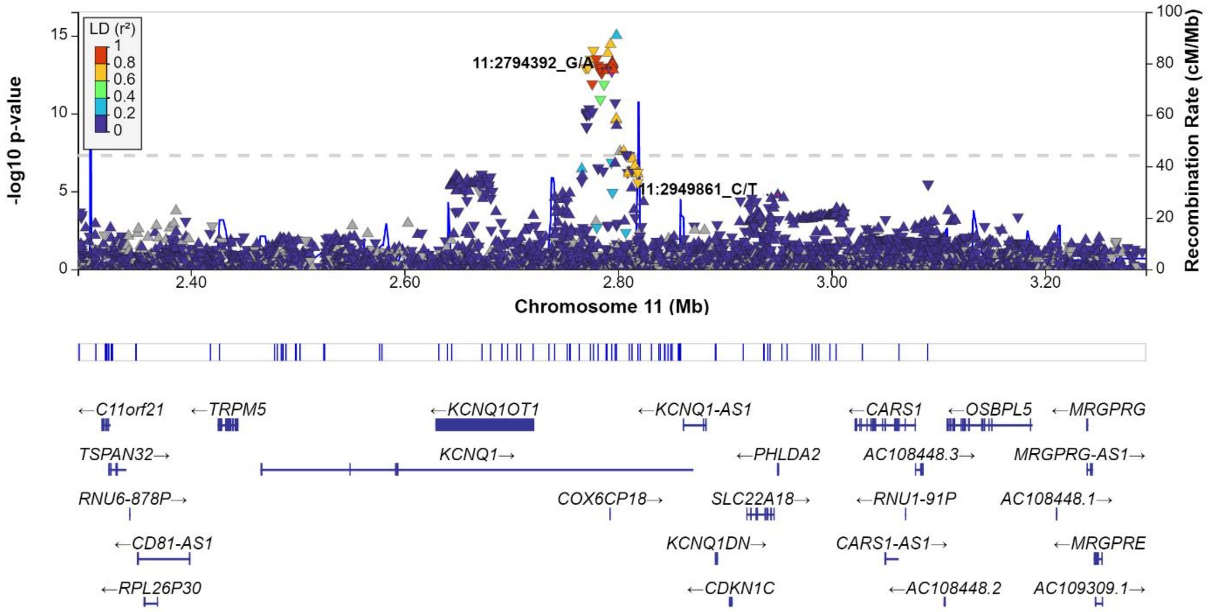

Figure 41: Regional association plot for signal of 4th rs1056819 (11:2949861_C/T) using conditional eGFR association analysis (conditioned on index SNP, secondary signal, and tertiary signal).

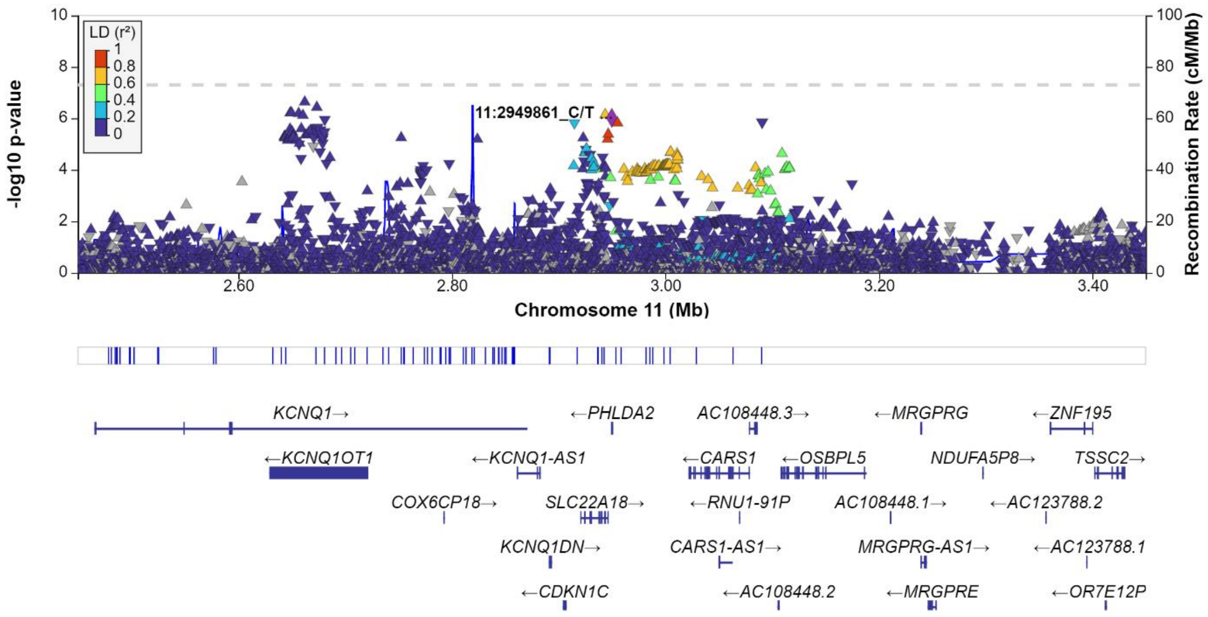

Figure 42: Regional association plot for index SNP rs233438 (11:2794392_G/A) using conditional eGFR association analysis (conditioned on secondary signal, tertiary signal, and signal of 4th).

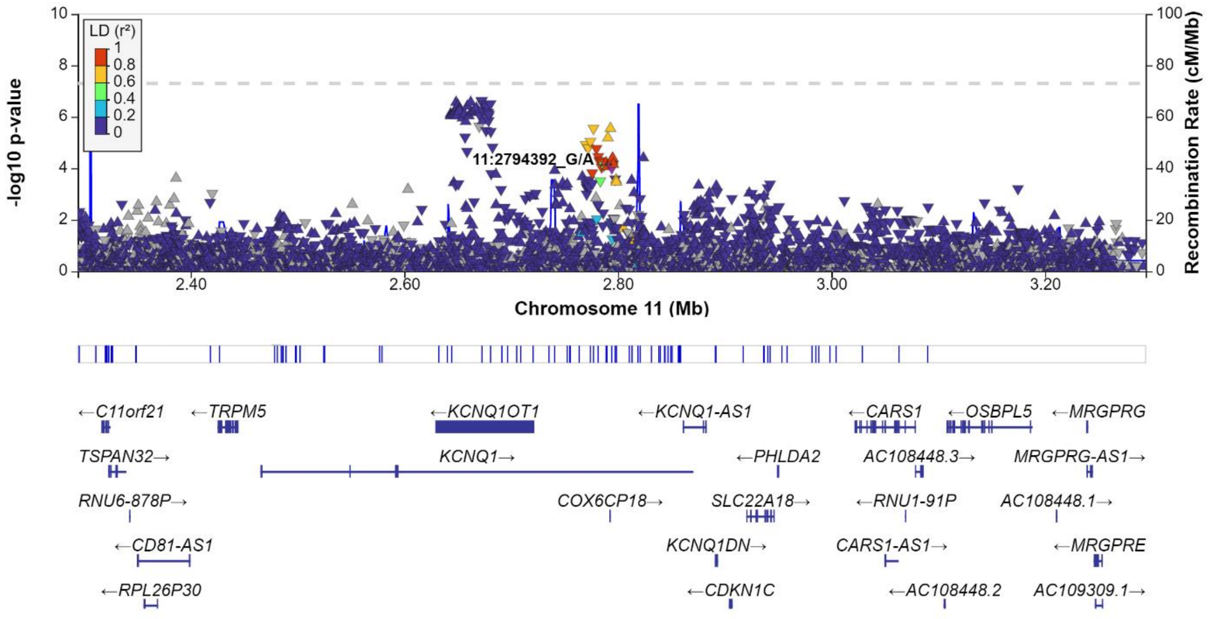

Figure 43: Regional association plot for index SNP rs6127099 (20:52731402_A/T) and signal of 4th rs2585441 (20:52737955_G/C) using eGFR association analysis.

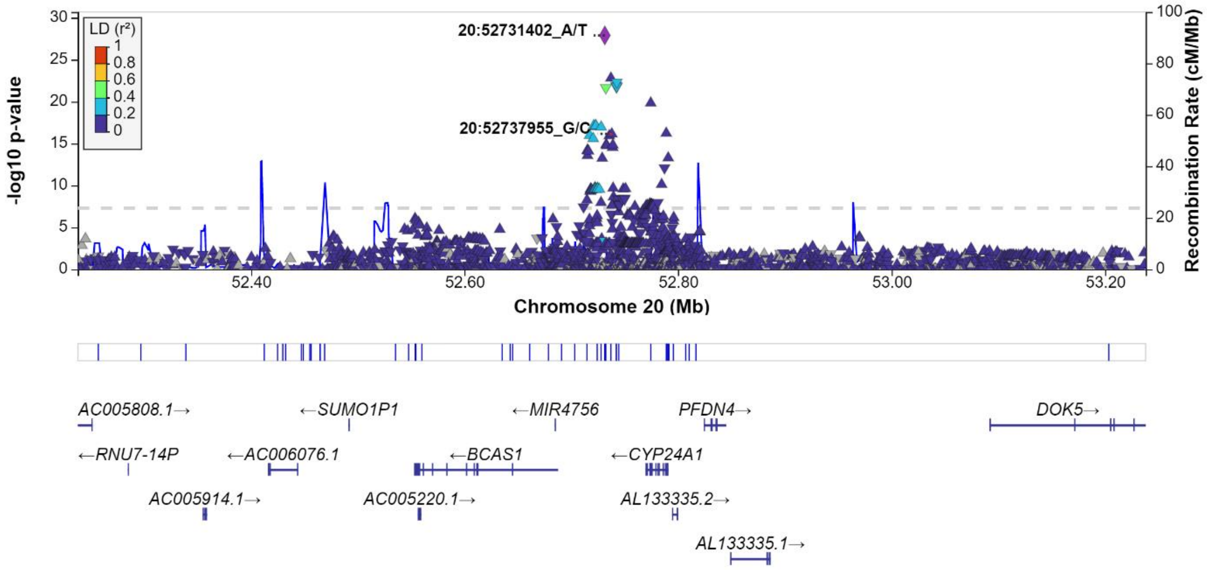

Figure 44: Regional association plot for signal of 4th rs2585441 (20:52737955_G/C) using conditional eGFR association analysis (conditioned on index SNP and secondary signal).

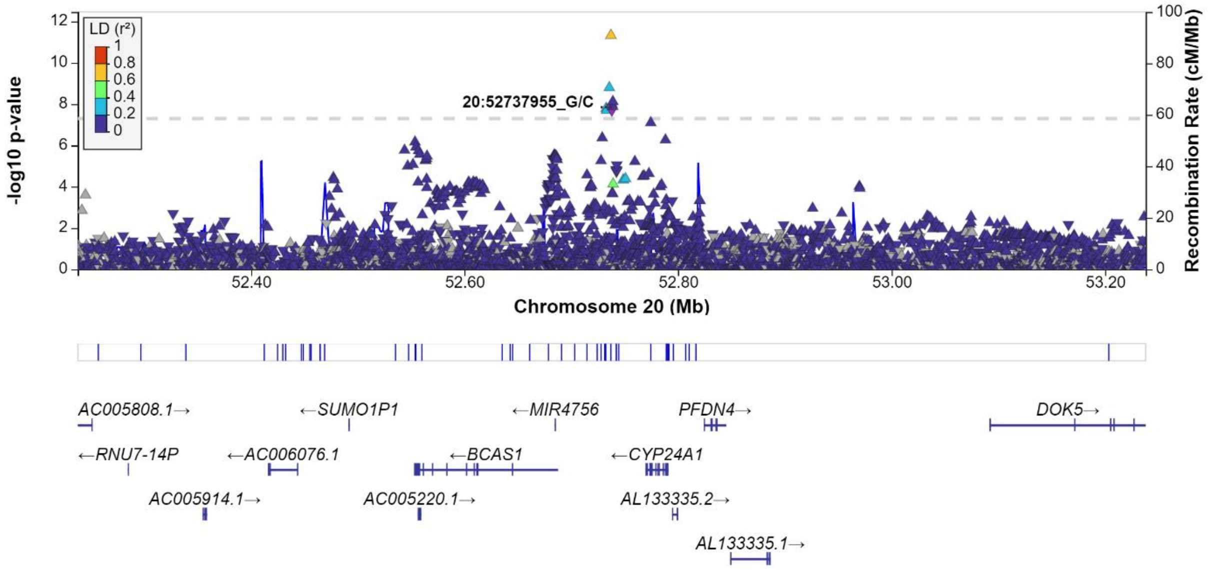

Figure 45: Regional association plot for index SNP rs6127099 (20:52731402_A/T) using conditional eGFR association analysis (conditioned on secondary signal and signal of 4th).

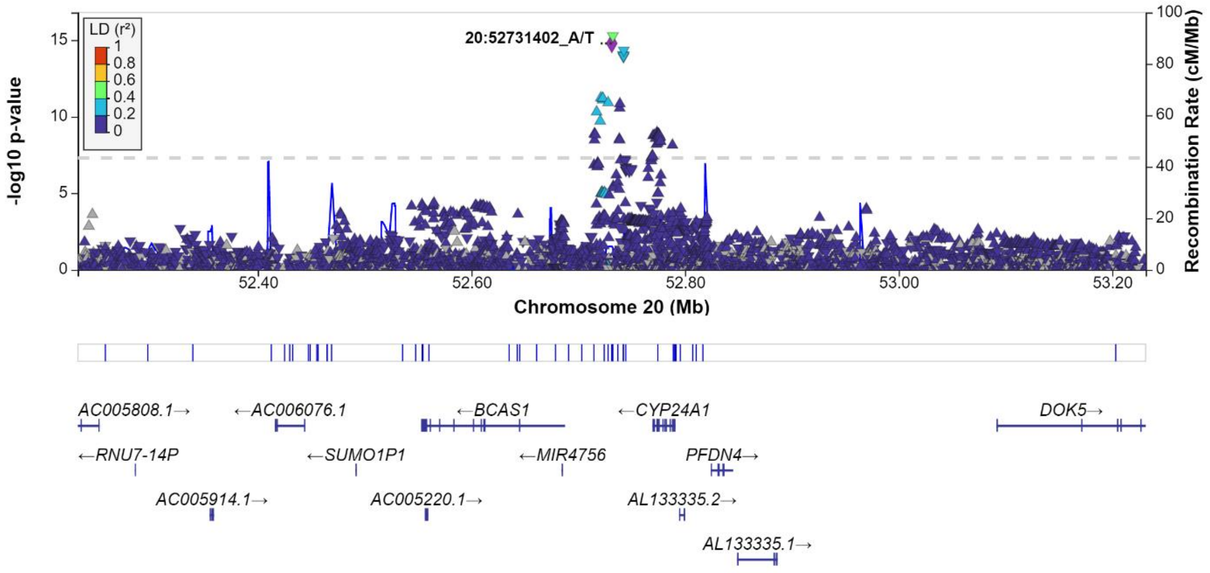

Figure 46: Regional association plot for index SNP rs2261092 (20:62353933_A/G) and signal of 4th rs6062357 (20:62892739_C/T) using eGFR association analysis.

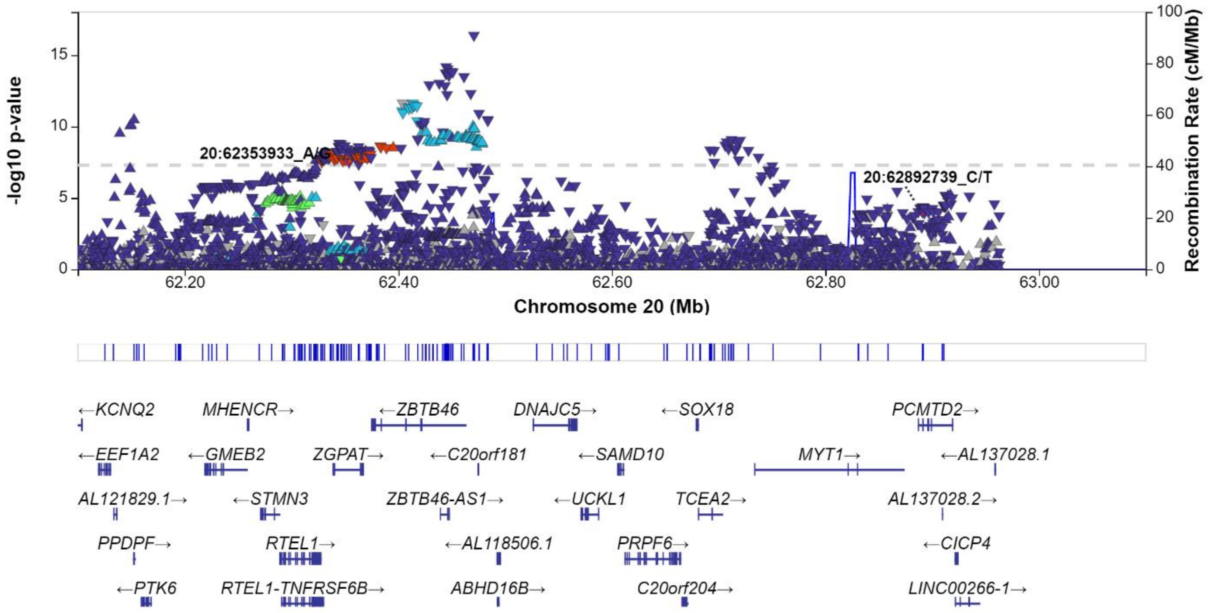

Figure 47: Regional association plot for signal of 4th rs6062357 (20:62892739_C/T) using conditional eGFR association analysis (conditioned on index SNP, secondary signal, and tertiary signal).

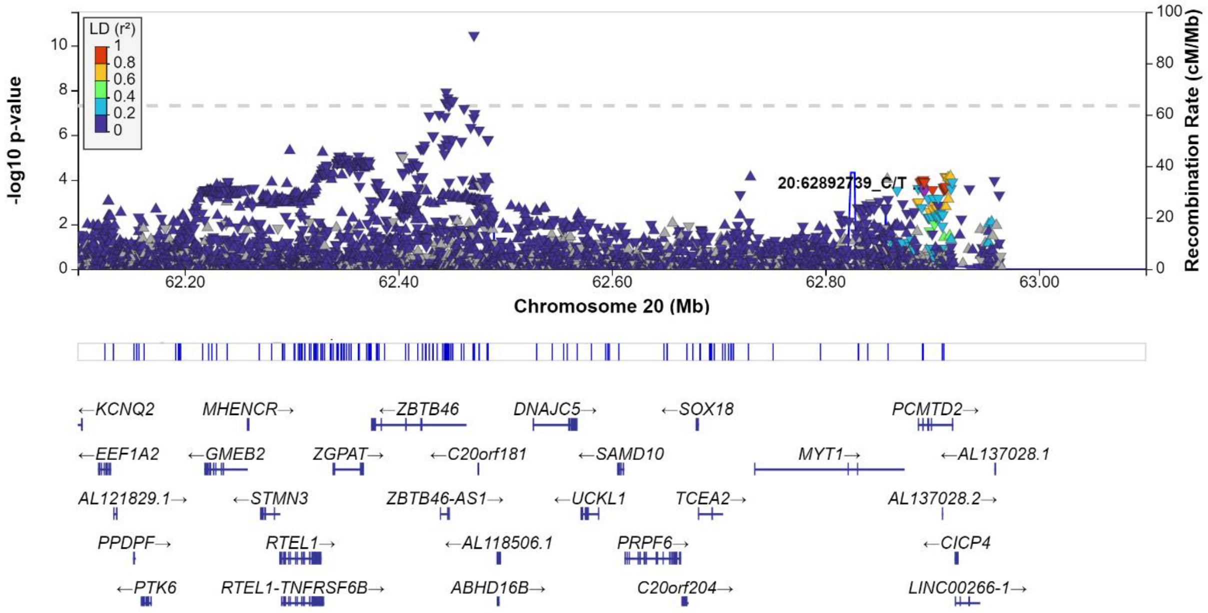

Figure 48: Regional association plot for index SNP rs2261092 (20:62353933_A/G) using conditional eGFR association analysis (conditioned on secondary signal, tertiary signal, and signal of 4th).

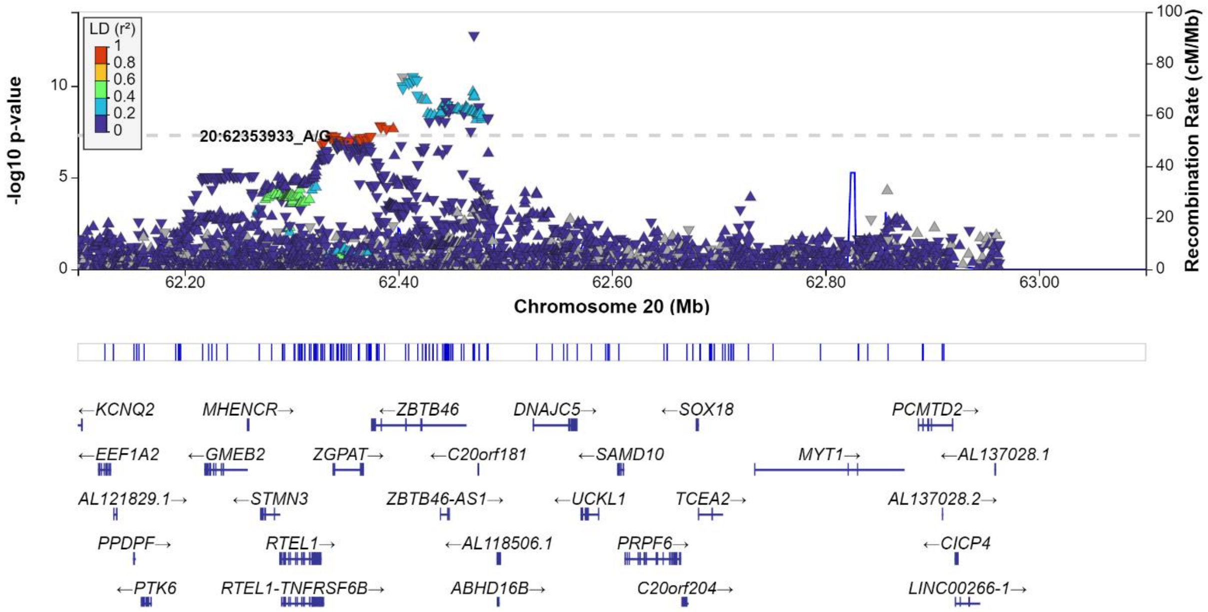

Figure 49: Regional association plot for index SNP rs12207180 (6:160633107_T/A) and signal of 5th rs76426793 (6:160566392_ T/A) using eGFR association analysis.

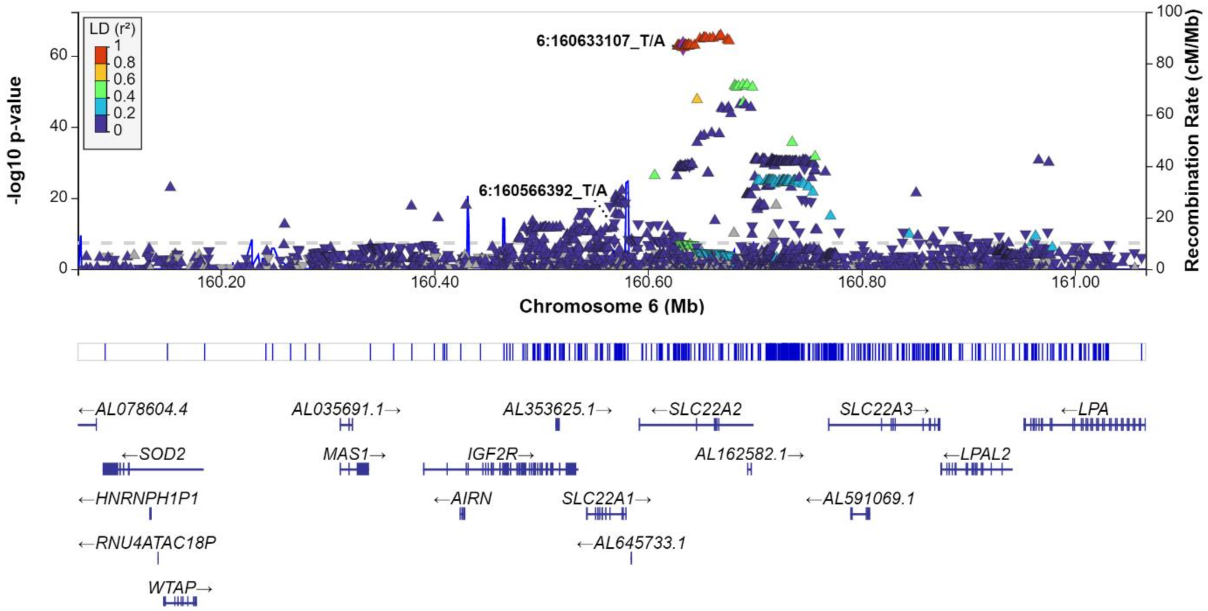

Figure 50: Regional association plot for signal of 5th rs76426793 (6:160566392_ T/A) using conditional eGFR association analysis (conditioned on index SNP, secondary signal, tertiary signal, and signal of 4th).

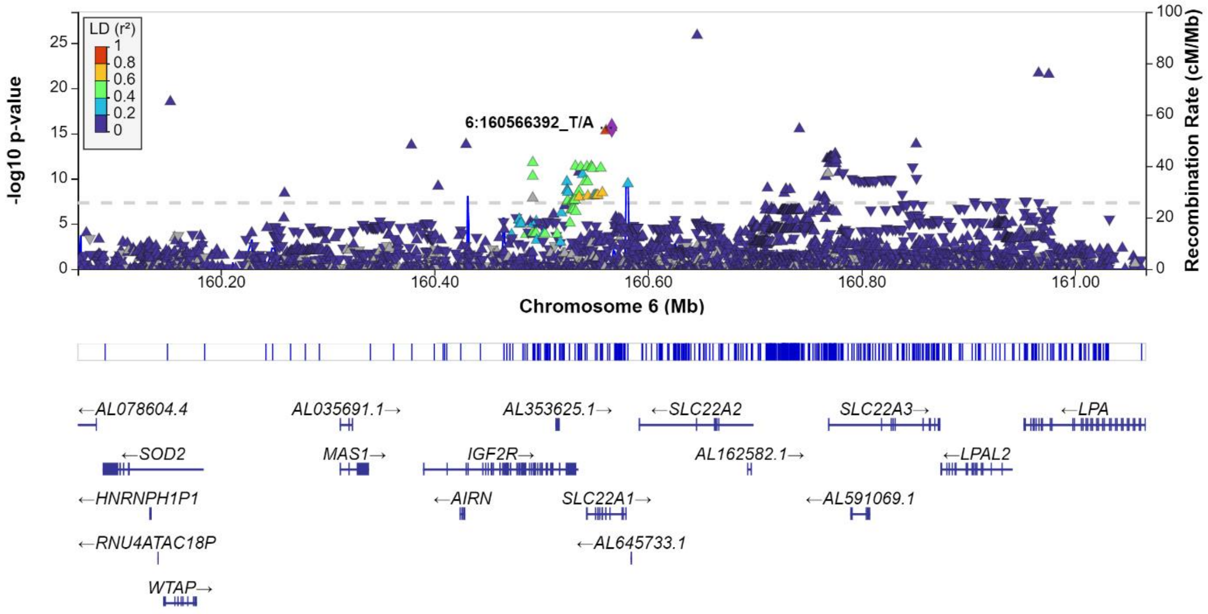

Figure 51: Regional association plot for index SNP rs12207180 (6:160633107_ T/A) using conditional eGFR association analysis (conditioned on secondary signal, tertiary signal, and signal of 4th, and signal of 5th).

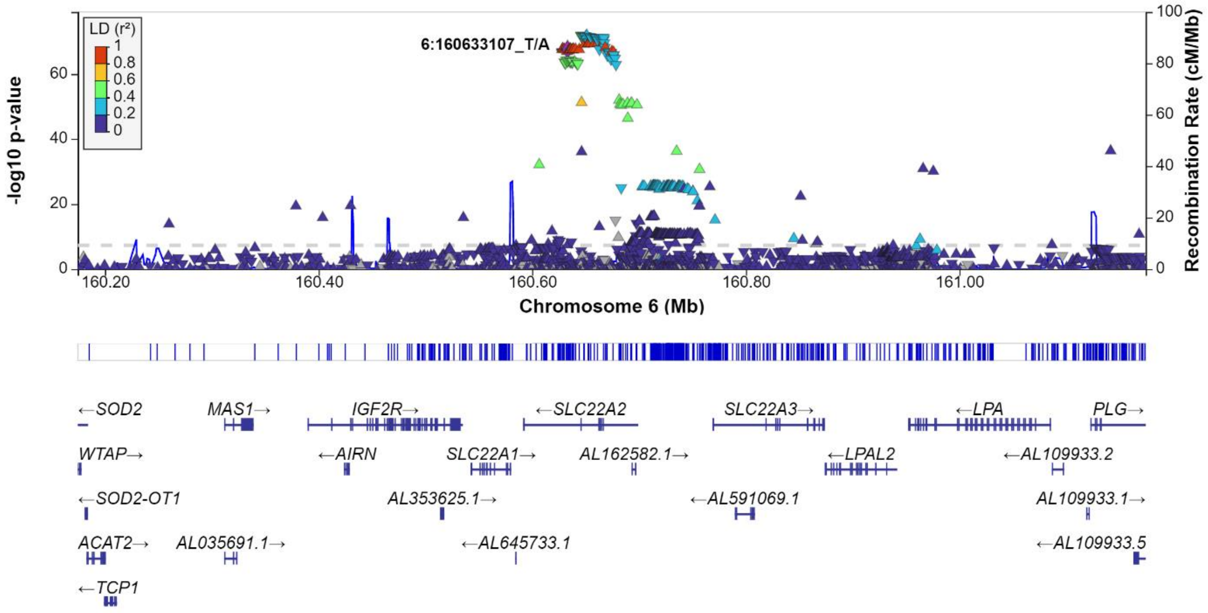

Figure 52: Regional association plot for index SNP rs2365286 (7:156258179_G/A) and signal of 5th rs2695565 (7:156397312_ A/G) using eGFR association analysis.

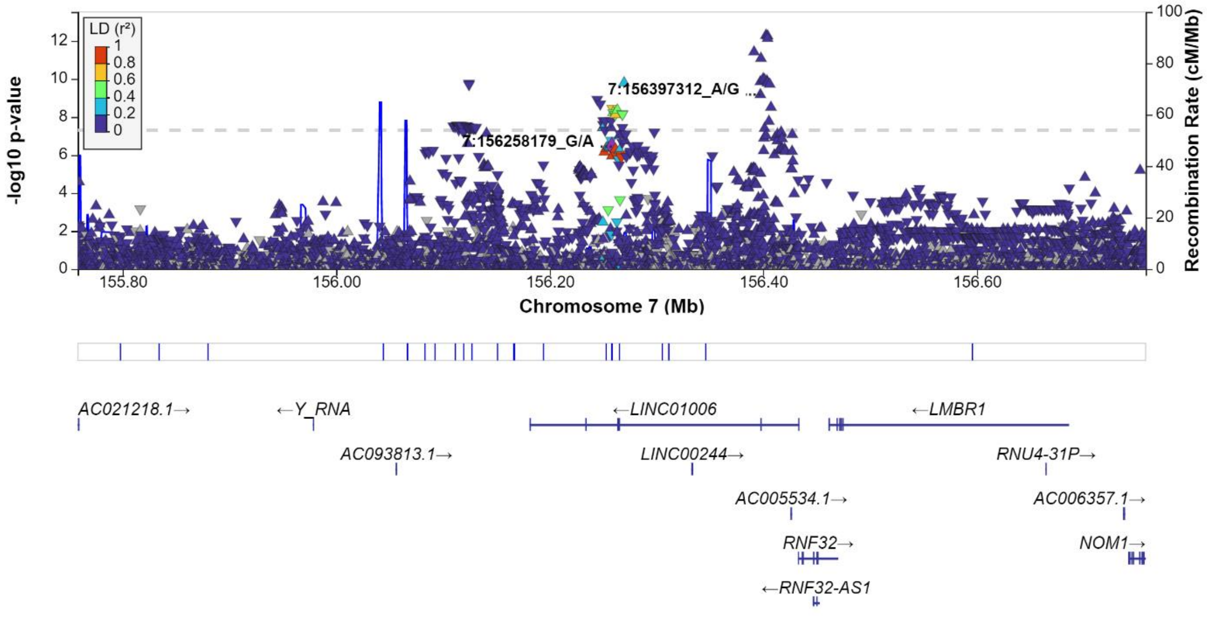

Figure 53: Regional association plot for signal of 5th rs2695565 (7:156397312_ A/G) using conditional eGFR association analysis (conditioned on index SNP, secondary signal, tertiary signal, signal of 4th and signal of 6th).

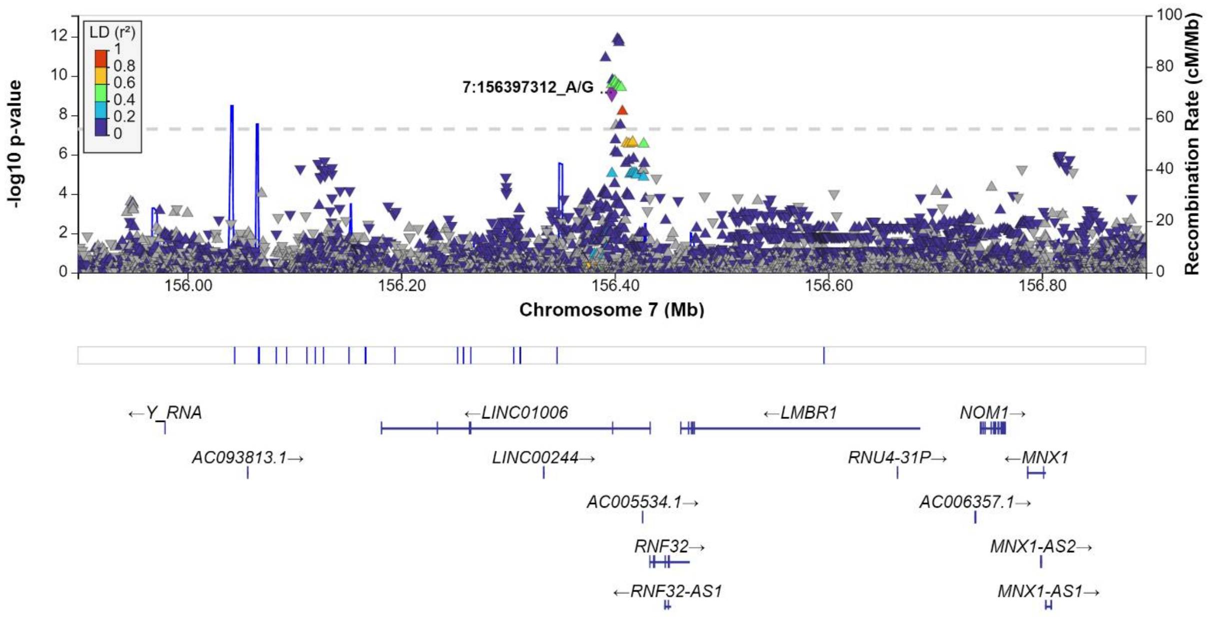

Figure 54: Regional association plot for index SNP rs2365286 (7:156258179_G/A) using conditional eGFR association analysis (conditioned on secondary signal, tertiary signal, signal of 4th, signal of 5th and signal of 6th).

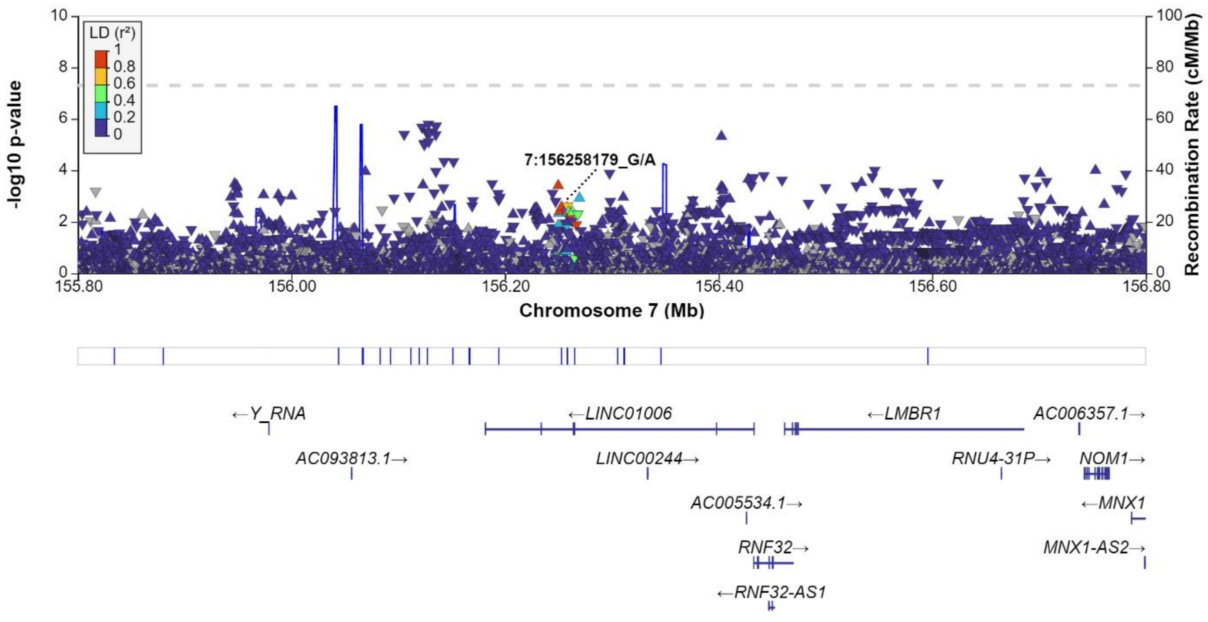

Figure 55: Regional association plot for index SNP rs2365286 (7:156258179_G/A) and signal of 6th rs6951593 (7:156270725_ A/G) using eGFR association analysis.

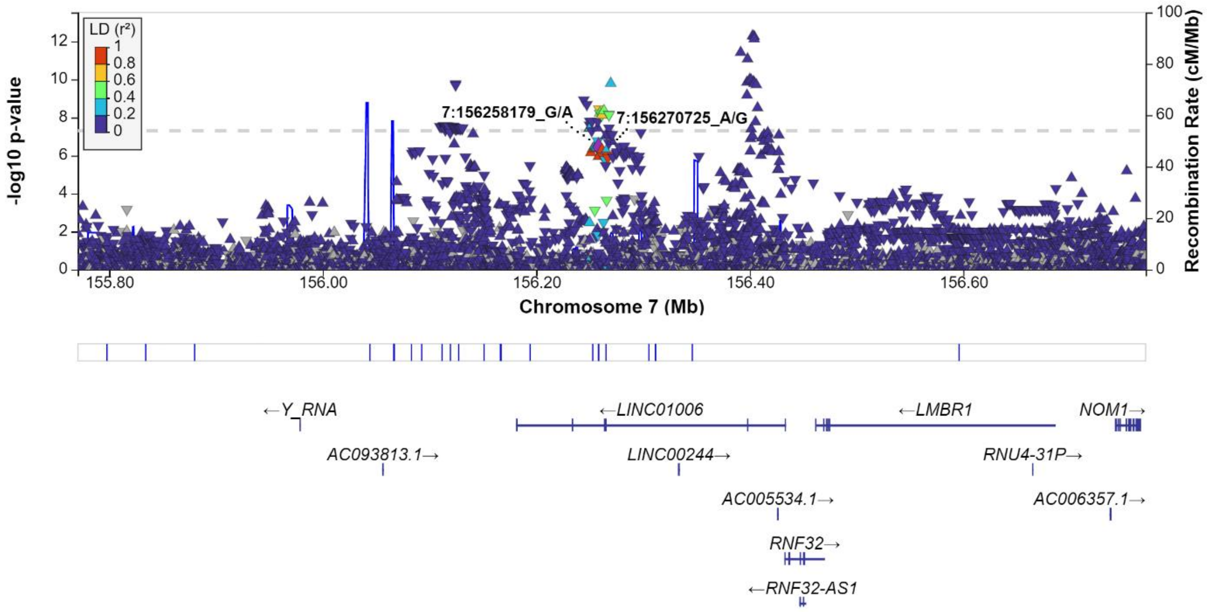

Figure 56: Regional association plot for signal of 6th rs6951593 (7:156270725_ A/G) using conditional eGFR association analysis (conditioned on index SNP, secondary signal, tertiary signal, signal of 4th, and signal of 5th).

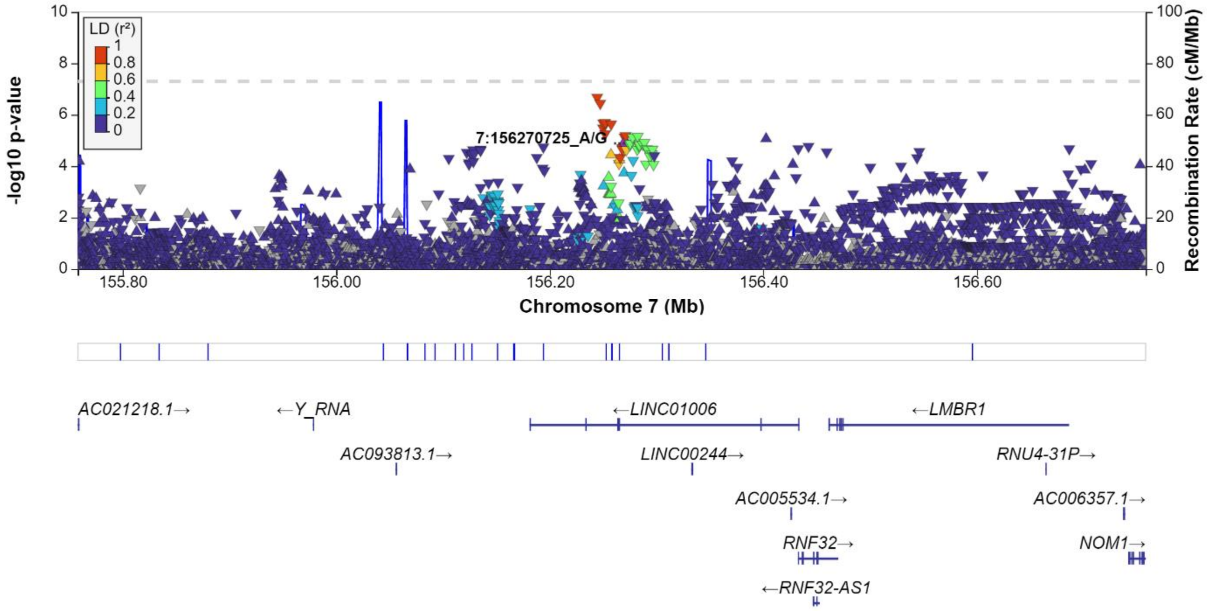

Figure 57: Regional association plot for index SNP rs2365286 (7:156258179_G/A) using conditional eGFR association analysis (conditioned on secondary signal, tertiary signal, signal of 4th, signal of 5th, signal of 6th).

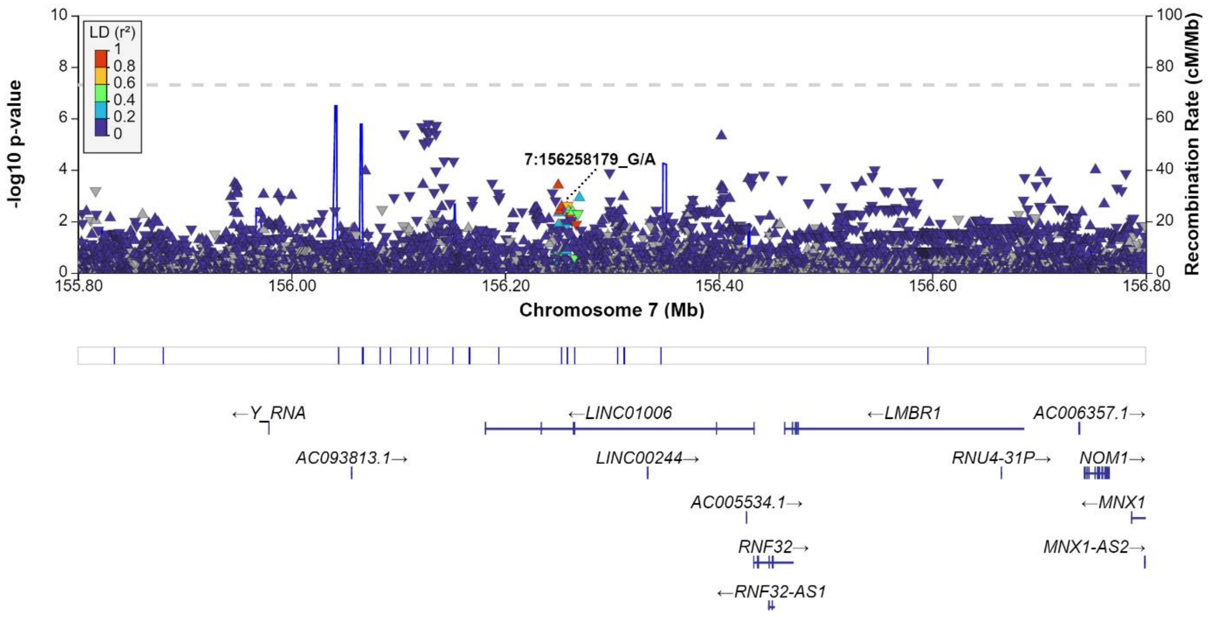

Figure 58: Colocalization of the known index SNPs using unconditional eGFR association analysis.

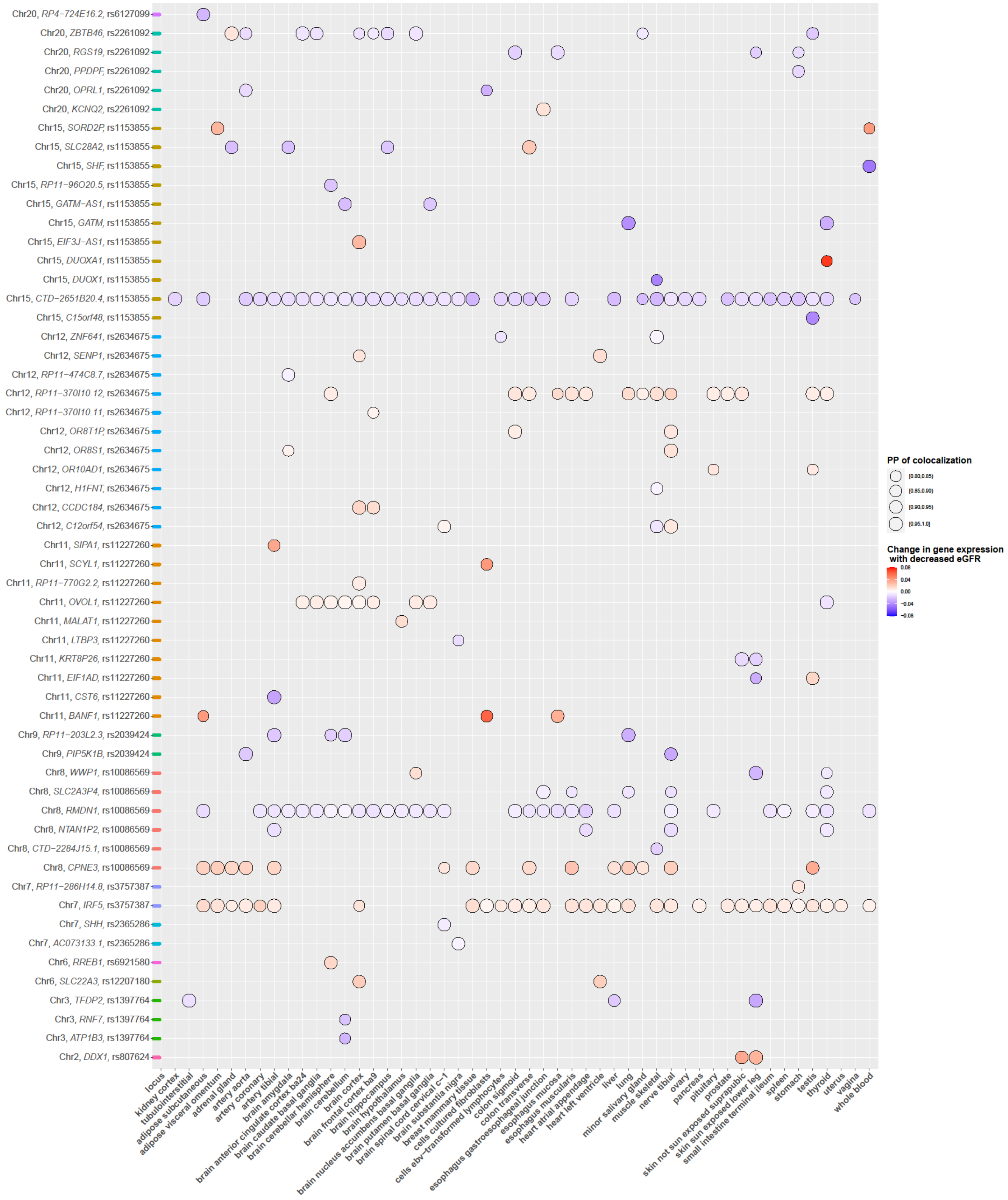

Figure 59: Colocalization of the known index SNPs using conditional eGFR association analysis.

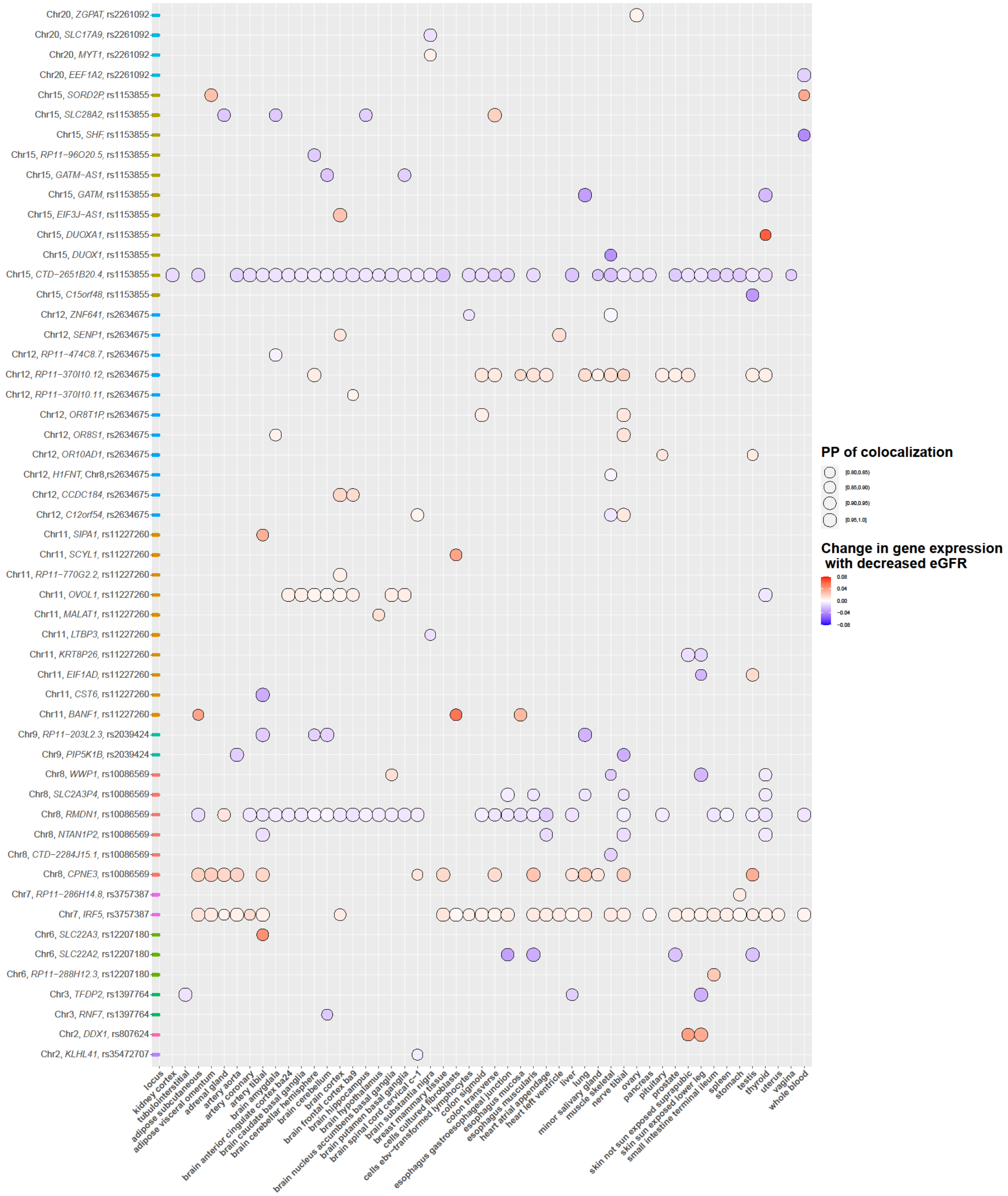

Figure 60: Colocalization results for index SNP rs35472707 and signal of 4th rs2075251 on chromosome 2. **Plot A**: conditional eGFR association analysis adjusted for secondary signal (rs3845731), tertiary signal (rs4668134) and signal of 4th (rs2075251). **Plot B**: conditional eGFR association analysis adjusted for index SNP (rs35472707), secondary signal (rs3845731), and tertiary signal (rs4668134).

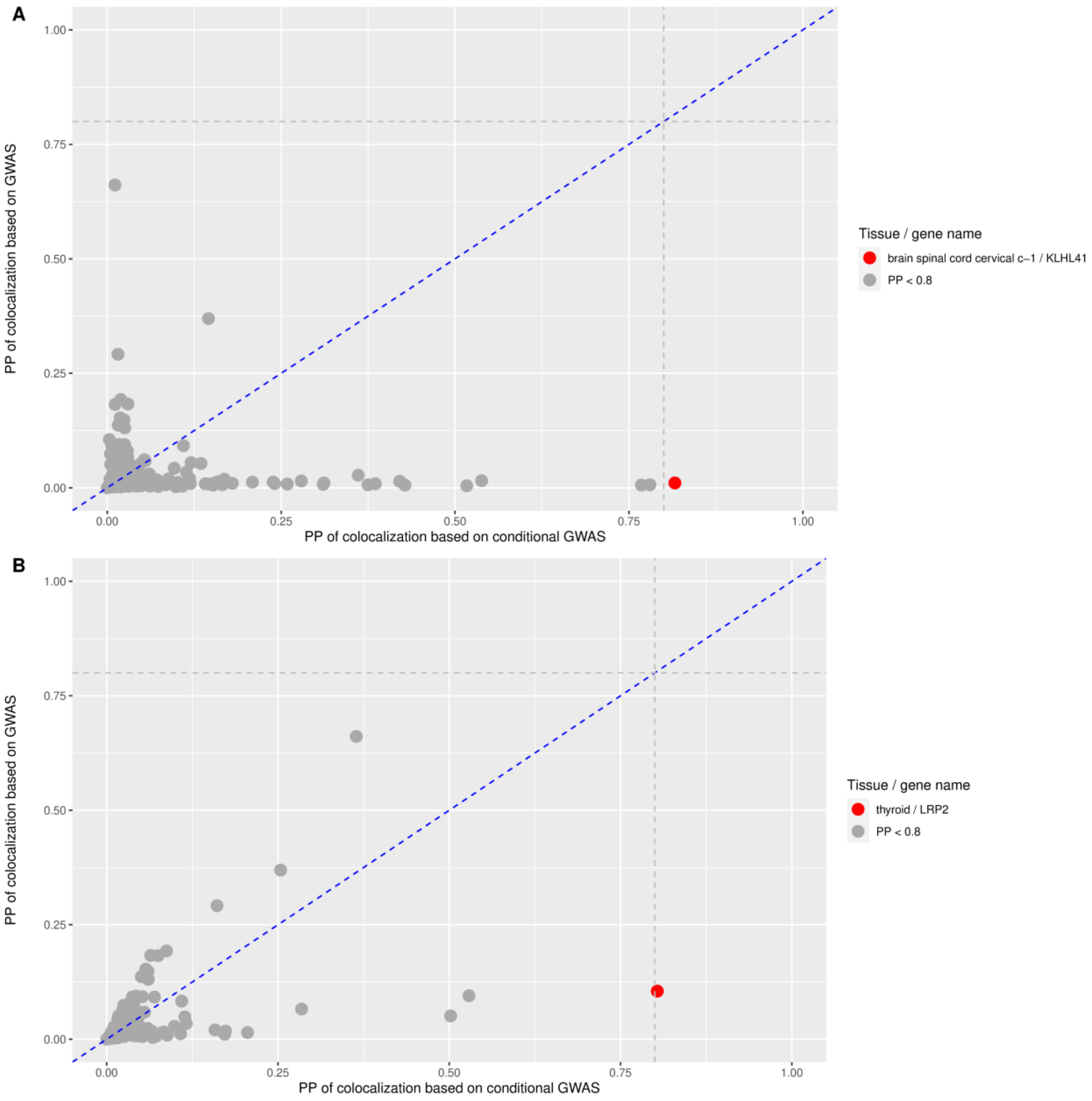

Figure 61: Colocalization results for index SNP rs807624 and tertiary signal rs807574 on chromosome 2. **Plot A**: conditional eGFR association analysis adjusted for secondary signal (rs4441471) and tertiary signal (rs807574). **Plot B**: conditional eGFR association analysis adjusted for index SNP (rs807624) and secondary signal (rs4441471).

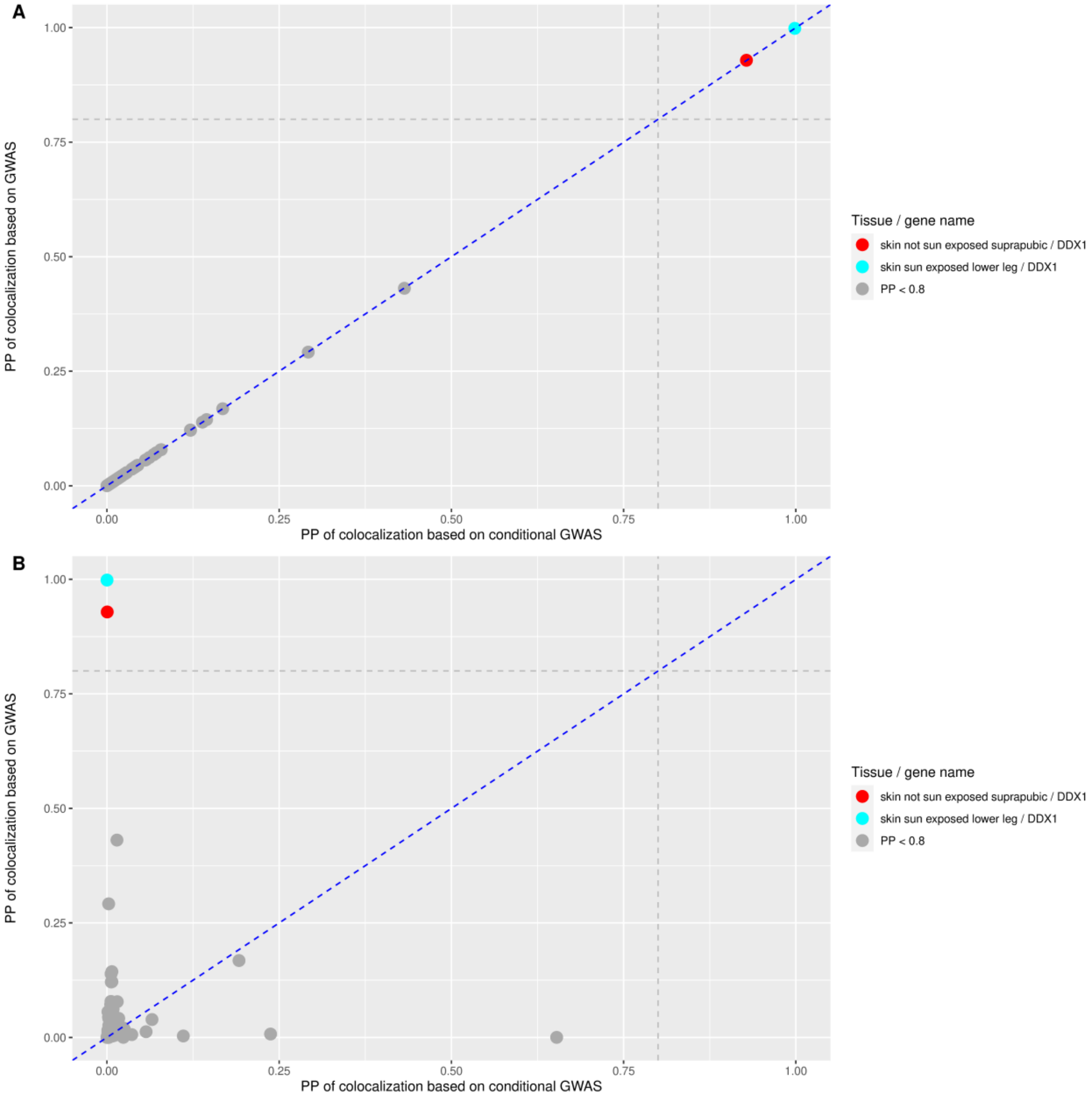

Figure 62: Colocalization results for index SNP rs1397764 and secondary signal rs147877018 on chromosome 3. **Plot A**: conditional eGFR association analysis adjusted for secondary signal (rs147877018). **Plot B**: conditional eGFR association anaylsis adjusted for index SNP (rs1397764).

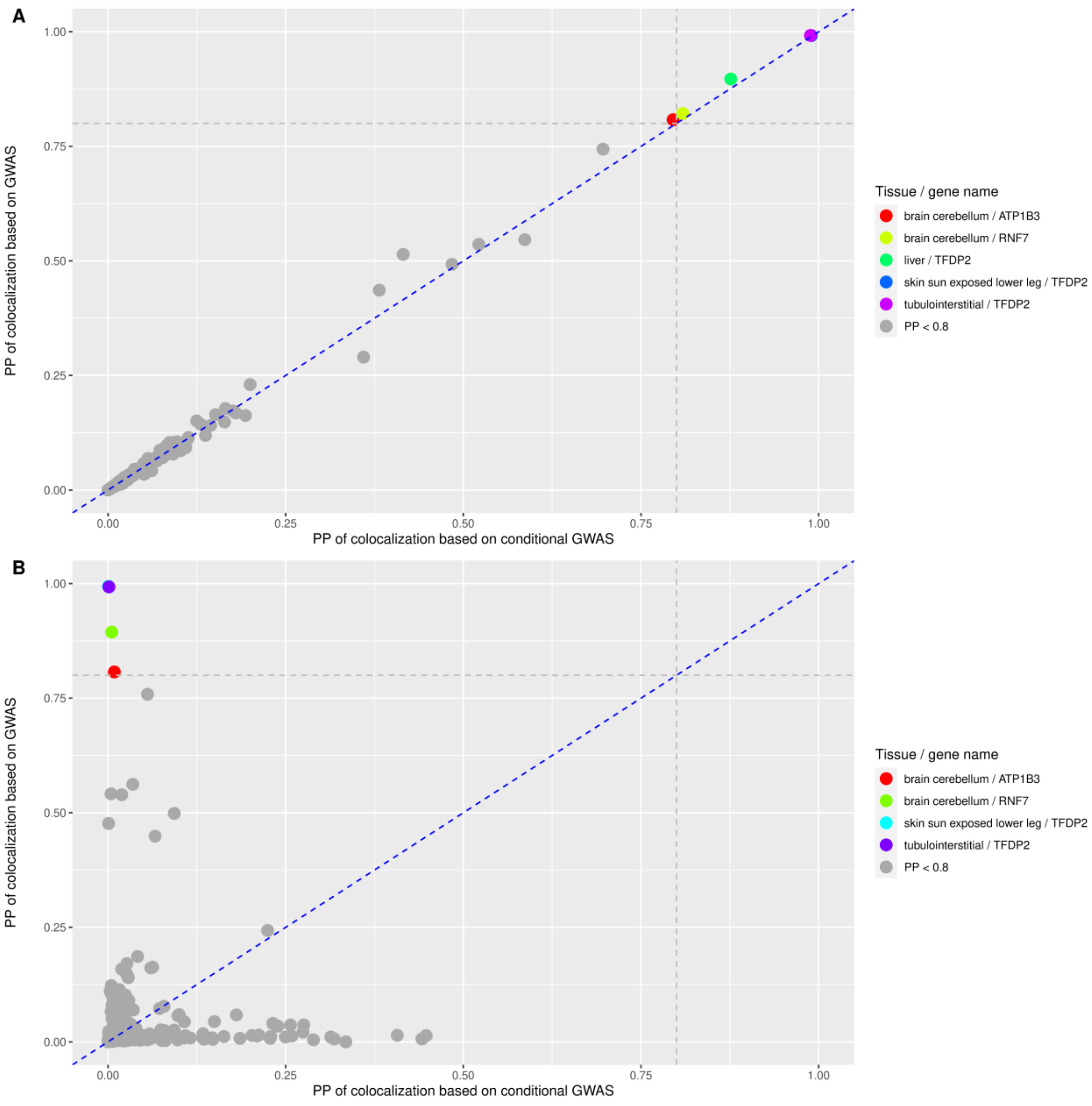

Figure 63: Colocalization results for index SNP rs12207180 and signal of 5th rs76426793 on chromosome 6. **Plot A**: conditional GWAS adjusted for secondary signal (rs316010), tertiary signal (rs9457825), signal of 4th (rs3798167), and signal of 5th (rs76426793). **Plot B**: conditional GWAS adjusted for index SNP (rs12207180), secondary signal (rs316010), tertiary signal (rs9457825), and signal of 4th (rs3798167).

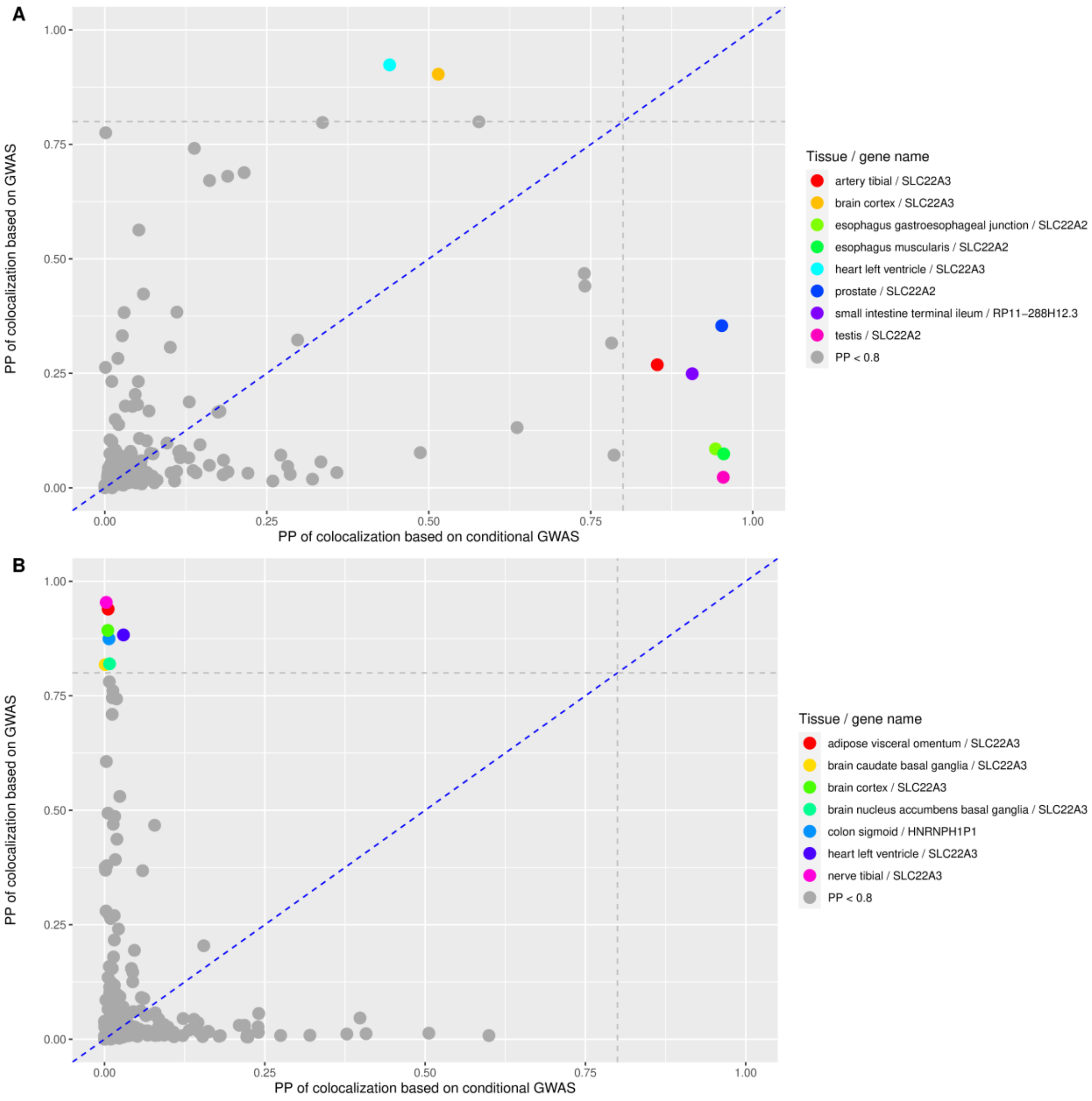

Figure 64: Colocalization results for index SNP rs6921580 and secondary signal rs3904600 on chromosome 6. **Plot A**: conditional eGFR association analysis adjusted for secondary signal (rs3904600). **Plot B**: conditional eGFR association analysis adjusted for index SNP (rs6921580).

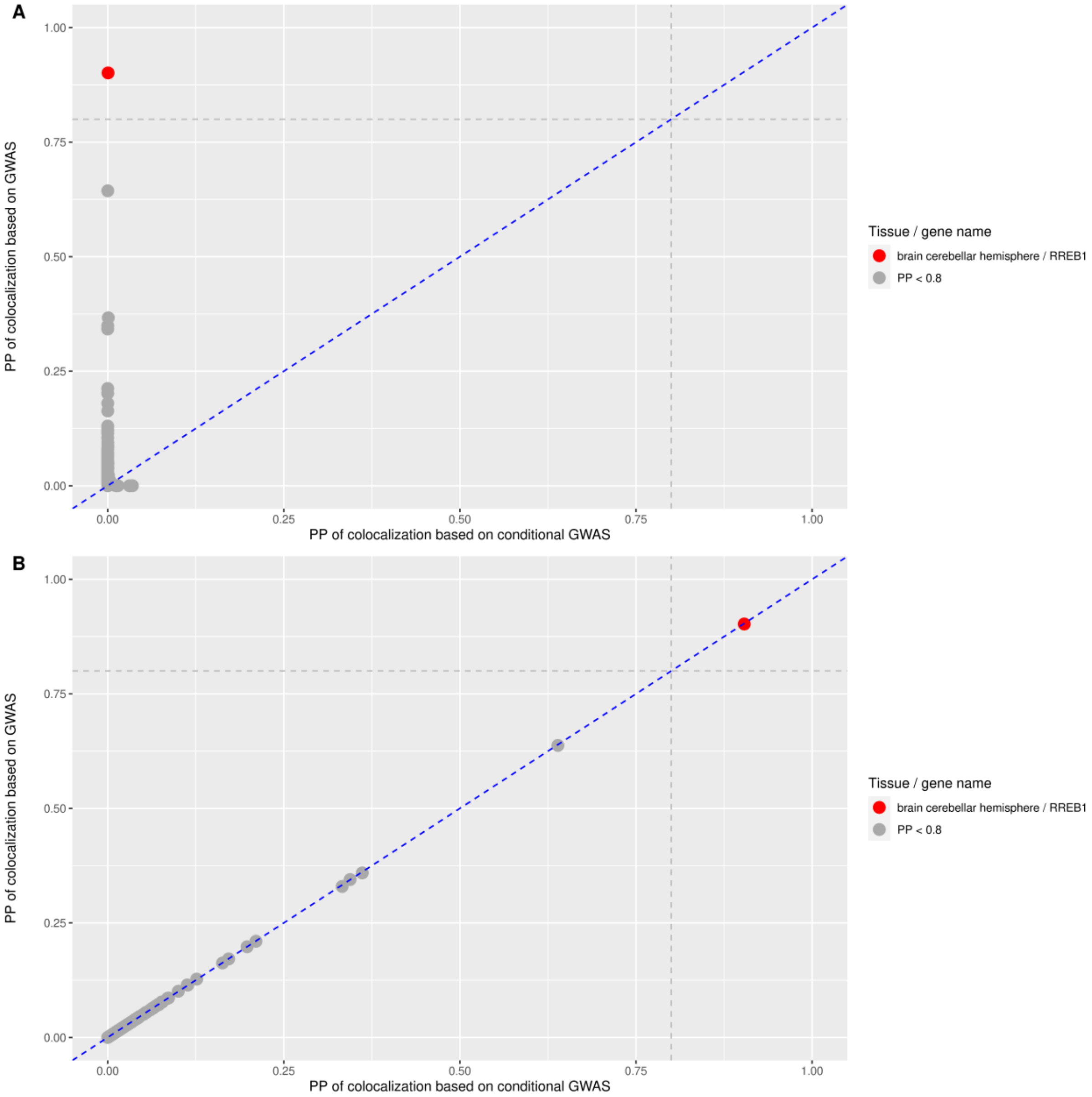

Figure 65: Colocalization results for index SNP rs881858 and signal of 4th rs6912283 on chromosome 6. **Plot A**: conditional GWAS adjusted for secondary signal (rs10498755), tertiary signal (rs833805), and signal of 4th (rs6912283). **Plot B**: conditional GWAS adjusted for index SNP (rs881858), secondary signal (rs10498755), and tertiary signal (rs833805).

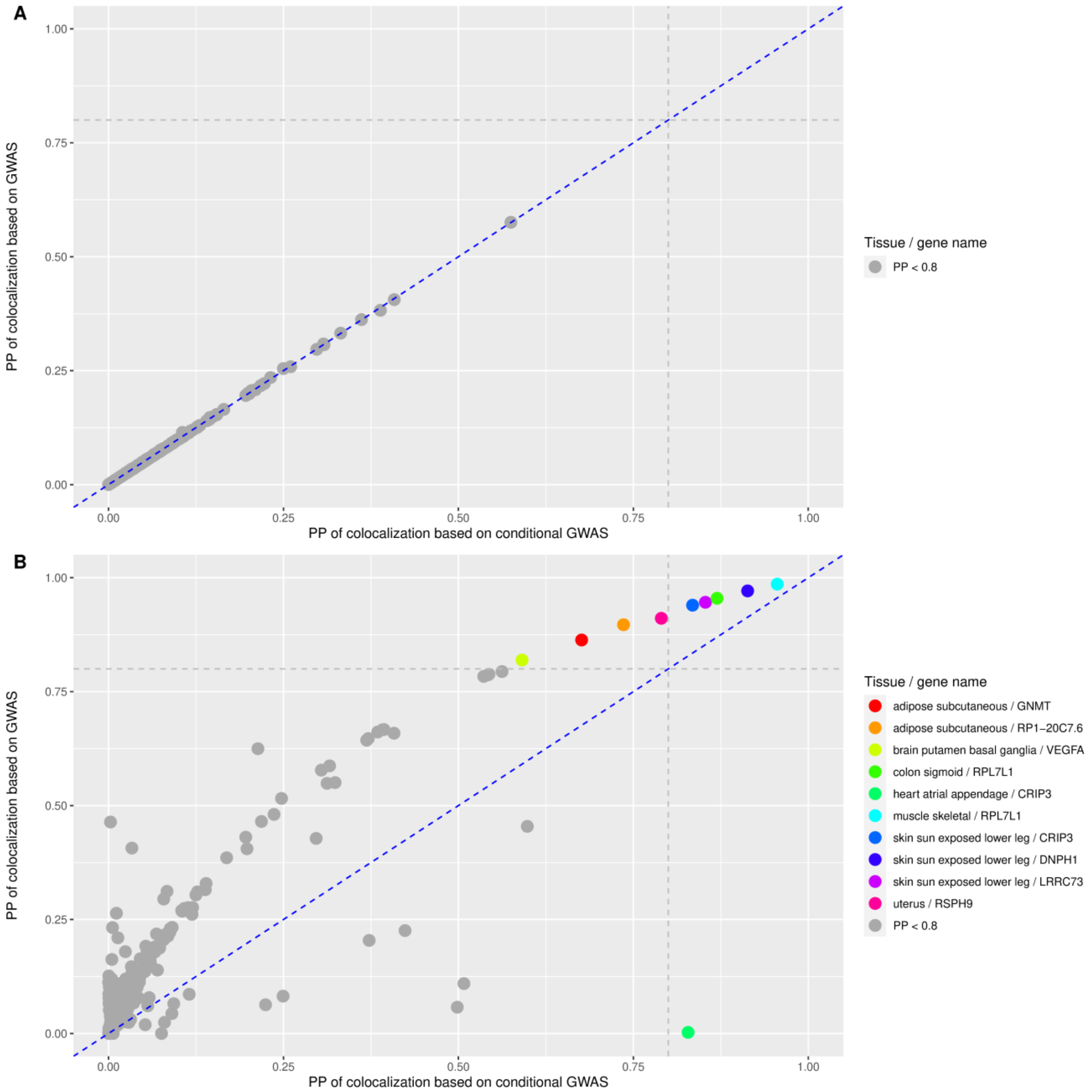

Figure 66: Colocalization results for index SNP rs2365286 and signal of 6th rs6951593 on chromosome 7. **Plot A**: conditional GWAS adjusted for secondary signal (rs6971211), tertiary signal (rs117027107), signal of 4th (rs6976733), signal of 5th (rs2695565), and signal of 6th (rs6951593). **Plot B**: conditional GWAS adjusted for index SNP (rs2365286), secondary signal (rs6971211), tertiary signal (rs117027107), signal of 4th (rs6976733), and signal of 5th (rs2695565).

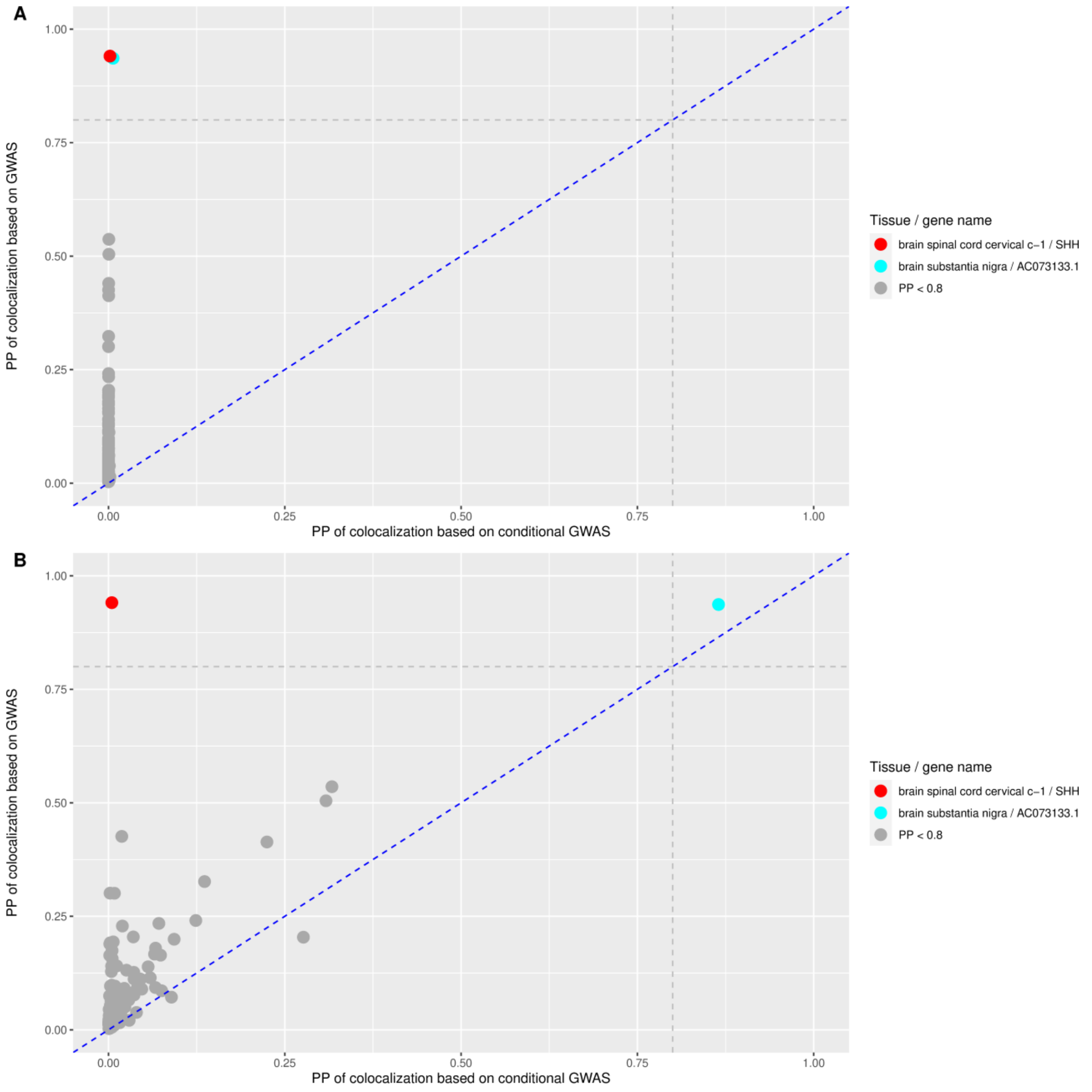

Figure 67: Colocalization results for index SNP rs3757387 and tertiary signal rs13227214 on chromosome 7. **Plot A**: conditional eGFR association analysis adjusted for secondary signal (rs62491533) and tertiary signal (rs13227214). **Plot B**: conditional eGFR association analysis adjusted for index SNP (rs3757387) and secondary signal (rs62491533).

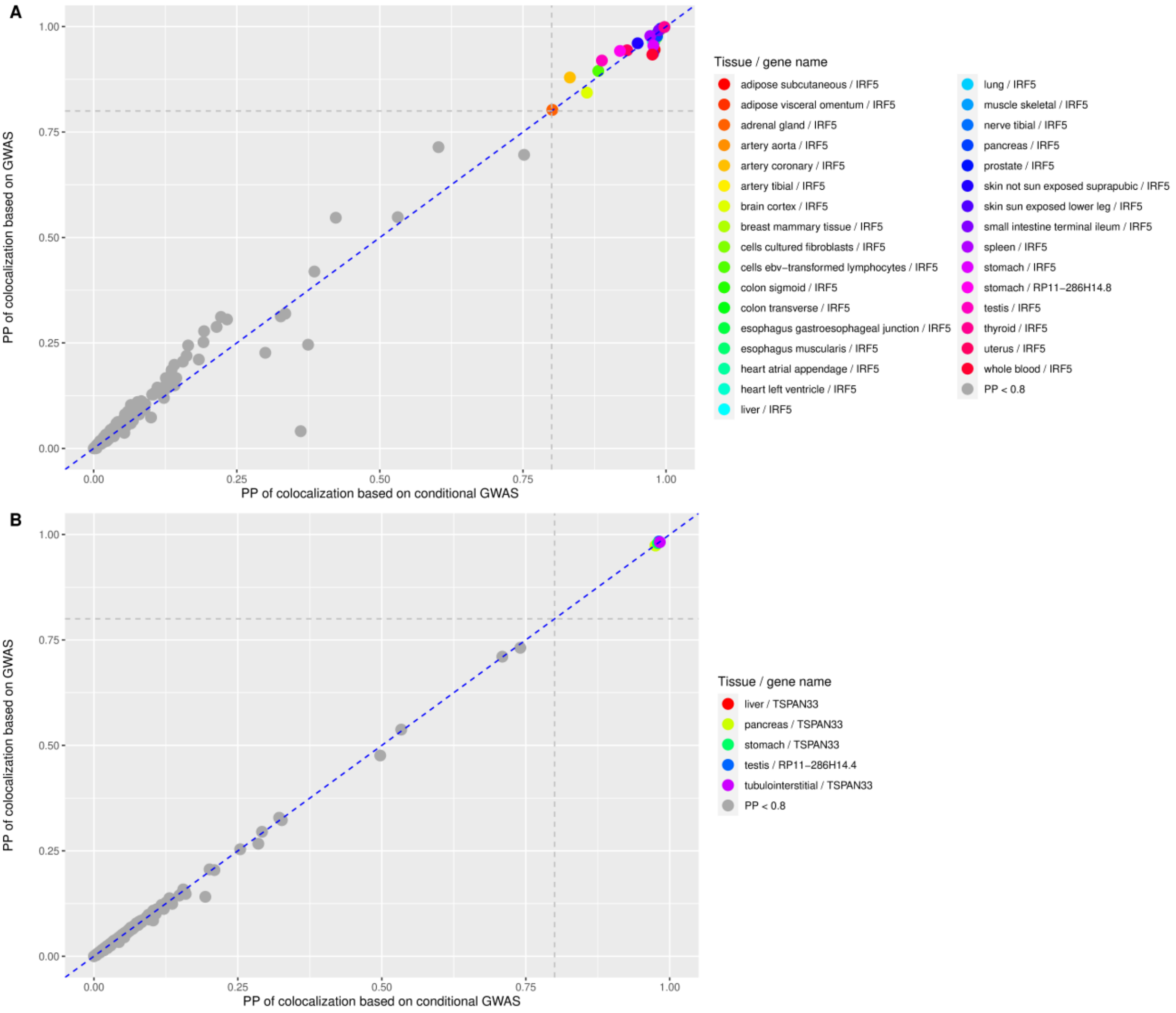

Figure 68: Colocalization results for index SNP rs10086569 and secondary signal rs4566 on chromosome 8. **Plot A**: Conditional GWAS adjusted for secondary signal (rs4566). **Plot B**: conditional GWAS adjusted for index SNP (rs10086569).

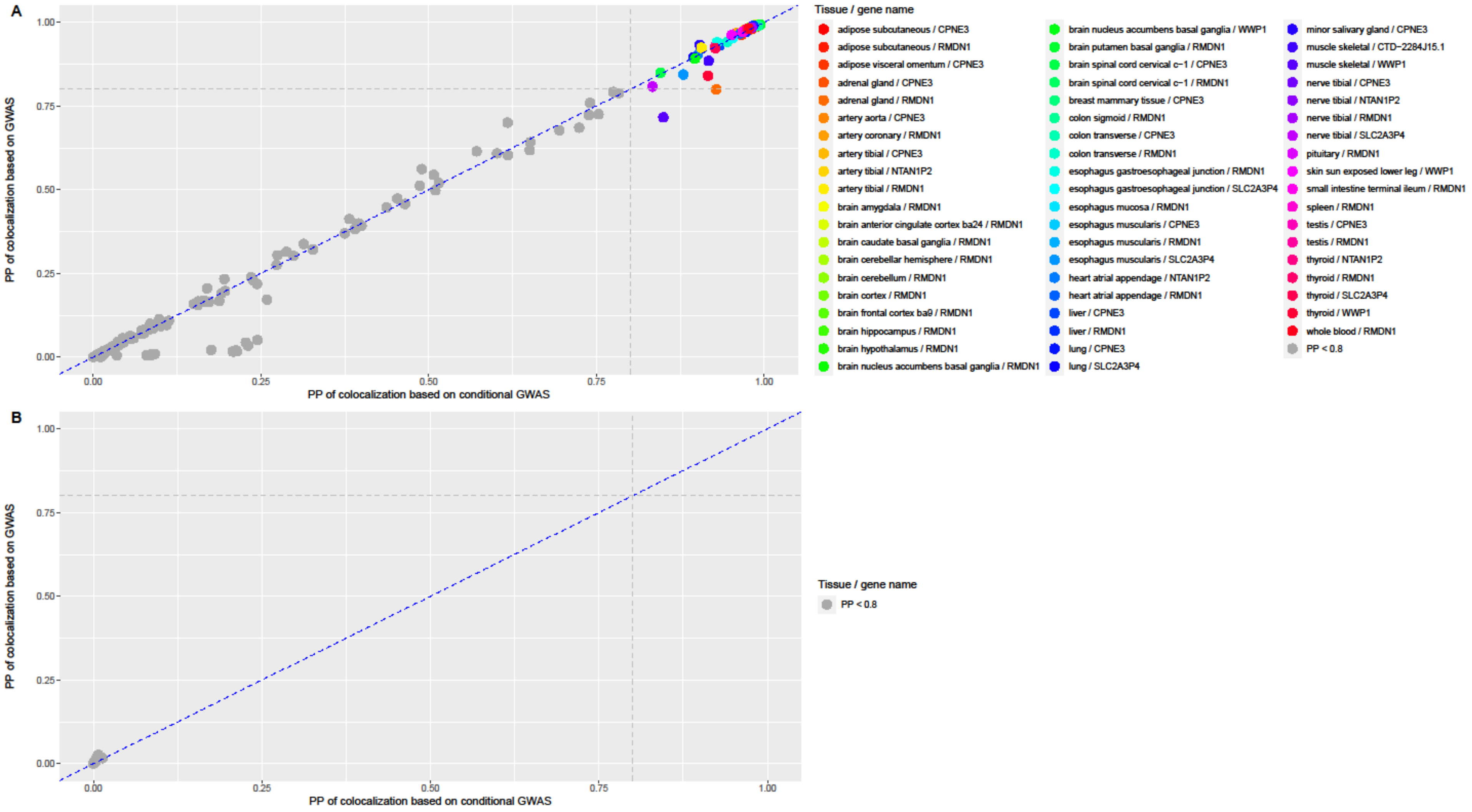

Figure 69: Colocalization results for index SNP rs2039424 and signal of 4th rs4745268 on chromosome 9. **Plot A**: conditional GWAS adjusted for secondary signal (rs7019089) and signal of 4th (rs4745268). **Plot B**: conditional GWAS adjusted for index SNP (rs2039424) and secondary signal (rs7019089).

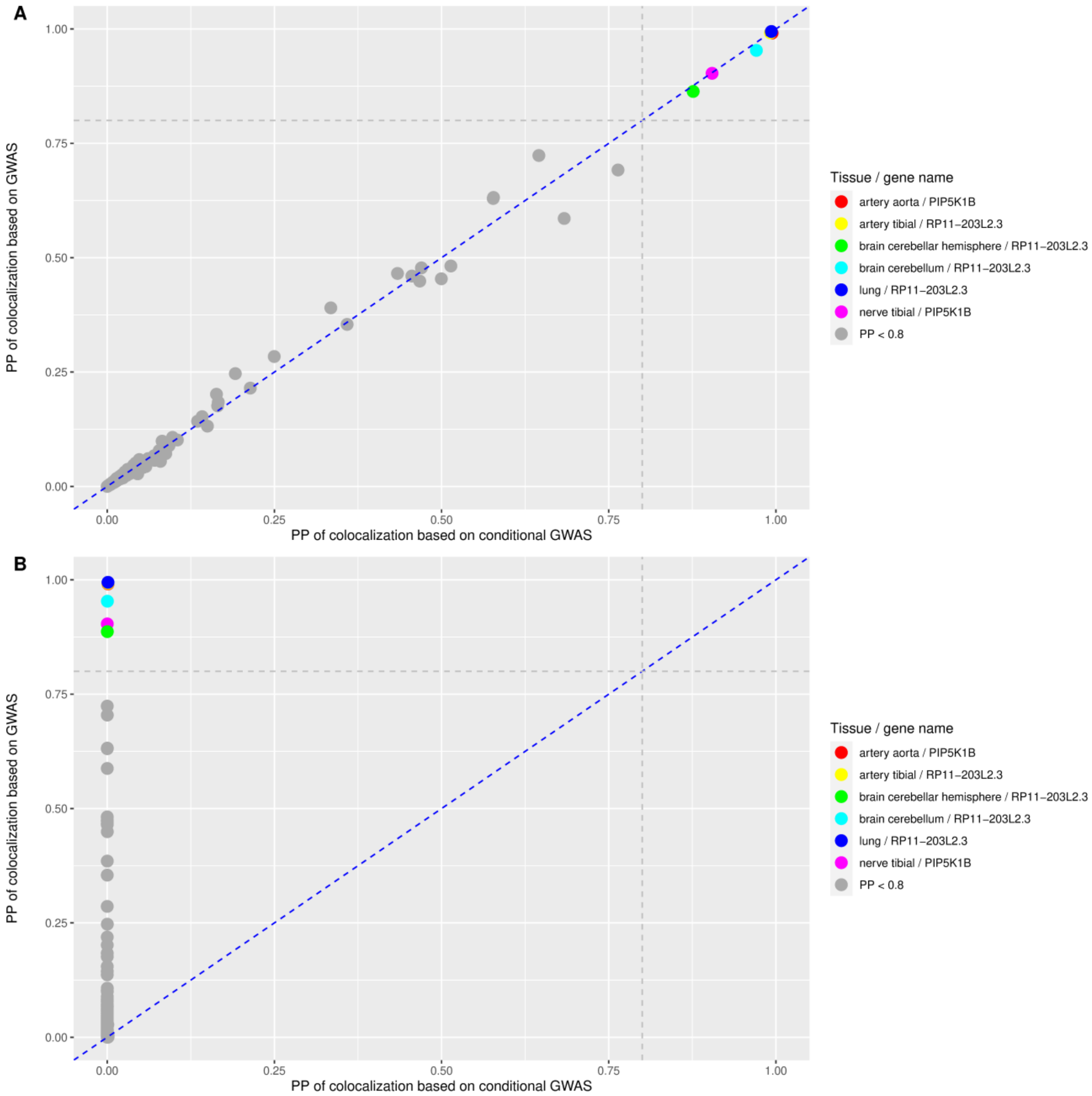

Figure 70: Colocalization results for index SNP rs11227260 and tertiary signal rs1193692 on chromosome 11. **Plot A**: conditional eGFR association analysis adjusted for secondary signal (rs34400381) and tertiary signal (rs1193692). **Plot B**: conditional eGFR association analysis adjusted for index SNP (rs11227260) and secondary signal (rs34400381).

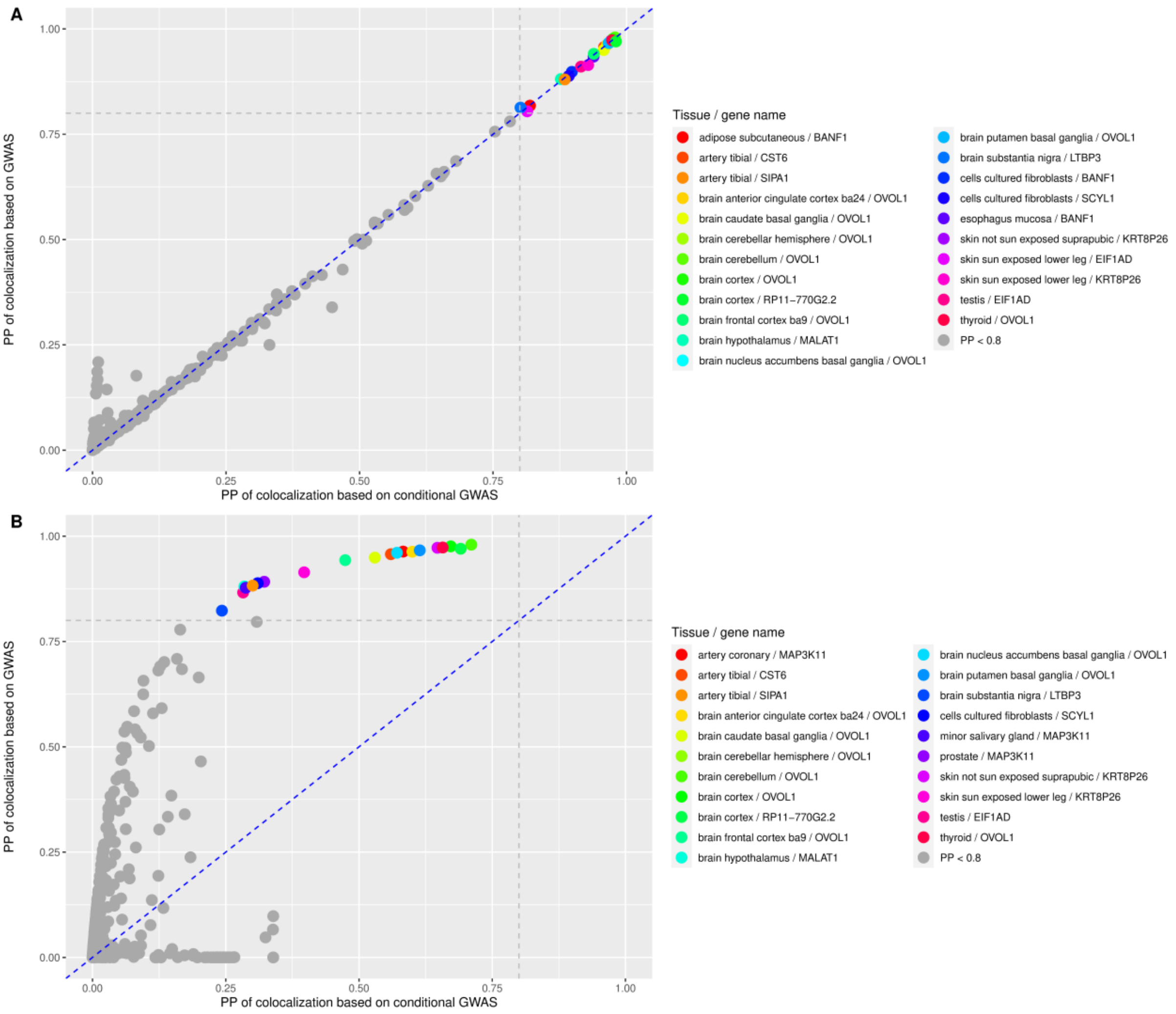

Figure 71: Colocalization results for index SNP rs233438 and signal of 4th rs1056819 on chromosome 11. **Plot A**: conditional GWAS adjusted for secondary signal (rs11564722), tertiary signal (rs81205) and signal of 4th (rs1056819). **Plot B**: conditional GWAS adjusted for index SNP (rs233438), secondary signal (rs11564722), and tertiary signal (rs81205).

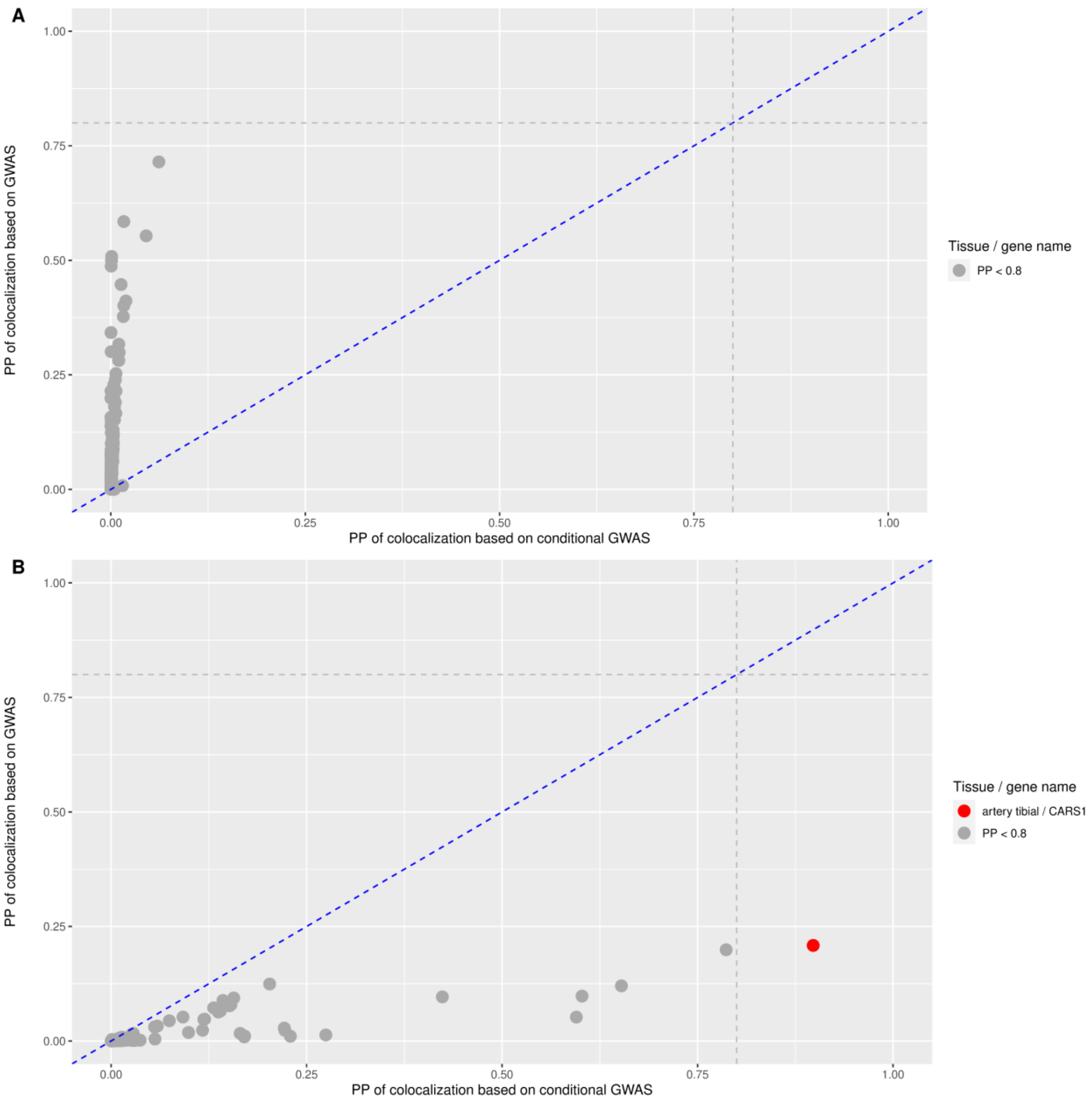

Figure 72: Colocalization results for index SNP rs233438 and tertiary signal rs81205 respectively on chromosome 11. **Plot A**: conditional eGFR association analysis adjusted for secondary signal (rs11564722), tertiary signal (rs81205) and signal of 4th (rs1056819). **Plot B**: conditional eGFR association analysis adjusted for index SNP (rs233438), secondary signal (rs11564722), and signal of 4th (rs1056819).

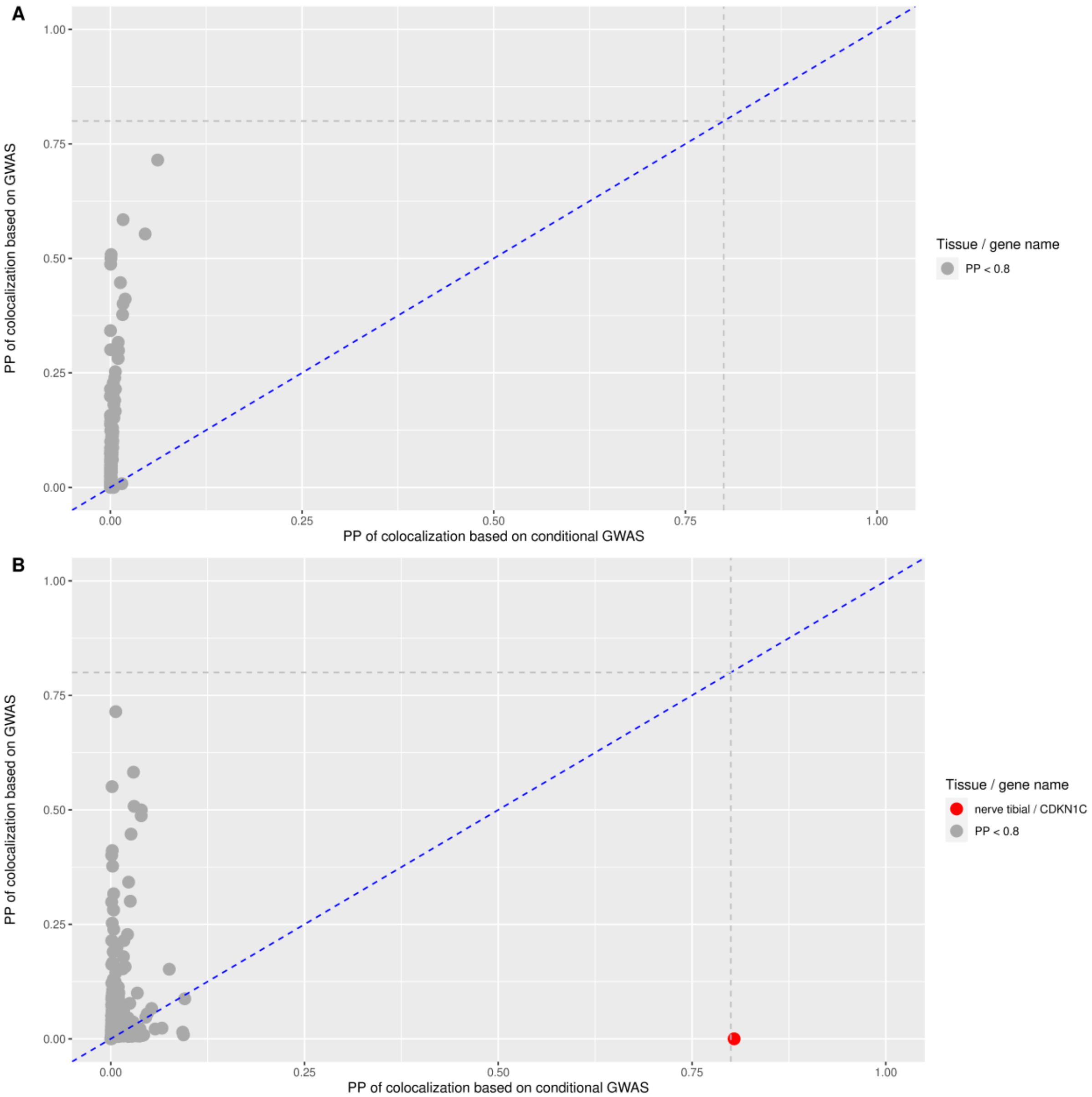

Figure 73: Colocalization results for index SNP rs2634675 and secondary signal rs3730071 on chromosome 12. **Plot A**: conditional GWAS adjusted for secondary signal (rs3730071). **Plot B**: conditional GWAS adjusted for index SNP (rs2634675).

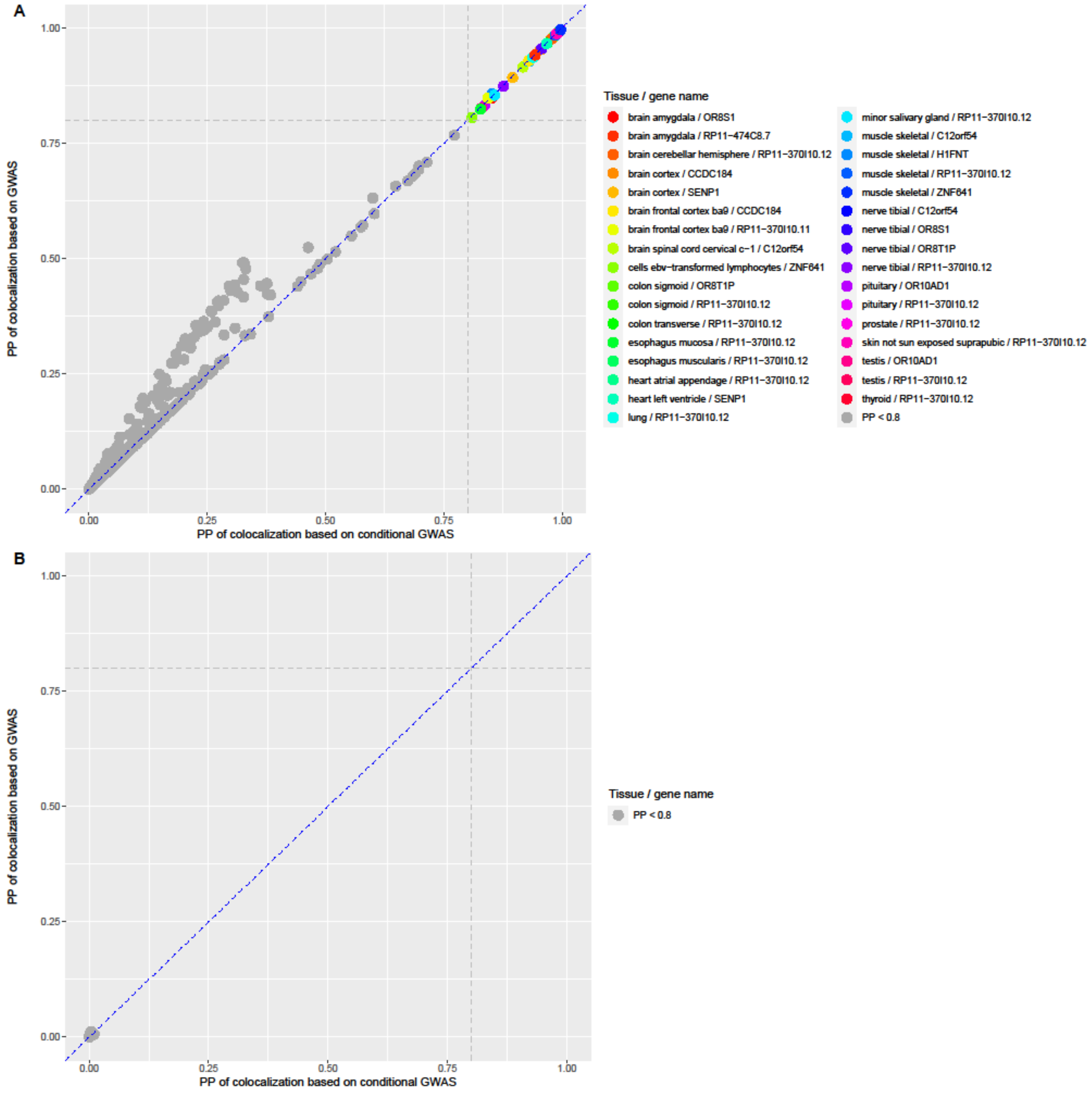

Figure 74: Colocalization results for index SNP rs1153855 and tertiary signal rs4775830 on chromosome 15. **Plot A**: conditional eGFR association analysis adjusted for secondary signal (rs68087211) and tertiary signal (rs4775830). **Plot B**: conditional eGFR association analysis adjusted for index SNP (rs1153855) and secondary signal (rs68087211).

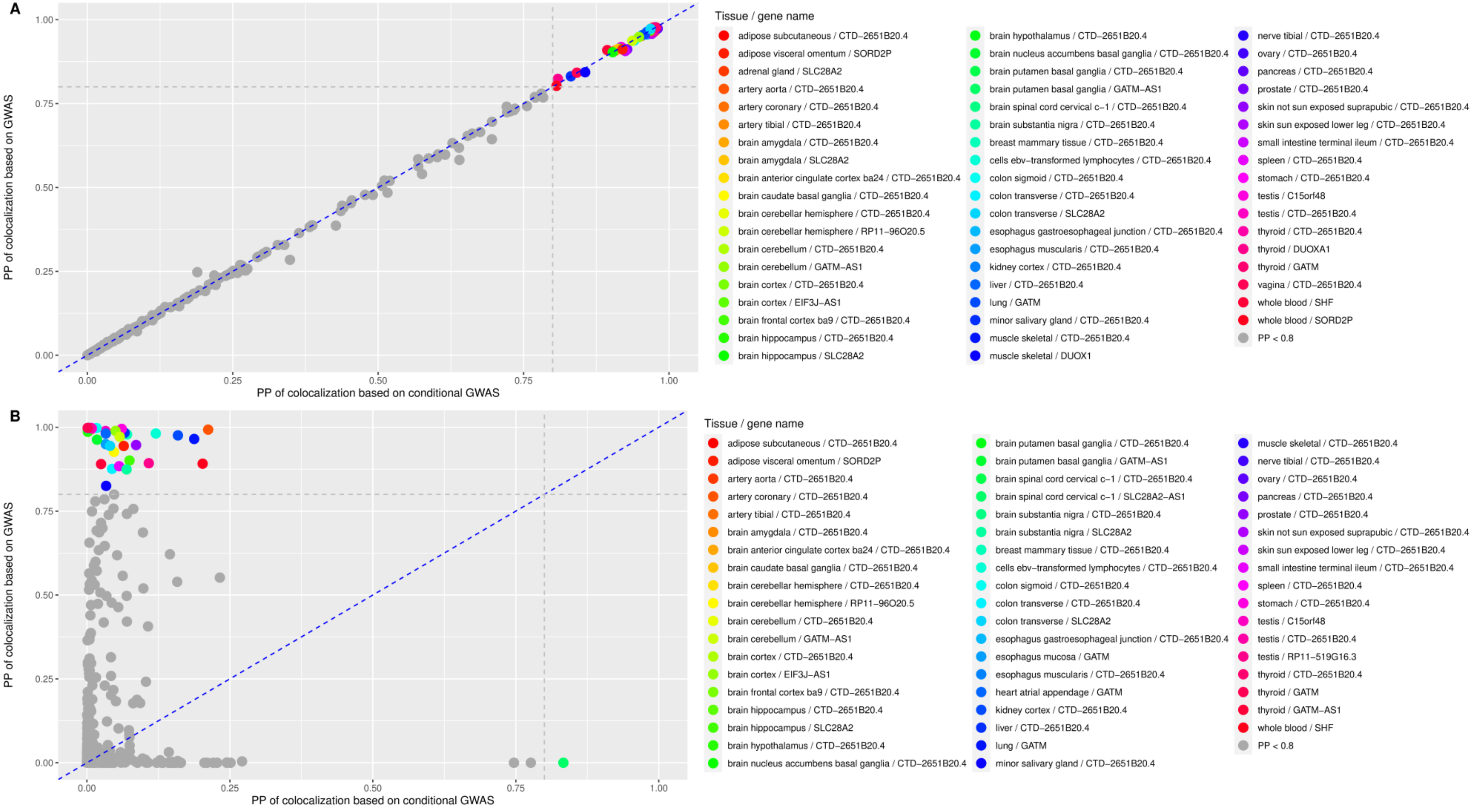

Figure 75: Colocalization results for index SNP rs2261092 and signal of 4th rs6062357 on chromosome 20. **Plot A**: conditional GWAS adjusted for secondary signal (rs4408777), tertiary signal (rs72629024) and signal of 4th (rs6062357). **Plot B**: conditional eGFR association analysis adjusted for index SNP (rs2261092) secondary signal (rs4408777) and tertiary signal (rs72629024)

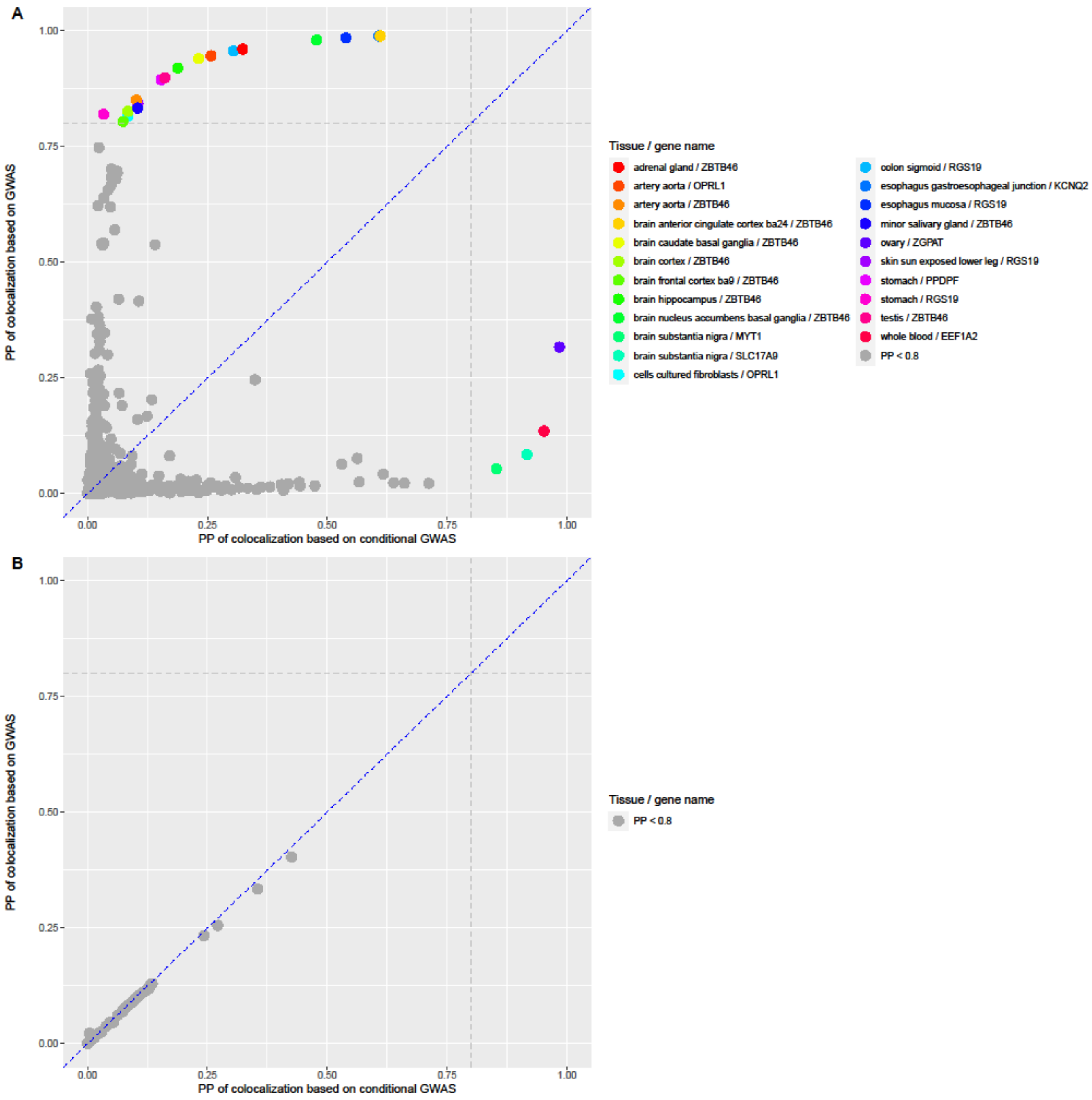

Figure 76: Colocalization results for index SNP rs6127099 and signal of 4th rs2585441 on chromosome 20. **Plot A**: conditional GWAS adjusted for secondary signal (rs2762943) and signal of 4th (rs2585441). **Plot B**: conditional GWAS adjusted for index SNP (rs6127099) and secondary signal (rs2762943)

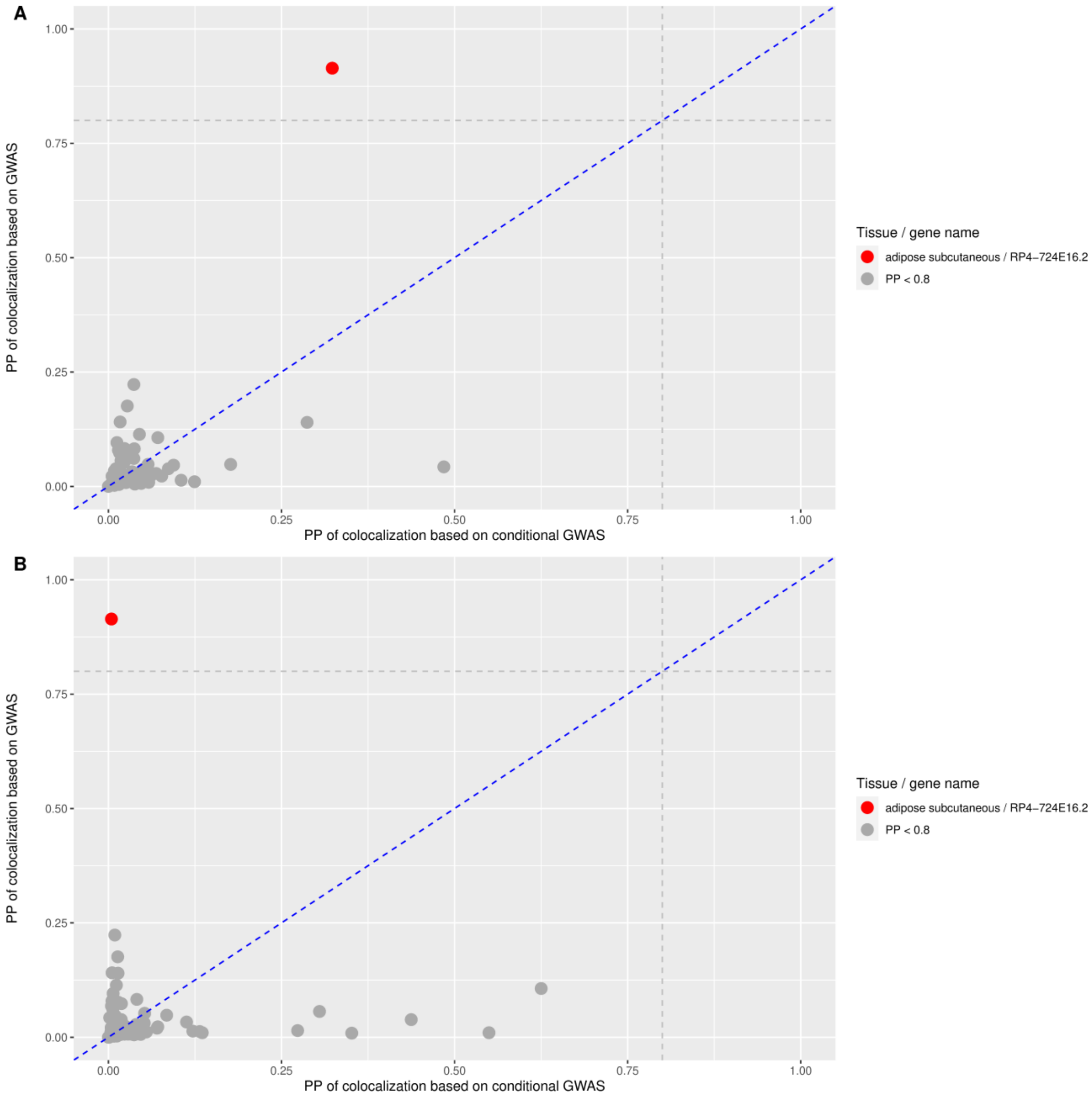

Supplement: Supplementary file 1 [file DataSheet1.pdf]
